# Soluble Diphenylhexatriene Dimers for Intramolecular Singlet Fission with High Triplet Energy

**DOI:** 10.1021/jacs.2c12060

**Published:** 2023-01-23

**Authors:** Oliver Millington, Stephanie Montanaro, Anastasia Leventis, Ashish Sharma, Simon A. Dowland, Nipun Sawhney, Kealan J. Fallon, Weixuan Zeng, Daniel G. Congrave, Andrew J. Musser, Akshay Rao, Hugo Bronstein

**Affiliations:** † Department of Chemistry, University of Cambridge, CambridgeCB2 1EW, U.K.; ‡ Cavendish Laboratory, University of Cambridge, CambridgeCB3 0HE, U.K.; § Department of Chemistry and Chemical Biology, Cornell University, Baker Laboratory, Ithaca, New York14853, United States

## Abstract

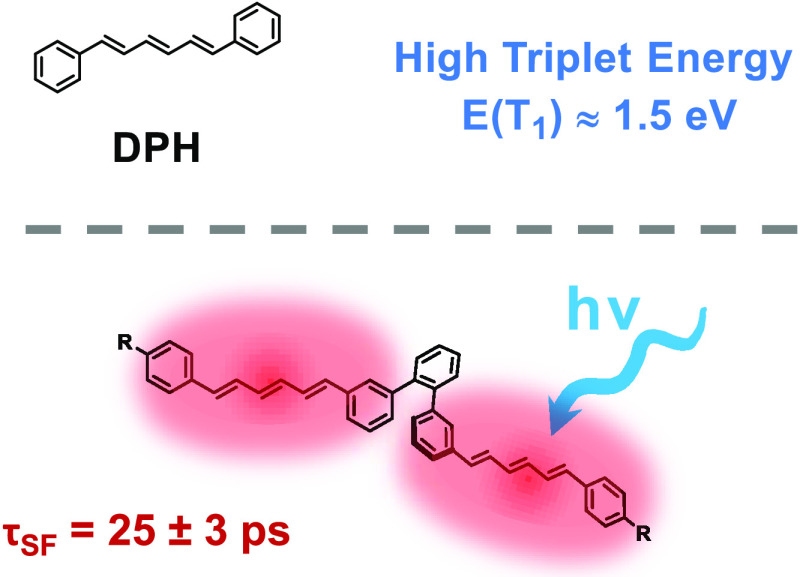

Intramolecular singlet fission (iSF) facilitates single-molecule exciton multiplication, converting an excited singlet state to a pair of triplet states within a single molecule. A critical parameter in determining the feasibility of SF-enhanced photovoltaic designs is the triplet energy; many existing iSF materials have triplet energies too low for efficient transfer to silicon via a photon multiplier scheme. In this work, a series of six novel dimers based upon the high-triplet-energy, SF-active chromophore, 1,6-diphenyl-1,3,5-hexatriene (DPH) [*E*(T_1_) ∼ 1.5 eV], were designed, synthesized, and characterized. Transient absorption spectroscopy and fluorescence lifetime studies reveal that five of the dimers display iSF activity, with time constants for singlet fission varying between 7 ± 2 ps and 2.2 ± 0.2 ns and a high triplet yield of 163 ± 63% in the best-performing dimer. A strong dependence of the rate of fission on the coupling geometry is demonstrated. For optimized iSF behavior, close spatial proximity and minimal through-bond communication are found to be crucial for balancing the rate of SF against the reverse recombination process.

## Introduction

Singlet fission (SF) is heralded for its potential to enable photovoltaic cells to surpass the Shockley–Queisser efficiency limit.^1−3^ In the simplest model for SF, an excited singlet state (S_1_) interacts with a chromophore in the ground state (S_0_) to form a correlated triplet pair state (TT) that then dissociates to form two triplets (T_1_):^4^
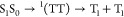
SF was first observed intermolecularly as a solid-state phenomenon within crystalline anthracene^5,6^ and has since been studied in the solid state for a range of chromophores.^7,8^ But singlet fission in crystalline systems is intrinsically dependent on morphology,^9,10^ which can be challenging to control through rational design.^11^ Intramolecular singlet fission (iSF) circumvents this problem through the combination of two or more chromophores in a single molecule, enabling synthetic control over chromophore geometry. iSF has been studied in molecular dimers, oligomers, and polymers.^12^

The acenes, particularly pentacene and tetracene, have dominated iSF studies.^7,12^ However, the relatively low triplet energies of pentacene (0.81 eV)^13^ and tetracene (1.25 eV)^14^ prevent effective incorporation into silicon photovoltaic devices. While the band gap of silicon is 1.1 eV, accounting for energetic losses in the triplet harvesting process, the ideal triplet energy of the SF component material in a photon multiplier system is 1.4–1.5 eV.^15^

Beyond the acenes, translating efficient SF in the solid state to efficient iSF in covalent dimers has been elusive for high-triplet-energy chromophores; solid 1,3-diphenylisobenzofuran (*E*(T_1_) = 1.41 eV)^16^ is capable of SF with a high triplet yield (Φ_T_) (200 ± 30%)^16^ but covalent dimers fail to exceed 10%.^17^

Diphenylhexatriene (DPH) has been reported as an efficient singlet fission chromophore,^18^ with a triplet energy of ∼1.5 eV.^19−23^ To date, no covalent dimers of DPH have been investigated for iSF, with all SF studies on DPH derivatives restricted to the crystalline domain.^9,18,24−29^

Herein, we present the first iSF study of DPH derivatives, with a systematic series of isomeric DPH dimers, linked by a phenylene bridge. Phenylene-linked dimers have demonstrated their utility in the systematic investigation of iSF behavior for the acenes^30−36^ and were chosen as a logical starting point for study toward developing structure–function relationships for iSF systems based upon DPH. Time-correlated single photon counting and transient absorption spectroscopy reveal the excited-state dynamics of these materials. Notably, iSF activity is observed in all but one of the dimers, with Φ_T_ of 163 ± 63% in the best-performing material. The timescale of iSF is observed to vary over 3 orders of magnitude, and the ability of strongly correlated triplet pairs to lose their spin correlation demonstrates a strong dependence on the molecular geometry.

## Results

### Design and Synthesis

**Scheme 1:**
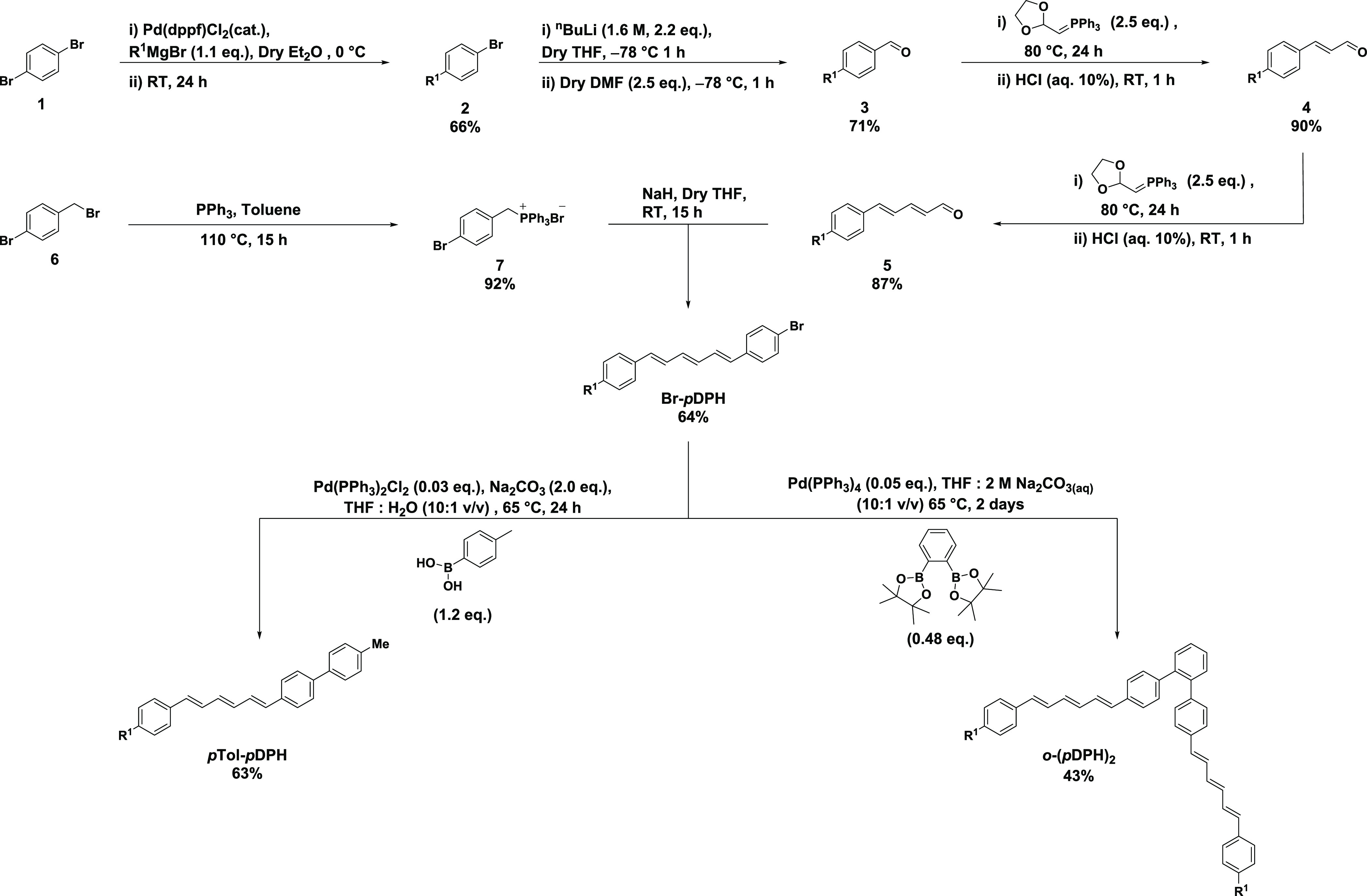
Synthetic Scheme of *p*Tol-*p*DPH and *o*-(*p*DPH)_2_

A new methodology for the synthesis of asymmetric DPH derivatives was developed, to synthesize a bromo-substituted DPH building block capable of being dimerized via cross-coupling. In consideration of the poor solubility of DPH, a branched alkyl chain was incorporated with the aim of enhancing the solubility of the final materials. The building block was coupled via a Suzuki reaction with corresponding commercial bis-boronic acid pinacol esters or *p*-tolyl boronic acid to produce the final materials, as illustrated for representative examples in Scheme 1.

**Figure 1:**
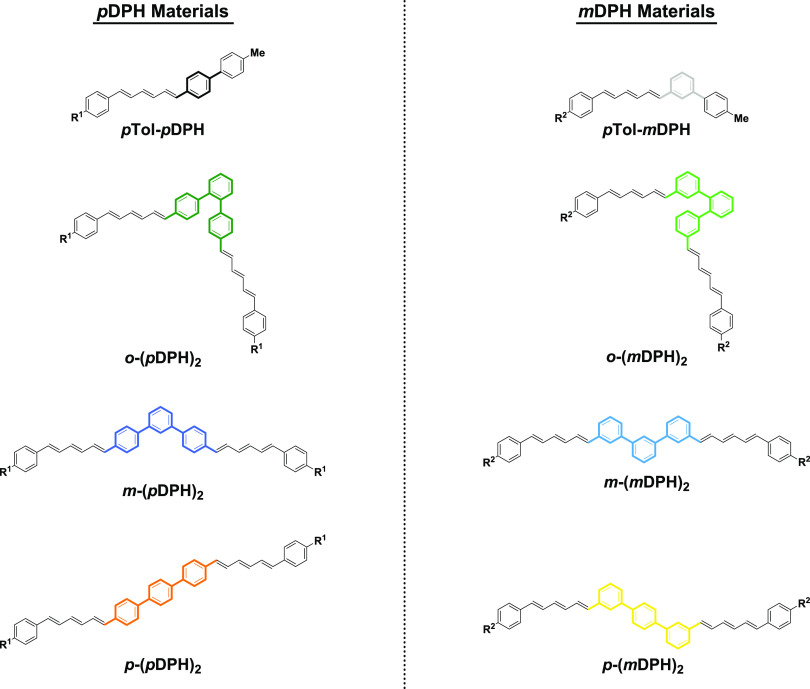
Chemical structures of the DPH dimers and reference materials studied in this work. The geometry defining the central region is highlighted in bold with color. R groups indicate branched alkyl chains incorporated for enhancing solubility: R^1^ = 2-butyloctyl and R^2^ = 2-ethylhexyl.

In total, a series of six isomeric DPH dimers were synthesized, linked via a phenylene spacer (Figure 1). The terminal phenyl rings of DPH provide multiple degrees of freedom for dimer connectivity. First, there is the geometry of the phenylene linker, which here was varied through the *ortho*, *meta*, and *para* substitution arrangements, noted by the prefix *o/m/p*. Second, the geometry of the phenylene rings that attach to the linker is significant. For each of the three bridge geometries, we have investigated *para* and *meta* relationships between the hexatriene and the linker and adopted the terms “*p*DPH” or “*m*DPH” to refer to the DPH unit in each family of materials.

In addition to the six dimers, a reference monomeric DPH material was synthesized for each of the *p*DPH and *m*DPH families. These were designed to better represent the monomeric unit of the dimers than DPH itself, through a tolyl substituent in the place of the linking phenylene of the dimers.

### Steady-State Absorption and Photoluminescence

**Figure 2:**
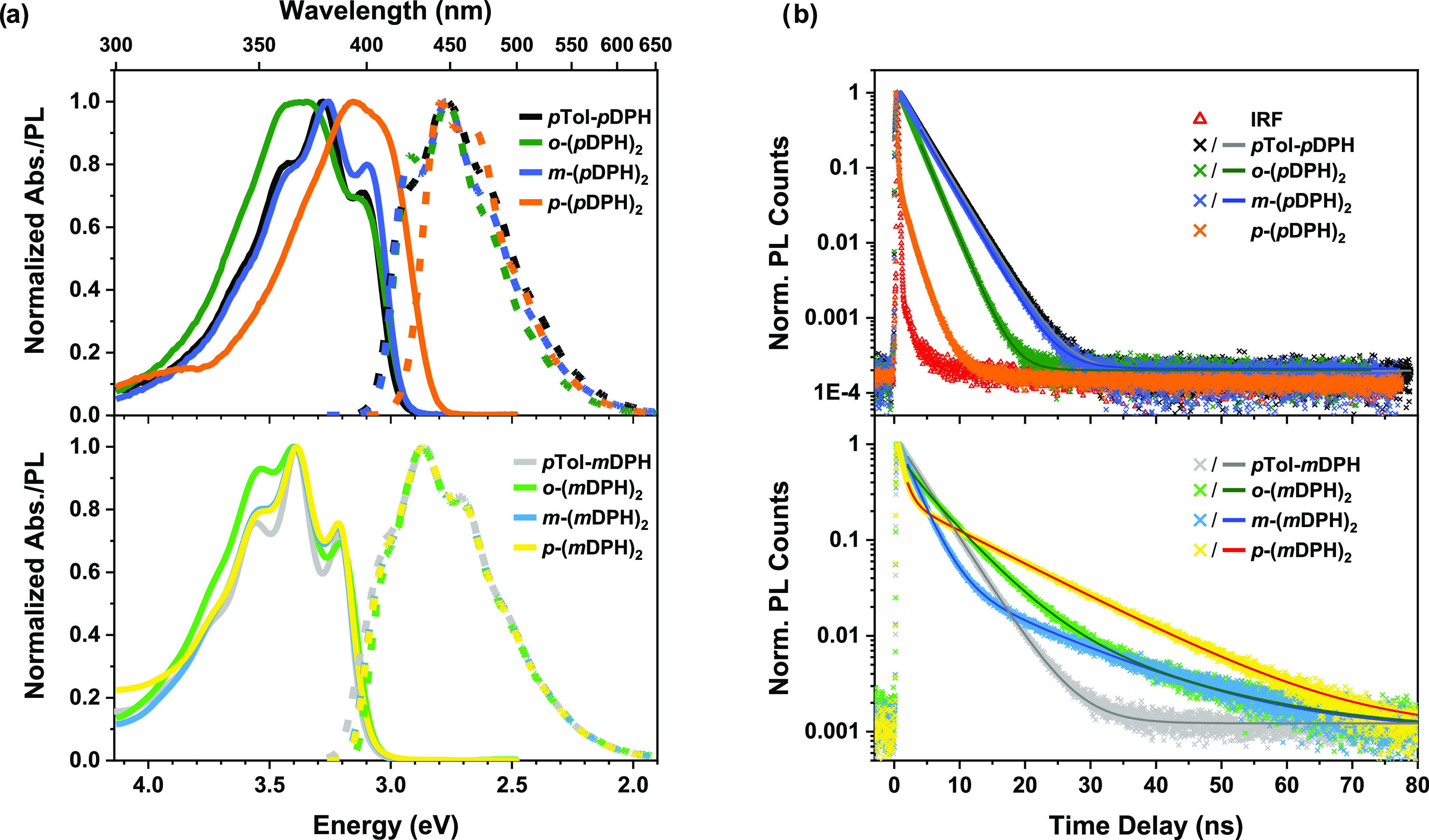
(a) UV–vis absorption (solid line) and photoluminescence (dashed line) spectra of dilute solutions (∼5 to 10 μM) in toluene. Photoluminescence spectra were recorded with excitation at 355 nm, except for *p*-(*p*DPH)_2_ (excited at 385 nm). (b) TCSPC emission lifetime plot of dilute solutions (<̃100 μM) excited at 375 nm. Symbols represent the raw data, while lines indicate the fits to the data. The instrument response function (IRF) was measured by scattering the excitation beam off a piece of scratched glass.

The steady-state absorption and photoluminescence (PL) spectra of dilute solutions (∼ 5–10 μM) in toluene are shown in Figure 2a. Except for *p*-(*p*DPH)_2_, the dimers have absorption and PL onsets effectively equal to their respective monomers, suggesting similar singlet energetics.

Relative to reference monomer *p*Tol-*p*DPH, *p*-(*p*DPH)_2_ exhibits a bathochromic shift of *ca.* 0.15 eV. This was shown to be temperature-independent, allowing an aggregation-related origin to be ruled out (SI Figure S2). Uniquely, the all-*para* geometry enables direct electronic conjugation of the DPH units, resulting in greater delocalization and consequently a lower-energy singlet excited state.

The *m*DPH family are hypsochromically shifted by ∼0.1 eV relative to their *p*DPH counterparts (by ∼0.25 eV for the two *para* dimers). This demonstrates the influence of breaking the direct conjugation of the hexatriene unit with the phenylene linker (or tolyl end group). Thus, the *m*DPH family more closely represents the energetics of the native DPH chromophore, while the *p*DPH family are a distinctly lower-energy class of materials. Therefore, dependent upon the coupling geometry, it is possible that the linker may modulate the fundamental excited-state energetics of the chromophore in dimers designed for iSF.

The vibronic band structure of the spectra of the monomers is similar to that of native DPH, which has previously been studied in detail.^37,38^ The line shapes for *m*-(*p*DPH)_2_, *m*-(*m*DPH)_2_, and *p*-(*m*DPH)_2_ display only slight differences relative to the relevant monomers, suggesting weak vibronic coupling between the DPH units.

Meanwhile, the remaining dimers (*o*-(*m*DPH)_2_, *o*-(*p*DPH)_2_, and *p*-(*p*DPH)_2_) exhibit more significant divergences in the line shapes of their absorption spectra, potentially indicating changes in the DPH unit geometry.

### Fluorescence Lifetimes and Yields

Fluorescence lifetimes were determined by time-correlated single photon counting (TCSPC) (Figure 2b and Table 1). The fluorescence of the monomeric materials decays monoexponentially, as expected for decay from a single emissive state (or a mixture of states equilibrating on a timescale far faster than the fluorescence). The fluorescence lifetime of *p*Tol-*m*DPH is intermediate between that of *p*Tol-*p*DPH and the literature value reported for native DPH (5.3 ns).^39^ Similar variation in fluorescence lifetime is well documented across other DPH derivatives, all with similar fluorescence yields (∼70 to 80%).^40^ Variations in lifetime are attributed to the interaction between the S_2_ and S_1_ states, which are nearly degenerate in DPH and other polyenes.^41−43^ The S_2_ (1B_u_) state is the state for which an optical transition from the ground state (S_0_) is formally allowed. Internal conversion enables very rapid equilibration with a slightly lower-lying state S_1_ (2A_g_). Fluorescence from S_1_ is enabled via a Herzberg–Teller coupling to a vibrational mode with odd symmetry. As such, a smaller S_2_–S_1_ gap corresponds to a faster radiative rate.^40^ It is evident that changing the position of the tolyl group from *meta* to *para* relative to the hexatriene in our materials significantly affects this interaction. Indeed, the noted ∼0.1 eV difference in absorption onset suggests a lowering of the S_2_ state that may not necessarily be equaled by a change in energy of the S_1_ state.

**Table 1:** Fluorescence Yields and Lifetimes (<̃100 μM)

compound	PLQE (%)	τ(τ_1_; τ_2_; τ_3_) (ns)	relative amplitudes (A_1_; A_2_; A_3_)
*p*Tol-*p*DPH	84	3.0	
*o*-(*p*DPH)_2_	48	2.1	
*m*-(*p*DPH)_2_	85	2.9	
*p*-(*p*DPH)_2_	4		
*p*Tol-*m*DPH	85	4.1	
*o*-(*m*DPH)_2_	22	1.6; 5.4; 17.1	0.24; 0.71; 0.03
*m*-(*m*DPH)_2_	66	2.5; 14.4	0.96; 0.04
*p*-(*m*DPH)_2_	74	1.0; 12.5	0.81;0.19

As the majority (>90%) of the decay of *p*-(*p*DPH)_2_ occurs within the instrument response time of the TCSPC setup, a valid lifetime cannot be obtained. While the other materials all have moderate photoluminescence quantum yield (PLQE), the PLQE of *p*-(*p*DPH)_2_ is very low (4%), indicating this rapid excitation decay arises from a fast nonradiative process. The direct conjugation in *p*-(*p*DPH)_2_ may impart properties similar to those of a longer polyene, where the relationship of decreasing singlet lifetime with increasing polyene length is well known and attributed to faster internal conversion to the ground state.^44^ In contrast, *m*-(*p*DPH)_2_ has both a fluorescence lifetime and PLQE similar to that of the relevant monomer, suggesting that the rates of both radiative and nonradiative decay processes are not significantly impacted by the second DPH unit.

While the *p*DPH materials exhibit monoexponential fluorescence decay, the *m*DPH dimers demonstrate distinctly different decays, which were best fitted by biexponential or triexponential functions. The *m*DPH dimers exhibit decay components with longer lifetimes than the reference monomer, indicating at least one additional state, beyond the singlet, must be involved. This state(s) is either capable of emission itself or able to convert back into the singlet state. As the line shape of the PL spectra are closely matching with the reference monomer (Figure 2a), if the delayed emission is from a distinct state, then it is not resolved under steady-state measurement conditions.

Within the concentration regime of the TCSPC experiments (10^–5^ to 10^–4^ M) and the timescale of the lifetime constants, the involvement of excimers or intermolecular process can be confidently ruled out. Hence, from the TCSPC data, it appears that the *m*DPH dimer family exhibit delayed fluorescence, requiring the involvement of one or more longer-lived states from which the singlet can be reformed. We turn toward transient absorption spectroscopy to further investigate the excited-state dynamics of the materials and potentially rationalize the delayed fluorescence component in the *m*DPH dimers.

### Nanosecond Transient Absorption(nsTA): Monomers

Before considering the dimer dynamics, it is important to discuss the excited-state dynamics in the monomeric reference materials. Nanosecond resolved transient absorption spectroscopy (nsTA) was utilized to study the monomers’ behavior in solution at various concentrations. The broad singlet photoinduced absorption (PIA) features (2.8–2.0 and <1.8 eV) are observed to decay within the first few nanoseconds.

**Figure 3:**
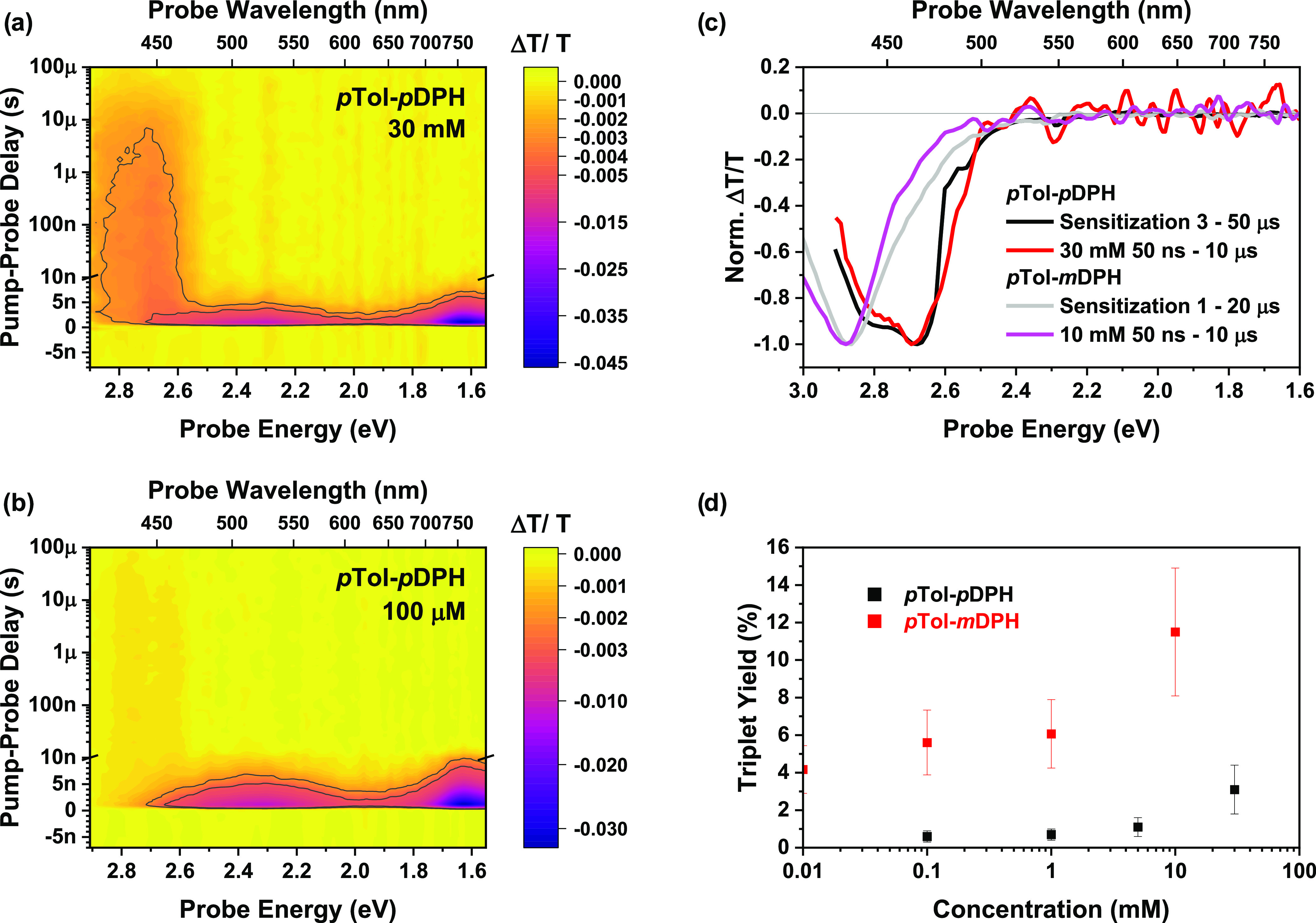
nsTA dynamics of the reference monomer materials. (a) TA contour plot of *p*Tol-*p*DPH (30 mM) with excitation at 400 nm. A higher density of contour color levels is employed below |Δ*T*/*T*| = 0.005 to highlight the long-lived feature. The gray contour lines indicate PIA intensities at ∼10% and ∼6% of the peak. (b) Same as (a) for 100 μM with a threshold of |Δ*T*/*T*| = 0.003 for the increased level density. (c) Comparison of the long-lived features in the nsTA spectra and the sensitized triplet spectrum produced by sensitization with PdOEP for both monomers. (d) Concentration dependence of the triplet quantum yields.

For *p*Tol-*p*DPH, in concentrated solutions, a long-lived PIA (2.9–2.6 eV) continuing for tens of microseconds is clearly visible (Figure 3a), while in dilute solution, this signal is very weak (Figure 3b). Triplet states in organic materials typically possess long lifetimes (>10 μs).^2,8,45^ These arise from low oscillator strengths such that transitions to the ground state are formally forbidden.^46^ Similar trends were observed for *p*Tol-*m*DPH (SI Figure S3).

Sensitization experiments were carried out with the triplet sensitizer, palladium(II) octaethylporphyrin (PdOEP), excited at 532 nm (Figure 3c). Comparison of the sensitized triplet spectra to the long-lived features in concentrated solutions of the DPH monomers confirms that the concentration-dependent long-lived species are triplet states.

The conjugation of the tolyl group with the hexatriene in *p*Tol-*p*DPH results in a distinct shift and broadening of the triplet PIA relative to *p*Tol-*m*DPH. These spectral differences are consistent across the two families with the triplet spectra of the dimers matching well to their respective monomers (SI Figures S8c and S10a).

Triplet absorption cross sections were determined from the sensitization experiments and utilized to calculate the Φ_T_ (full details in Section 5 in the SI). Both monomers display a consistently low Φ_T_ across multiple orders of magnitude of concentration but show an appreciable increase in Φ_T_ in the most concentrated case (Figure 3d).

The residual Φ_T_ at low concentration may be attributed to intersystem crossing (ISC), which is typically low yielding for small organic molecules in the absence of any heavy atoms or low-lying n-π* transitions.^47^ Φ_T_(ISC) was somewhat greater for *p*Tol-*m*DPH than for *p*Tol-*p*DPH (∼5% vs ∼1%) but both are of comparable magnitude to the reported ISC efficiency of native DPH in nonpolar solvents (2.9%).^48^

The concentration dependence on Φ_T_ is strong evidence for SF since in solution, SF of monomeric materials is limited by intermolecular interactions; other mechanisms for triplet generation are unimolecular and thus inherently concentration-independent. Crucially, this concentration dependence presents the first evidence for a new class of materials capable of undergoing SF in solution. Previous reports of intermolecular SF in solution have been limited to acene derivatives^49−53^ and aggregated carotenoids.^54−56^

The onset of triplet formation due to intermolecular singlet fission occurs in the region ∼1 to 10 mM. Given a plateau is not reached, at higher concentrations, further increases in Φ_T_ may be possible. Indeed, for TIPS-pentacene, Φ_T_ increases with concentration and plateaus at ∼75 mM.^7^ However, solubility limits inhibited the practical feasibility of exploring higher concentrations in these materials.

Unlike the monomeric materials discussed in this section, dimeric materials have the potential to pre-form a triplet pair state via iSF. Understanding what states might be involved at longer timescales requires an appreciation of the ultrafast intramolecular photophysics, so we will first consider the data from femtosecond resolved transient absorption spectroscopy (fsTA) for the dimers before returning to nsTA.

### Femtosecond Transient Absorption (fsTA)

fsTA was utilized to probe the ultrafast photophysical response on timescales up to 1.8 ns. At dilute concentrations <̃ 1 mM, these timescales are relevant to fast intramolecular processes. Intermolecular processes are highly unlikely to be significant, in consideration of the timescale and concentrations required to observe such effects in the nsTA experiments on the monomers.

**Figure 4:**
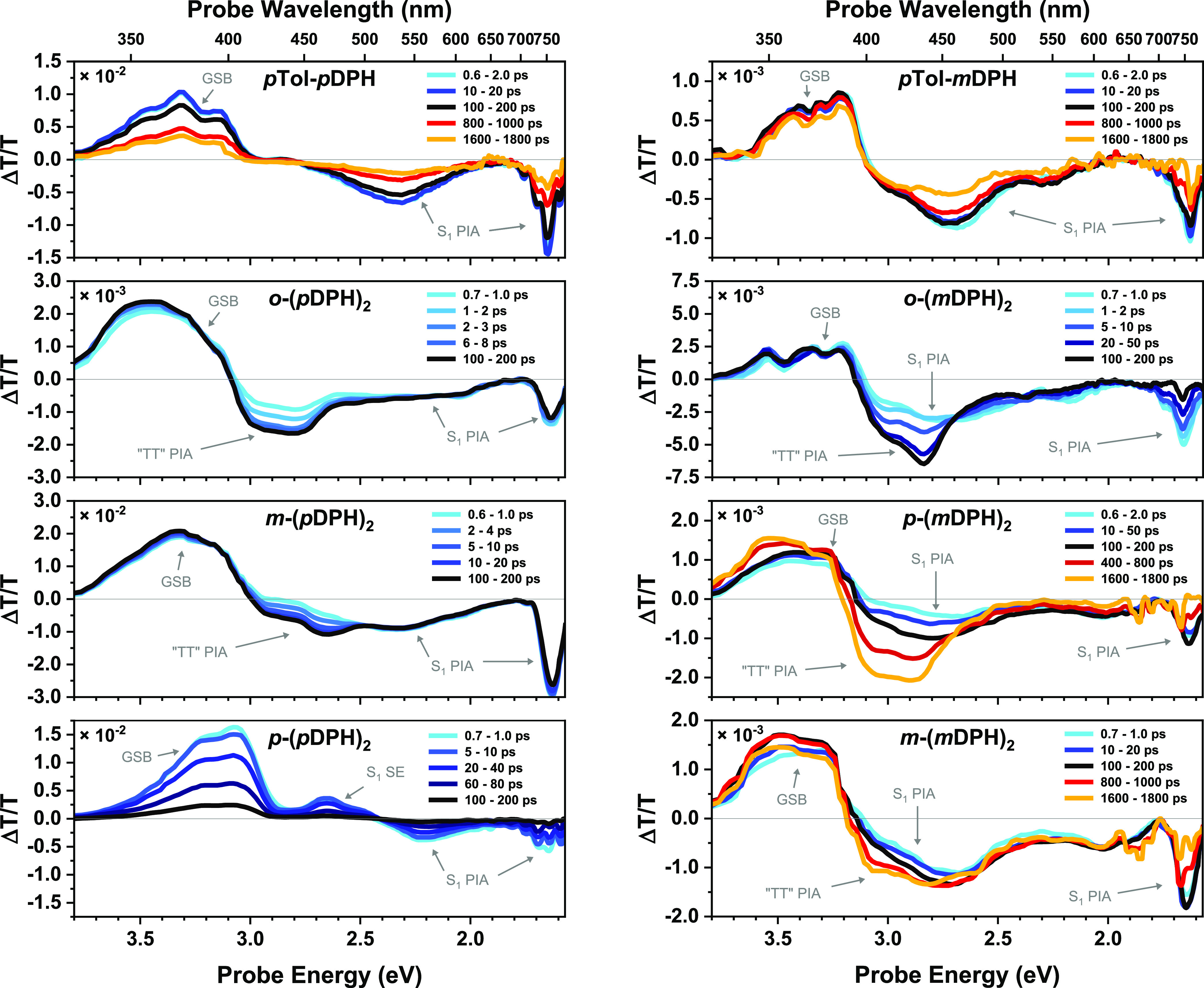
fsTA spectra of dilute solutions (<̃1 mM), with excitation at 400 nm. Time intervals have been selected as appropriate to indicate the decay or evolution of spectral shape for each material. For the materials that display rapid spectral evolution and then decay (*o*-(*p*DPH)_2_, *m*-(*p*DPH)_2_ and *o*-(*m*DPH)_2_), the early time intervals up to the peak are shown here, while later time intervals featuring the decay may be found in the SI (Figure S4).

Figure 4 illustrates the fsTA spectra of the materials at key time intervals. The fsTA spectra of the monomers contain a ground-state bleach (GSB) feature and two PIA features, which all decay concomitantly. Accounting for the different spectral sensitivity of the two instruments, this matches the initial state seen in the nsTA spectra. We assign these spectra to the S_1_ singlet states of the materials. There is no evidence of distinct S_2_ and S_1_ states indicating that this transition results in negligible spectral change or occurs too quickly to be observed on this experimental setup (accounting for a strong artifact that obscures the spectra in the first ∼600 fs). The assignment of the observable state at ≥700 fs to S_1_ is supported by comparison to the S_1_ → S_n_ spectrum and S_2_ → S_1_ transition timescale (610 fs) reported for native DPH by Hirata et al.^57^

While the NIR PIA feature at ∼1.7 eV is similar for both monomers, there is a distinct hypsochromic shift of the higher energy PIA for *p*Tol-*m*DPH, further highlighting that conjugation of the hexatriene with the tolyl group has a moderate influence on the energetics of the excited-state manifolds.

Among the dimers, *p*-(*p*DPH)_2_ is the only material to exhibit no spectral evolution with all features decaying concomitantly. The decay fits to a monoexponential with a comparatively rapid time constant (τ = 73 ps), substantially faster than the decay of the monomer *p*Tol-*p*DPH. *p*-(*p*DPH)_2_ has similar GSB and PIA features to *p*Tol-*p*DPH albeit with the additional presence of a stimulated emission (SE) feature at ∼2.65 eV. The rapid decay is consistent with the low PLQE and likely arises from fast internal conversion to the ground state.

In the remaining dimers, the fsTA clearly indicates multiple distinct electronic states. For *m*-(*p*DPH)_2_ and all three *m*DPH dimers, the early PIA (∼1 ps) strongly resembles the relevant monomer (singlet) spectrum. The dimers then display the emergence of a new PIA feature at ca. 3.2–2.7 eV, which is accompanied by a reduction in the intensity of the NIR singlet PIA feature. The rate of growth of the new feature varies significantly between the dimers. At the extremes, in *o*-(*p*DPH)_2_, this feature is already present at ∼700 fs, while in *m*-(*m*DPH)_2_, it barely appears as a shoulder on the singlet PIA at ∼1800 ps. Moreover, while the intensities of the characteristic singlet features decrease these do not decay completely in all cases.

In *o*-(*p*DPH)_2_ and *m*-(*p*DPH)_2_, the singlet NIR PIA still has significant intensity by the time interval that the emerging PIA (∼3.2 to 2.7 eV) reaches its maximum. The resulting spectra then decay approximately uniformly (SI Figure S4). These observations suggest that the conversion from the singlet state to the state that produces the emergent PIA does not go to completion but reaches a molecule-specific equilibrium followed by decay of the states in tandem.

In *o*-(*m*DPH)_2_, a fine structure in the ca. 3.2–2.7 eV PIA can be discerned that is not so sharply resolved in the other materials. Two narrower peaks can be seen to vary slightly in their relative intensity over time spectrum (SI Figure S4d). The slightly higher energy peak (ca. 3.2–2.95 eV) decreases in intensity such that the feature approaches a single sharp peak (ca. 2.95–2.7 eV), matching well to the sensitized triplet. We hypothesize that this may be direct spectroscopic evidence of two similar but distinct types of state with triplet character and consequently of singlet fission. The sharp triplet signal at later intervals may correspond to a dissociated state “T_1_ + T_1_”, in which two triplets appear independent despite residing upon the same molecule. The bluer shoulder (∼3.2 to 2.95 eV) may arise from the singlet fission intermediate, a correlated ^1^(TT) state. This assignment aligns well with reports of spectral observations of the ^1^(TT) state in a selection of other SF materials^53^ and adds to the growing body of evidence indicating that the ^1^(TT) transient absorption signatures should be similar albeit slightly blue-shifted from the free triplet signatures due to the binding interaction.^58^

Following the assignment of the emergent PIA to TT states, a decay-associated algorithm was employed to model the iSF dynamics. The data were fitted to model kinetic systems of ordinary differential equations with no spectral input (see SI Section 4.iii for details). All systems were fitted using two excited states, nominally S_1_ and “TT”, *i.e.*, not distinguishing ^1^(TT) and “T_1_ + T_1_”. Species-associated spectra (SAS) and comparison of the fit kinetics with the data can be found in the SI (Figures S5 and S6). Table 2 summarizes the triplet parameters inferred from the fitting of both the fsTA and nsTA data.

**Table 2:** Triplet Yields and Time Constants Determined from Transient Absorption Spectroscopy Experiments in Dilute Solutions

compound	Φ_T_ (%)	τ_iSF_	τ_TT_ (τ_1_; τ_2_)	relative amplitudes (A_1_; A_2_)^f^	τ_T_ (μs)^f^
*p*Tol-*p*DPH	0.8 ± 0.4^a^^,^^b^				47 ± 6
*o*-(*p*DPH)_2_	54^c^	7 ± 2 ps^e^	2.5 ± 0.4 ps^e^		
*m*-(*p*DPH)_2_	32^c^	55 ± 9 ps^e^	10.6 ± 0.9 ps^e^		
*p*-(*p*DPH)_2_	0				
*p*Tol-*m*DPH	5.3 ± 1.7^a^^,^^b^				60 ± 2
*o*-(*m*DPH)_2_	163 ± 63^b^^,^^d^	25 ± 3 ps^e^	3.5 ± 0.6 ns^e^^/^f; 25 ± 2 ns	0.74; 0.26	44 ± 5
*m*-(*m*DPH)_2_	48 ± 15^b^	2.2 ± 0.2 ns^f^	44 ± 2 ns^f^		50 ± 2
*p*-(*m*DPH)_2_	119 ± 35^b^	1.1 ± 0.4 ns^e^^/^f	12.3 ± 0.2 ns^f^; 89 ± 4 ns	0.88; 0.12	44 ± 1

a Intersystem crossing yield.

b Determined from nsTA data and the triplet absorption cross section from sensitization.

c Calculated by doubling the “TT” yield determined kinetically from the fsTA data.

d Extrapolated from instrument response limited nsTA data: See the SI for calculation details.

e Determined from fitting of fsTA data.

f Determined from fitting of nsTA data.

For *o*-(*p*DPH)_2_ and *m*-(*p*DPH)_2_, the reverse TT → S_1_ rate (1/2.5 and 1/10.6 ps^–1^, respectively) is noted to be faster than the forward process (1/7 and 1/55 ps^–1^) such that an equilibrium maintaining significant singlet population is established. The position of these equilibria toward the singlet fundamentally limits the SF yields of these materials.

Perhaps the most promising iSF candidate, *o*-(*m*DPH)_2_ displays a rise of the “TT” feature fitted by a fast time constant of 25 ± 3 ps. Significantly, the NIR singlet signal decays completely in this timeframe and a model involving the reverse rate could not be fit. This indicates that the “equilibrium” can be considered to reach full conversion i.e., about unity SF efficiency. The slight shifting of intensity between the two peaks of the TT PIA band was not treated in this model.

The remaining *m*DPH dimers displayed very slow transitions with only partial triplet pair formation in the final time intervals of the fsTA experiment. A time constant for the S_1_ → TT transition of 900 ± 200 ps was determined for *p*-(*m*DPH)_2_, but this likely represents a lower bound since the spectrum has not finished evolving by the longest delay interval. The transition for *m*-(*m*DPH)_2_ is slower again, with the characteristic TT PIA only just beginning to emerge as a shoulder from the singlet PIA.

### Nanosecond Transient Absorption(nsTA): Dimers

Further investigation of the triplet pair dynamics and free triplet formation in the dimers was carried out using nsTA, to explore the fate of the TT states in these materials and investigate the origin of the delayed fluorescence in the *m*DPH family.

**Figure 5:**
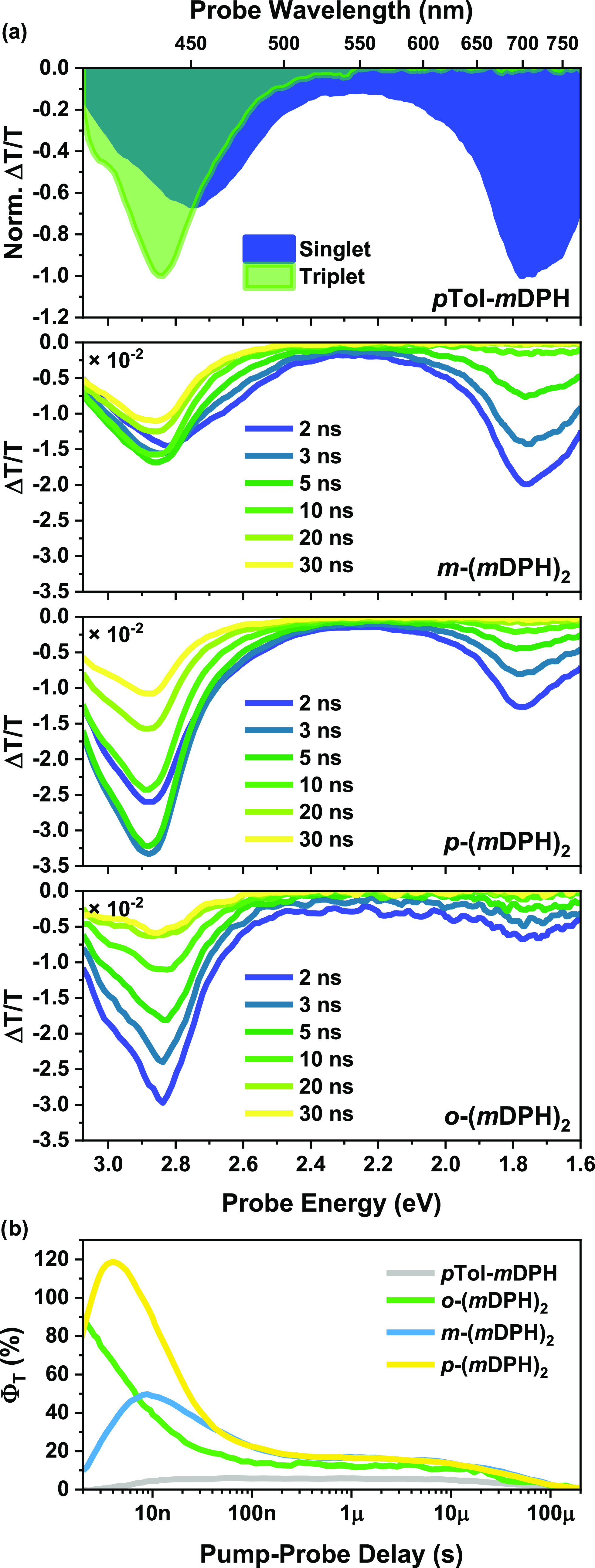
(a) nsTA spectra up to 30 ns for dilute solutions (<̃1 mM) of the *m*DPH dimers. The normalized singlet (1 mM, 2 ns data) and triplet (sensitization data) for the monomer are indicated for reference in the top panel. (b) Dilute nsTA triplet kinetics extracted using a global analysis genetic algorithm and normalized by the calculated triplet yields.

The spectra of *o*-(*p*DPH)_2_ and *m*-(*p*DPH)_2_ continue the decay observed in the fsTA experiment without significant evolution in spectral shape (SI Figure S7). The kinetics closely match the fluorescence lifetimes (SI Figure S7b, Table 1), supporting assignment to the state mixture S_1_ ⇌ ^1^(TT). The spectra of all three *m*DPH dimers exhibited similar form (Figure 5a). A triplet PIA (ca. 2.6–3.1 eV) overlaps with and emerges out of the singlet PIA (ca. 3.1–2.4 eV and <̃2 eV). The intensity of the peak of the singlet NIR PIA at the instrument response limited early timepoint (∼2 ns) increases in the order: *o*-(*m*DPH)_2_ < *p*-(*m*DPH)_2_ < *m-*(*m*DPH)_2_. The increasing proportion of the singlet at the early time intervals of the nsTA experiment agrees with the trend in decreasing SF rates observed in the fsTA data. In addition, for *m-*(*m*DPH)_2_ and *p*-(*m*DPH)_2_, the peak triplet signal occurs at a timepoint slower than the instrument response. By contrast, for *o*-(*m*DPH)_2_, all parts of the spectrum peak at ∼2 ns, clearly suggesting that the peak position is instrument response limited. This matches expectations considering that the signal peaks at ∼100 ps in the fsTA experiment.

Due to overlapping singlet and triplet PIA features, Φ_T_ determination required deconvolution of the nsTA spectra. Deconvolution was undertaken using a global analysis genetic algorithm (SI, Figures S8 and S9). A curve of Δ*T*/*T* vs pump-probe delay was determined for the triplet state for each of the *m*DPH materials. Using the triplet absorption cross section calculated from sensitization data, the Δ*T*/*T* values could be converted to Φ_T_ values (Figure 5b, Table 2).

The *m*DPH dimers’ triplet features decay in two distinct phases: the initial decay from the peak triplet population (<̃300 ns) and then the remaining triplet population that decays monoexponentially over tens of microseconds. The slow decay is of the same order of magnitude as the triplet lifetime of the monomer and the triplet lifetimes observed during the sensitization experiments, which generate a single triplet on the sensitized molecule. Accordingly, we attribute this microsecond lifetime to triplet states on molecules where there is only one triplet species present. We assign the sub-microsecond decay components to processes involving molecules hosting two triplets, shown in Table 2 as “τ_TT_”. Exponential fitting found a decay time constant of 44 ± 2 ns for *m*-(*m*DPH)_2_, while two exponential components were required to give reasonable fitting to the sub-microsecond decay for the other *m*DPH dimers.

In sharp contrast to the *p*DPH materials for which the fluorescence lifetimes (Table 1) and nsTA decay lifetimes (SI Figure S7b) are a close match, there is not a clear correlation for the *m*DPH dimers. Where the *p*DPH dimers form a triplet pair that is strongly bound in an equilibrium favoring the singlet state, the triplet pair in the *m*DPH materials is able to undergo spin evolution, decoupling the PL and TA kinetics; while TA tracks the decay of the triplet feature (“TT”/T_1_), emission is only possible while the triplet pair remains spin-correlated into an overall singlet, ^1^(TT).

The slow iSF activity in *m*-(*m*DPH)_2_ corresponded to the lowest peak Φ_T_ among the *m*DPH dimers (48 ± 15%). Exponential fitting to the growth of the curve in Figure 5b results in a fit time constant for fission of 2.2 ± 0.2 ns. Notably, even the “slow” iSF in *m*-(*m*DPH)_2_ results in a much greater Φ_T_ than expected from ISC, as indicated by the Φ_T_ of *p*Tol-*m*DPH in dilute solution (∼5%).

*p*-(*m*DPH)_2_ demonstrated a moderate peak Φ_T_ of 119 ± 35%. The fitted time constant for the rise in triplet signal was 1.4 ± 0.1 ns, although being of similar magnitude to the instrument response, this may be considered as an upper bound. In conjunction with the lower bound taken from the fsTA data, a fission time constant of 1.1 ± 0.4 ns may be suggested.

As discussed earlier, the peak for *o*-(*m*DPH)_2_ was instrument-response-limited, with significant decay from the true maximum occurring within the instrument response time of the nsTA experiment. A value of Φ_T_ = 90 ± 27% was calculated that corresponds to the instrument response limited peak and represents a lower bound for the Φ_T_. Utilizing the determined decay constant, this value was extrapolated back to the peak at ∼100 ps, to obtain a calculated peak triplet yield of 163 ± 63% (calculation details in SI Section 5). While the error in this value is large, in conjunction with the fast iSF kinetics, it is evident that *o*-(*m*DPH)_2_ is capable of high-yielding intramolecular singlet fission.

Next, we consider the long-lived triplet population at the kinetic “plateau” occurring at timescales between intramolecular and unimolecular triplet decay. In the dilute regime, the long-lived triplet yield of the dimers may arise from ISC (as in the monomer), but spin evolution of the triplet pair presents an additional mechanism. Once no longer coupled into an overall singlet, recombination of the triplet pair can generate a singular higher-lying triplet excited state, which will decay to T_1_ by internal conversion:
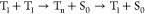
The long-lived Φ_T_ for dilute solutions of the dimers is ∼15 ± 5%, appreciably greater than the ISC Φ_T_ of the monomer (Figure 5b.). This may be considered as evidence for contribution from a triplet-pair-mediated mechanism. Ultimately, this long-lived Φ_T_ represents nonmultiplicative triplet generation (one triplet generated from one photon absorbed) and can be considered as parasitic to effective exciton multiplication by SF.

The most soluble dimer, *m*-(*m*DPH)_2_, was studied across a range of concentrations enabling comparison with the monomer. While the peak (iSF) Φ_T_ appears concentration-independent, the long-lived Φ_T_ and triplet decay kinetics do exhibit concentration dependence (SI Figure S10). At 10 mM, the initial triplet decay lifetime drops to 33 ± 2 ns from the 44 ± 2 ns of the dilute case and the plateau occurs at a greater fraction of the peak triplet population. This suggests the involvement of an intermolecular contribution to triplet formation in *m*-(*m*DPH)_2_ at sufficiently high concentration. There are two distinct explanations for the intermolecular contribution to triplet formation. Like a monomer, the dimer may undergo intermolecular SF upon molecular collisions in solution. Alternatively, long-lived free triplets could be generated following iSF, through intermolecular triplet energy transfer of one of the triplets from a dimer with two triplets (“T_1_ + T_1_”) to a second molecule in its ground state, as has been demonstrated for of dicyano-oligoene materials in the solid state.^59^

## Discussion

The photophysical behavior of the DPH moiety is significantly influenced by structural differences between derivatives. Conjugatively linking a tolyl group in *p*Tol-*p*DPH versus breaking the conjugation with the hexatriene in *p*Tol-*m*DPH manifests as an increased radiative rate, which in turn influences the yields of ISC and SF. We attribute this change to the stabilization of the bright state enabled by greater delocalization in the *para*-substituted geometry.

When designing derivatives of DPH, the varying sensitivities of the singlet and triplet states must be considered. This has relevance to the design of optimized iSF dimers, where dependent upon the geometry, the linker may modulate the inherent properties of the chromophore to a greater or lesser extent. Ultimately, the phenylene linker in our *p*DPH family is much less innocent than the same linker in their *m*DPH analogues. In the *m*DPH derivatives, the linker provides a convenient handle by which to tune the relative geometries of the two DPH units and the degree of interaction, in turn controlling singlet fission activity. However, in the *p*DPH family, the direct conjugation of the phenylene with the hexatriene units fundamentally alters the photophysics of the chromophore. In the most extreme case, this manifests as the polyene-like rapid deactivation by internal conversion in *p*-(*p*DPH)_2_. This presents an informative contrast to the analogous *p*-phenylene TIPS-pentacene dimer, referred to as “BP1” by its authors, which exhibits iSF activity with τ_SF_ = 20 ps and an intramolecular triplet decay timescale of 16.5 ns.^34^ In TIPS-pentacene derivatives, such as BP1, the excited states are known to be stabilized and partially localized away from the linker toward the TIPS groups,^34^ while in DPH the excited states are delocalized across the chromophore.^9^ Consequently, equivalently linked DPH dimers should be expected to demonstrate stronger intramolecular coupling than analogous TIPS-acene dimers, which matches our experimental findings.

In the other two *p*DPH dimers, *o*-(*p*DPH)_2_ and *m*-(*p*DPH)_2_, iSF activity is observed, but we observe a tightly bound triplet pair, which establishes equilibrium with the singlet in a matter of picoseconds. Evidently, to achieve triplet decorrelation, disabling direct through-bond electronic coupling is of critical importance.

Having disabled conjugative interaction of the hexatriene units with the phenylene linker in the *m*DPH family, these dimers display tunable iSF activity with capacity for spin dephasing of the triplet pair. At ∼1 ps following excitation, all three dimers display singlet characteristics matching those of the monomer. All *m*DPH dimers undergo iSF, on the nanosecond timescale for *m*-(*m*DPH)_2_ and *p*-(*m*DPH)_2_ but 2 orders of magnitude faster in *o*-(*m*DPH)_2_. It is clear that a much closer spatial proximity of the DPH units is possible in *o*-(*m*DPH)_2_ than its isomers (Figure 1). Therefore, in hexatriene-based iSF systems, while through-bond communication is best avoided, the molecular geometry should be optimized for close spatial proximity of the chromophore units.

The triplet decay of the *m*DPH dimers exhibited multiple components. The trend in the rate of the fastest, dominant decay pathway paralleled the singlet fission rate, as is typical of iSF dimers. The same factors which govern the chromophore interaction driving SF also determine the rate of the reverse process, which equally strongly depends on the interaction of the two chromophores. The propensity for rapid (<100 ns) loss of the additional triplet state, generated by fission, in confined molecular systems represents a limitation to their practical utility. In consideration of this, the community has begun to establish strategies for reducing the intramolecular triplet recombination rate in acene systems, through the provision of entropic and energetic driving forces for triplet separation within a single molecule.^32,60−62^ Incorporating the dimer design principles found in this work into larger oligomeric structures presents an avenue for future work toward enhancing the lifetime of iSF-generated triplets in DPH systems. Alternatively, the long lifetime of molecularly separated triplet states could be leveraged via a hybrid intra–intermolecular singlet fission mechanism taking place in concentrated rather than dilute solution. This would require the design of a highly soluble derivative capable of undergoing fast iSF and then intermolecular triplet transfer before intramolecular triplet decay can occur.

## Conclusions

A series of novel dimeric structures and monomeric reference compounds based upon the high-triplet-energy chromophore diphenylhexatriene, DPH, have been synthesized and evaluated for potential singlet fission activity using transient absorption spectroscopy and time-correlated single photon counting. The best-performing material, *o*-(*m*DPH)_2_, displays fast iSF activity (τ_iSF_ = 25 ± 3 ps) with a triplet yield of 163 ± 63%. As such, DPH dimers present a new class of iSF materials capable of achieving high triplet yields and with triplet energy sufficient for relevance to silicon photovoltaics.

The geometry of covalently linked DPH dimers critically impacts their photophysical properties. Direct conjugation of the hexatriene with the linker unit significantly alters the singlet state dynamics of the *p*DPH materials and favors the singlet in equilibria, where iSF is possible. This contrasts the iSF behavior of analogous phenylene-linked acenes, suggesting significantly stronger coupling in DPH materials as opposed to analogous acene derivatives. However, when *meta* substitution patterns are employed, preventing linear conjugation of the hexatriene with the linker, SF activity is observed to generate decorrelated triplet states. Moreover, the SF rate demonstrates an exceptionally high degree of tunability, with τ_iSF_ tunable by 2 orders of magnitude between the *m*DPH dimers.

The results presented indicate that iSF is optimized by tuning the coupling geometry to maximize the potential for through space interaction, while limiting through-bond communication via direct conjugation. This principle should be applied to further DPH oligomers and will likely have relevance as a design rule for other SF materials.

Finally, concentration-dependent triplet yields provide the first known evidence for intermolecular SF in solutions of DPH derivatives. This presents an entirely new avenue of SF materials to explore, with the potential for more soluble derivatives to enable efficient intermolecular SF in solution with high triplet energy.

## Supporting Information Available

Experimental details including synthetic procedures, additional spectroscopic results, triplet yield calculation methodology, and NMR spectra (PDF)
