# Supplementary material for: Soluble Diphenylhexatriene Dimers for Intramolecular Singlet Fission with High Triplet Energy
Source: J Am Chem Soc. 2023 Jan 23;145(4):2499–510. doi: 10.1021/jacs.2c12060 (PMC9896565; doi:10.1021/jacs.2c12060)
Supplement: Supplementary file 1 — ja2c12060_si_001.pdf [file ja2c12060_si_001.pdf]

Supporting Information

Soluble diphenylhexatriene dimers for intramolecular singlet fission with high triplet energy

Oliver Millington†‡, Stephanie Montanaro†, Anastasia Leventis†, Ashish Sharma‡, Simon A. Dowland‡, Nipun Sawhney‡, Kealan J. Fallon†‡, Weixuan Zeng†, Daniel G. Congrave†, Andrew J. Musser§, Akshay Rao*‡, Hugo Bronstein*†‡

† Department of Chemistry, University of Cambridge, Cambridge, CB2 1EW, UK.

‡ Cavendish Laboratory, University of Cambridge, Cambridge, CB3 0HE, UK.

§ Department of Chemistry and Chemical Biology, Cornell University, Baker Laboratory, Ithaca, NY, 14853, USA.

Table of Contents

1. EXPERIMENTAL DETAILS **2**

2. A NOTE ON PHOTOSTABILITY **6**

3. UV-VIS TEMPERATURE DEPENDENCE STUDY FOR *P*-(*P*DPH)$_2$ **6**

4. FURTHER TRANSIENT ABSORPTION SPECTROSCOPY **7**

i) nsTA: pTol-mDPH contour plots - Concentrated vs Dilute *7*

ii) fsTA: Decay of spectra for o-(pDPH)$_2$, m-(pDPH)$_2$ and o-(mDPH)$_2$ *8*

iii) fsTA: Decay associated deconvolution/ fitting *9*

iv) nsTA: Spectra of dimers o-(pDPH)$_2$ and m-(pDPH)$_2$ *11*

v) nsTA: mDPH materials global analysis fitting *12*

vi) nsTA: m-(mDPH)$_2$ Concentration Series *14*

5. CALCULATION OF TRIPLET QUANTUM YIELDS **15**

Back-extrapolation of Φ_T from the instrument response limited value for o-(mDPH)$_2$ *19*

6. SYNTHETIC INFORMATION **20**

Intermediates *20*

Final Compounds *29*

7. NMR SPECTRA **37**

8. REFERENCES **57**

1. Experimental Details

General Synthesis and Characterisation Details

Glassware was routinely dried in an oven set to 200 °C before use. Flash column chromatography purifications were performed on an autocolumn (Biotage® Isolera) using pre-packed silica gel cartridges purchased from Biotage®. Column conditions were obtained by analytical thin layer chromatography (TLC) using precoated glass backed silica gel plates (Merck Kieselgel 60 F254 0.20 mm). Visualisation was achieved throughout using UV light (254, 365 nm).

Nuclear magnetic resonance spectroscopy was performed on a Bruker 400 MHz Avance III HD Spectrometer, a Bruker 500 MHz Avance III HD Spectrometer or a Bruker 700 MHz TXO Cryoprobe Spectrometer. All spectra taken on these machines were recorded at ambient temperature in the stated solvent with residual protic solvent used as the internal standard. Variable temperature NMR on *p*-(*p*DPH)$_2$ was performed on a 500 MHz DCH Cryoprobe Spectrometer by specialist NMR technician Andrew Mason. Spectra were processed using Mnova (Mestrelab). ^{1}H NMR data is reported as: chemical shift (multiplicity, coupling constant, integration). ^{13}C NMR data is reported as: chemical shift. Chemical shifts (δ) are quoted in parts per million (ppm) relative to residual solvent ($CDCl_3$: δ(^{1}H) = 7.26 ppm, δ(^{13}C) = 77.16 ppm); $CDCl_2CDCl_2$: δ(^{1}H) = 6.01 ppm). Standard abbreviations are used to indicate multiplicities and peak forms: s = singlet, d = doublet, t = triplet, q = quartet, p = pentet, sx = sextet, sp = septet, m = multiplet, br = broad and associated combinations thereof. Coupling constants (J) are quoted to the nearest 0.1 Hz and are assumed to be $J_{H\text{-}H}$ unless otherwise stated.

Mass spectra were obtained using a Waters LCT, Finnigan MAT 900XP or Waters MALDI micro MX spectrometer by the mass spec team at the Department of Chemistry, University of Cambridge.

Chemicals

All commercial chemicals were of ≥95% purity and were used as received without further purification. Anhydrous solvents were purchased from Sigma Aldrich or Acros Organics and used as received for synthesis. Once synthesized, final DPH materials were stored under argon at ~4 °C or in a nitrogen glovebox at ambient temperature.

Transient Absorption

General: A pump and probe beam (details for each setup given below) were overlapped upon the sample cuvette. Coarse alignment was performed with a beam profiler (Thorlabs BC106N-VIS) in the place of the sample followed by optimization of the signal through fine tuning of the sample stage position, to account for discrepancy in the position of the CCD of the profiler when mounted in the sample holder and the position of the cuvette. The pump profile and effective diameter were recorded using the same beam profiler. The transmitted pulses of the probe beam were collected while the pump

beam was blocked after the sample. The power of the pump beam was measured before the sample, after the sample and of the sample back reflection using a Thorlabs PM100D power meter.

Sample Preparation: Exclusion of oxygen was of paramount importance for valid investigation of singlet fission in DPH materials; It is known that oxygen can enhance intersystem crossing as a competitive (non-multiplicative) triplet generating process in DPH.[1,2] Commercial (Sigma-Aldrich) anhydrous toluene (~40-50 ml) was degassed thoroughly by 4-5 cycles of freeze pump-thawing in a 100 ml J. Youngs storage flask and backfilled with argon. This was transferred into a nitrogen glovebox, maintained at <0.1 ppm O_2. Storage of the solvent in the glovebox for up to 6 weeks, even after opening, did not lead to any evidence of oxygen ingress in our TA results. Solution samples were prepared and temporarily stored (for the duration of a measurement batch) in the same glovebox. Samples for TA were transferred to custom 1mm quartz cuvettes, utilized for the measurements. Quartz cuvettes with a graded seal to borosilicate glass (BOROFLOAT®) were purchased from Starna Scientific and J Youngs "NMR valves" attached by the in-house glassblowing service at the Department of Chemistry, University of Cambridge.

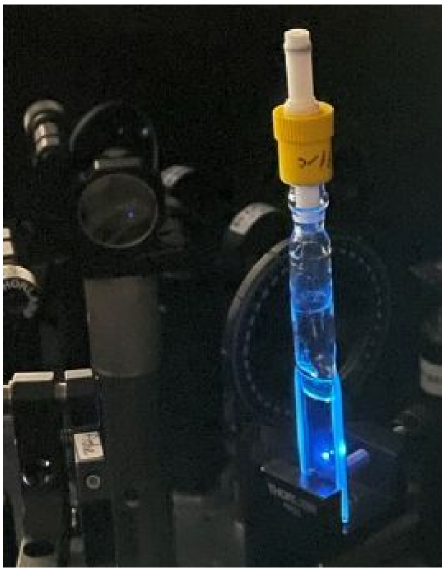

Figure S1: Cuvette fitted with J Young's valve used to exclude oxygen during transient absorption measurements.

Femtosecond transient absorption: The output of a titanium:sapphire amplifier system (Spectra Physics Solstice Ace) operating at 1 kHz and generating ~100 fs pulses was split into the pump and probe beam paths. The 400 nm pump pulses were created by sending the 800 nm fundamental beam of the Solstice Ace through a SHG BBO crystal of 1 mm thickness (Eksma Optics). The pump was blocked by a chopper wheel rotating at 500 Hz. The ultraviolet-visible broadband beam (330–800 nm) was generated by focusing the 800 nm fundamental beam onto a moving CaF_2 crystal (Eksma Optics, 5 mm) connected to a digital motion controller (Mercury C-863 DC Motor Controller) after passing through a mechanical delay stage (Thorlabs DDS300-E/M). The transmitted pulses were collected with a monochrome line scan camera (JAI SW-4000M-PMCL; spectrograph, Andor Shamrock SR-163) with collected data fed straight into the computer.

The setup used for the fsTA experiment is optimized for observation of the ultraviolet and blue spectral regions. This comes at the cost of reduced sensitivity and probe stability in the deep-red and near infra-red (NIR), resulting in apparent distortions in this region. This is most obtrusive in the late intervals for the *m*DPH family but nevertheless the underlying dynamics can be discerned.

Nanosecond transient absorption:

400 nm pump (used for measurements on *p*Tol-*p*DPH): The output of a titanium:sapphire amplifier system (Spectra Physics Solstice Ace) operating at 1 kHz and generating ~100 fs pulses was used. The 400 nm pump pulses were created by sending the 800 nm fundamental beam of the Solstice Ace through a second harmonic generating (SHG) beta barium borate (BBO) crystal of 1 mm thickness (Eksma Optics). The pump was blocked by a chopper wheel rotating at 500 Hz.

355 nm pump (All other materials): The pump was generated by the third harmonic (355 nm) of a Q-switched Nd:YVO_4 (1 ns pump length, Advanced Optical Technologies Ltd AOT-YVO-25QSPX). The pump was blocked by a chopper wheel rotating at 500 Hz.

532 nm pump (Sensitization with PdOEP): The second harmonic (532 nm) of the same laser utilized for the 355 nm was pump was used.

Probe (All nsTA measurements): The probe beam was generated with a LEUKOS Disco 1 UV supercontinuum laser (STM-1-UV, 1 kHz). The probe was split by a 50% beam splitter into a reference and probe and both were focused onto the sample. The reference beam passing through the sample did not interact with the pump, which allows for correcting for any shot-to-shot fluctuations in the probe that would otherwise greatly increase the structured noise in the experiments. A pair of line image sensors (Hamamatsu, G11608) mounted on a spectrograph (Andor Solis, Shamrock SR-303i) were used to detect the signal, using a custom-built board from Entwickslungsbüro Stresing to read out the signal. The delay was controlled electronically for all nsTA measurements.

Steady-State Photophysics

Steady state absorption and photoluminescence measurements on dilute toluene solutions were carried out in 10 mm fluorescence cuvettes with a screw cap featuring a seal (Starna Scientific), providing moderate air exclusion for the short time periods required for these experiments.

Absorption of all samples was measured using a Shimadzu UV-3600 Plus spectrophotometer.

The steady-state photoluminescence was measured using an Edinburgh Instruments FLS980 Spectrometer, comprising a 450 W Xe1 xenon arc lamp focused into a monochromator to excite the samples at 355 or 385 nm and an R928P PMT to detect emission between 375 – 650 nm.

PLQE

PLQE measurements on toluene solutions were carried out in 1 mm glass cuvettes sealed under nitrogen with PTFE screwcaps; the custom cuvettes detailed above were too large to be used within an integrating sphere. PLQE experiments were run promptly following sample preparation to minimise the possibility of PLQE distortion arising from oxygen ingress. Measurements were made following the procedure of de Mello *et al.*[3] Temperature and current controlled laser diodes (Thorlabs) were used to generate stable laser beams. These were focused through a small hole onto samples suspended in a Spectralon coated integrating sphere (Newport 819C-SL-5.3) modified with a custom baffle extension. Light from the experiment was collected using an optical fibre connected to a Andor Kymera 328i Spectrometer housing a DU420A Silicon CCD detector. This setup was calibrated using a using a Bentham 610 QTH calibration source. Calibration was verified through measurement of the fluorescence standard Rhodamine 6G (100 µM in EtOH), for which a PLQE of 92 % was measured, agreeing well with literature values.[4] As best practise, the PLQE values of all of our samples were measured 4-fold and a mean taken (reported in Table 1); the standard deviations of the measurement set for each sample were all within 1 %.

TCSPC

The same samples/ cuvettes used for TA were utilized for TCSPC measurements. Samples were excited with a pulsed 375 nm diode laser (PicoQuant LDH-P-C-375-B operated at 10 MHz). Scattered laser light was removed from the photoluminescence by two long pass filters (LP385 nm and LP400 nm) inserted into the path of the collection optics. The laser was controlled by a trigger box/power supply unit (PDL 800-B, PicoQuant). TCSPC utilised an emission spectrometer (Lifespec-ps unit, VTC900 PCI card, Edinburgh Instruments) with a multi-channel plate detector (R3809U-50, Hamamatsu). The instrument response was determined by scattering excitation light into the detector using a piece of scratched glass; a value of 137 ps was obtained. Fluorescence lifetimes were obtained by exponential fitting of the data using OriginLab software, over an appropriate range (> 1 ns) well beyond the timeframe of the instrument response.

2. A Note on Photostability

Photoinduced isomerization has been a well-studied phenomenon in native DPH.[5–7] On the basis of these existing studies we expect photoisomerization may occur to some extent within our materials family. In the long-term, stability demonstration may be expected to become increasingly important for singlet fission materials that aim to be device viable. Our aim in this work has been to begin to establish design principles for iSF systems based on DPH, that can be applied toward future materials design. None of the materials in this work are claimed to be a device ready singlet fission molecule and detailed stability studies are considered outsight the scope of this work. In this regard, DPH derivatives are not so different to the acenes that have dominated SF research despite their well-known ability to undergo photodimerization reactions.[8] Finally, if future studies reveal isomerization to be a potential pitfall for DPH based SF systems, one can synthetically envisage several ways to fuse parts of the hexatriene backbone in order to reduce isomerization. Indeed, there is some existing literature to this end.[9]

3. UV-Vis temperature dependence study for *p*-(*p*DPH)$_2$

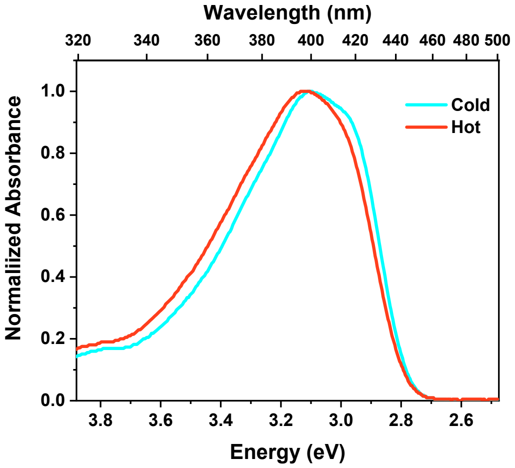

Figure S2: UV-Vis absorption spectra of *p*-(*p*DPH)2 taken in 1,2-dichlorobenzene. The solution was prepared and passed through a syringe filter before taking the cold measurement. The sample was then heated until boiling and the hot measurement taken immediately. There is no blue shift at the onset although a small shift along the curve of the peak but much smaller than the ~0.15 eV difference between *p*-(*p*DPH)$_2$ and the other *p*DPH materials.

4. Further Transient Absorption Spectroscopy

i) nsTA: *p*Tol-*m*DPH contour plots - Concentrated vs Dilute

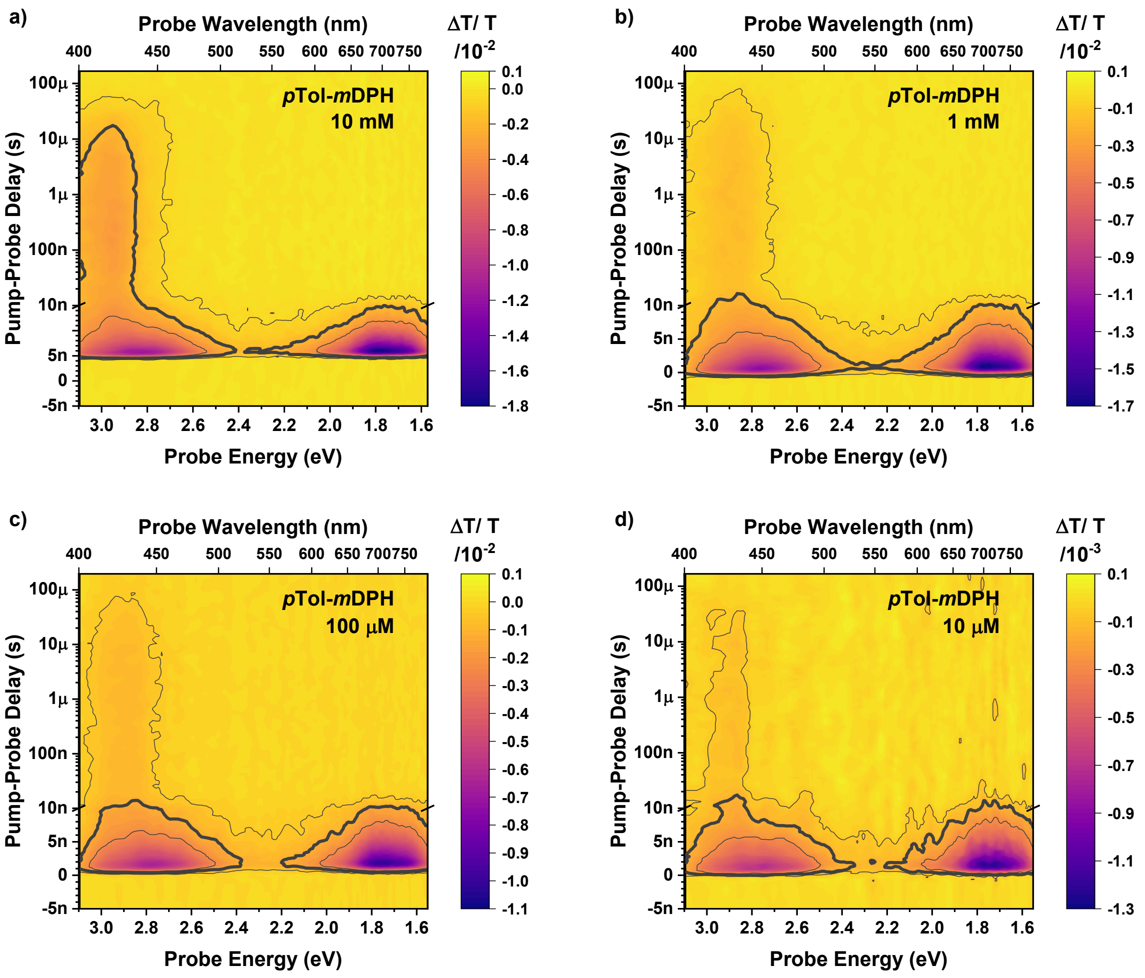

Figure S3. nsTA (exc. 355 nm) contour plots for *p*Tol-*m*DPH at concentrations spanning four orders of magnitude. Contour lines are indicated at ~22 %, 11 % (bold) and ~ 4 % of the maximum intensity. In a) the contour at ~ 11% highlights long lived triplet signal at this threshold while in b)-d) long lived signal is only observed at the lower threshold of ~ 4 % of the maximum PIA intensity. This may be interpreted as indicating only low yielding triplets formed by ISC in the three lower concentration regimes, while at the highest concentration the triplet signal is enhanced by the onset of intermolecular singlet fission.

ii) fsTA: Decay of spectra for *o*-(*p*DPH)$_2$, *m*-(*p*DPH)$_2$ and *o*-(*m*DPH)$_2$

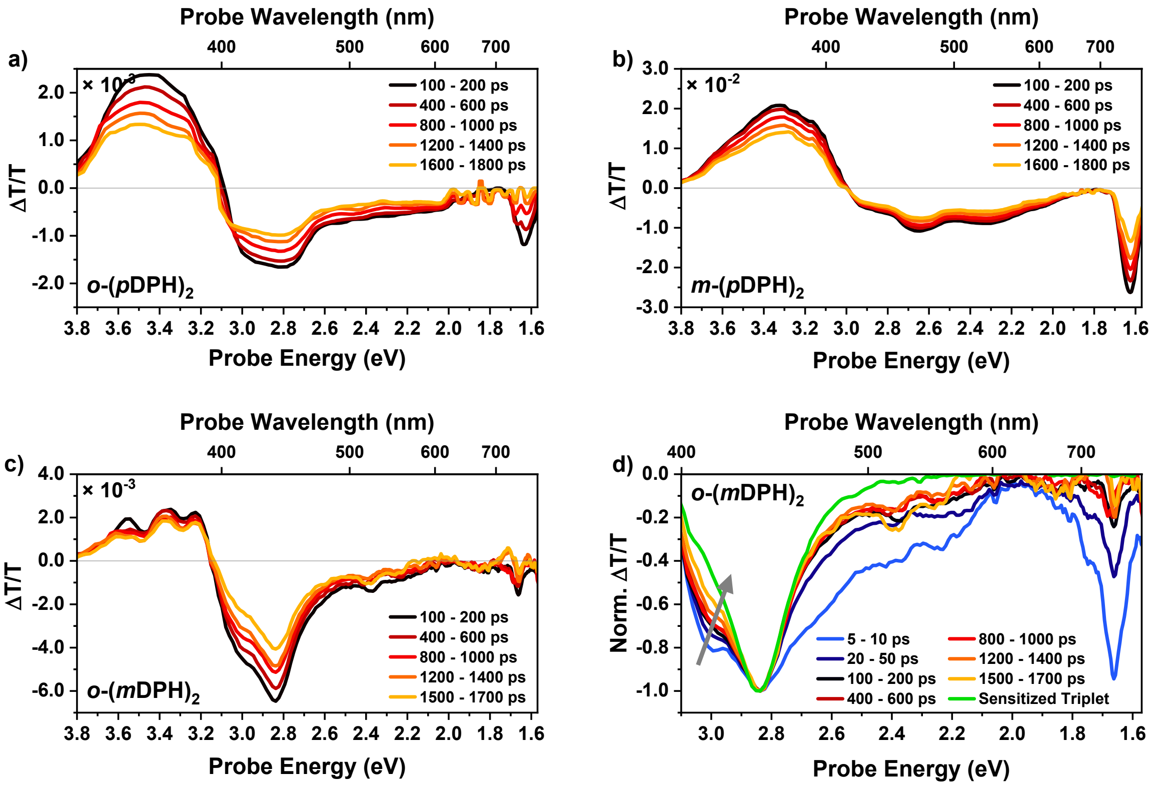

Figure S4. a)-c) Later time intervals of the fsTA spectra of select dimers, complementing the data shown in Figure 4 for earlier intervals. d) Normalized TA spectra for *o*-(*m*DPH)$_2$ over the later time intervals of the fsTA experiment with comparison to the sensitized triplet spectrum.

iii) fsTA: Decay associated deconvolution/ fitting

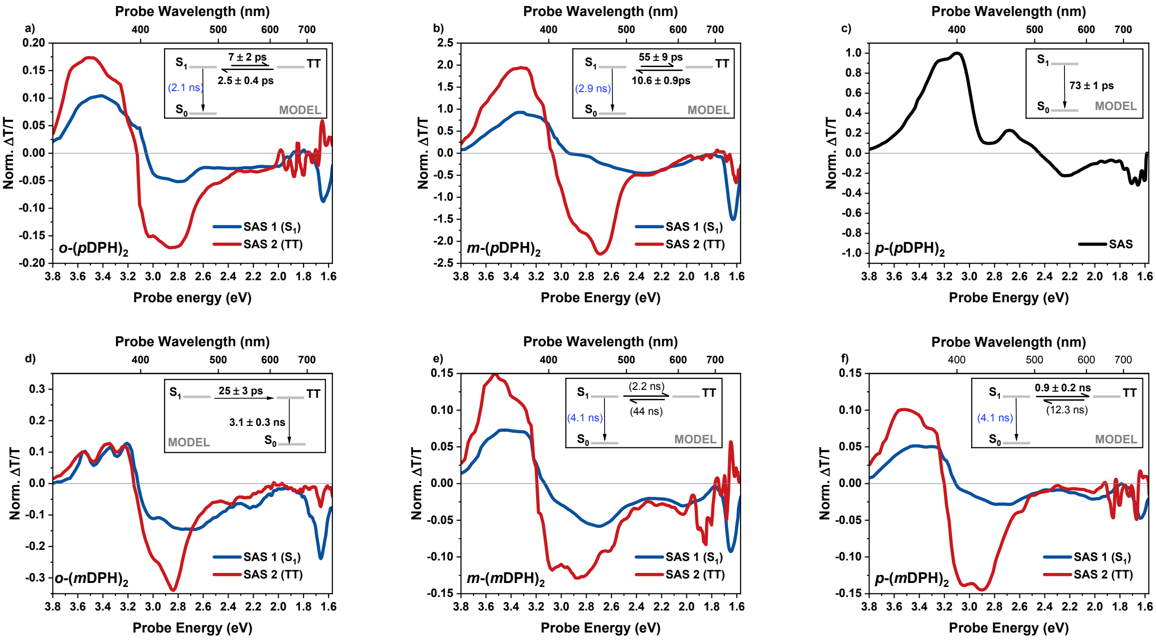

Figure S5. Species associated spectra (SAS) generated by decay associated (DAS) fitting of the fsTA data of the dimers. Fitting was performed through DAS analysis, whereby systems of ordinary differential equations (ODE's) were set up with up to two unknown rate/time constants (applying a kinetic model) and solved to give the best fit to the data. Model schemes are inset (and written in full on the next page), with time constants that were optimized indicated in bold. In some models other (fixed) time constants were included, taken from either TCSPC or fitting of nsTA data. These are indicated in parentheses and non-bold font, in blue and black fonts respectively. Approaching timescales on the order of nanoseconds required that some consideration of fluorescence from the singlet was built into the models for *m*-(*m*DPH)$_2$ and *p*-(*m*DPH)$_2$. This was done by including the fluorescence lifetime (~4.1 ns) of the monomer, *p*Tol-*m*DPH, as a base decay rate. Alone amongst the materials, due to the slow rates involved, the model parameters for *m*-(*m*DPH)$_2$ were entirely fixed by data from the TCSPC and nsTA experiments and the model was not optimized during the fit to the fsTA data.

SAS 1 typically demonstrates a good qualitative match to the shape of the relevant monomer singlet spectrum. SAS 2 shows a strong similarity in all of the SF active materials that is more difficult to observe in the raw data, where the mixing with S_1 features varies between the materials. This indicates that the arguments used to assign the emergent PIA to "TT" for *o*-(*m*DPH)$_2$ may be extended to the remaining dimers.

Ordinary Differential Equation Systems:

o-(*p*DPH)$_2$: dy(1) = (1/tau(2))*y(2)-(1/tau(1))*y(1)-(1/2100)*y(1);

dy(2)= (1/tau(1))*y(1)-(1/tau(2))*y(2);

Solution: tau(1) = 7 ± 2 ps, tau(2) = 2.5 ± 0.4 ps

m-(*p*DPH)$_2$: dy(1) = (1/tau(2))*y(2)-(1/tau(1))*y(1)-(1/2900)*y(1);

dy(2)= (1/tau(1))*y(1)-(1/tau(2))*y(2) ;

Solution: tau(1) = 55 ± 9 ps, tau(2) = 10.6 ± 0.9 ps

p-(*p*DPH)$_2$: dy(1) = -(1/tau(1))*y(1) ;

Solution: tau(1) = 73 ± 1 ps

o-(*m*DPH)$_2$: dy(1) = -(1/tau(1))*y(1);

dy(2)= (1/tau(1))*y(1)-(1/tau(2))*y(2) ;

Solution: tau(1) = 25 ± 3 ps, tau(2) = 3100 ± 300 ps

m-(*m*DPH)$_2$: dy(1) = -(1/2200)*y(1)-(1/4100)*y(1))+(1/44000)*y(2);

dy(2)= (1/2200)*y(1)-(1/44000)*y(2);

p-(*m*DPH)$_2$: dy(1) = -(1/ tau(1))*y(1)-(1/4100)*y(1))+(1/12300)*y(2);

dy(2)= (1/ tau(1))*y(1)-(1/12300)*y(2) ;

Solution: tau(1) = 900 ± 200 ps

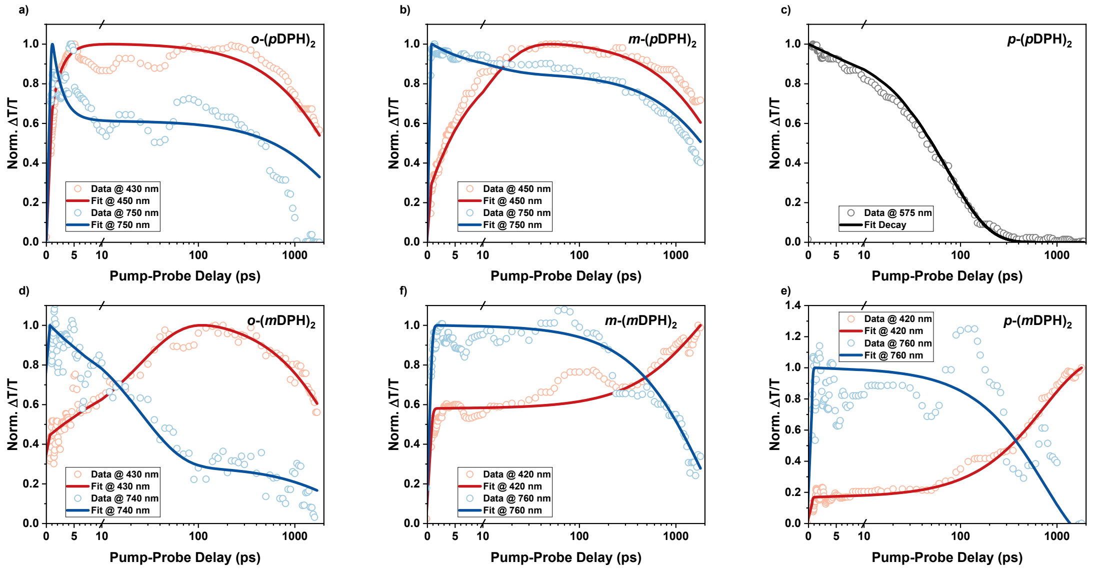

Figure S6. Comparison of the kinetics extracted at specified wavelengths from the raw fsTA data of the dimers and from the DAS fits of the data.

iv) nsTA: Spectra of dimers *o*-(*p*DPH)$_2$ and *m*-(*p*DPH)$_2$

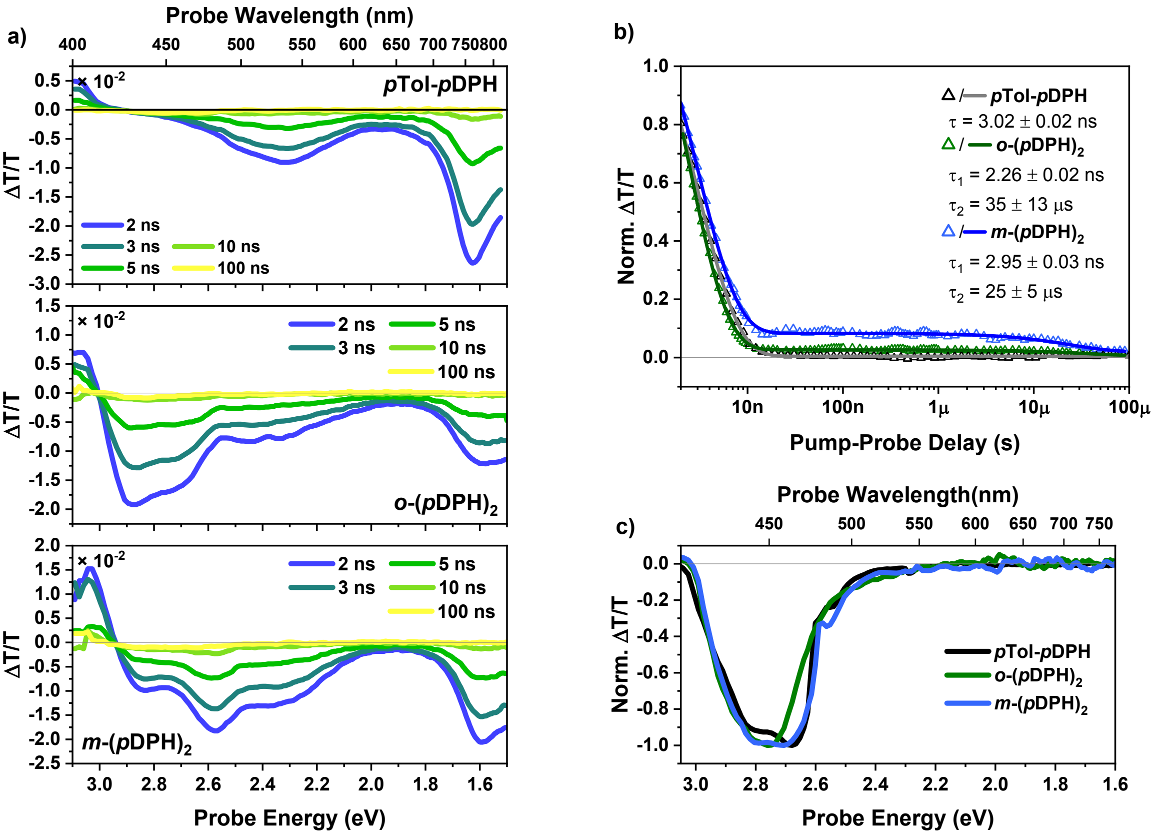

Figure S7. a) nsTA spectral intervals up to 100 ns of dilute (100 µM) solutions of *o*-(*p*DPH)$_2$ and *m*-(*p*DPH)$_2$ with *p*Tol-*p*DPH data shown for comparison. b) Kinetics for the data in (a) for the ranges 430-440 nm for the dimers and 530-540 nm for the monomer. Bi/monoexponential fits to the data are plotted. The dimer kinetics both have a long-lived, weak biexponential component, corresponding to isolated triplets, while the monomer kinetic is taken away from the region of the triplet PIA. c) Comparison of the triplet spectra of the *p*DPH dimers with the monomer, as produced through sensitization by PdOEP upon excitation at 532 nm.

v) nsTA: *m*DPH materials global analysis fitting

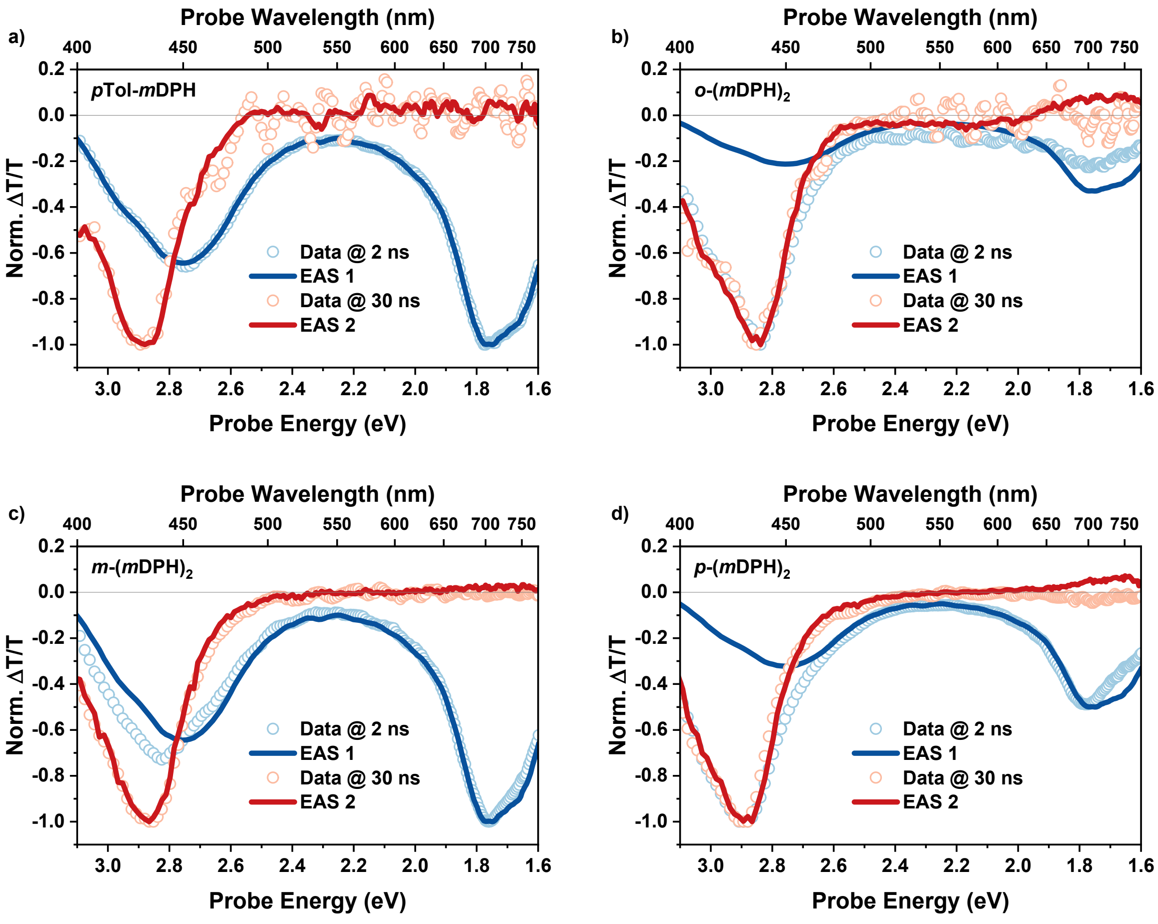

Figure S8. Evolution associated spectra (EAS) generated by a global analysis genetic algorithm[10] fitting for dilute solution nsTA data of the *m*DPH materials. Global analysis was found to perform better than DAS in respect to deconvolution of the triplet signal. While DAS enables control over the kinetic but no control over the species spectra, GA provides no handle over the kinetics but allows certain spectra to be fixed. For all of the dimers there is a mixture of singlet and triplet states formed within the instrument response, which DAS is unable to deconvolute as separate species. For GA the assumption was made that the singlet spectrum of the monomer should be only slightly different in shape than the singlet of any of the dimers; we fixed the early time spectrum of the monomer as a reference spectrum (EAS 1) that was not altered by the genetic algorithm, while EAS 2 (triplet) was optimized. This gave a good match for *m*-(*m*DPH)$_2$ but resulted it incomplete deconvolution for the isomers resulting in artificial positive features in red region for EAS 2 that are not real features of the triplet spectra. Nevertheless, fixing EAS 1 to the monomer singlet was required to prevent EAS 1 being optimized toward the initial spectra of these dimers and contain a significant amount of the triplet signal which would reduce the intensity of EAS 2 and skew the triplet yields which were calculated from the peak intensity of EAS 2.

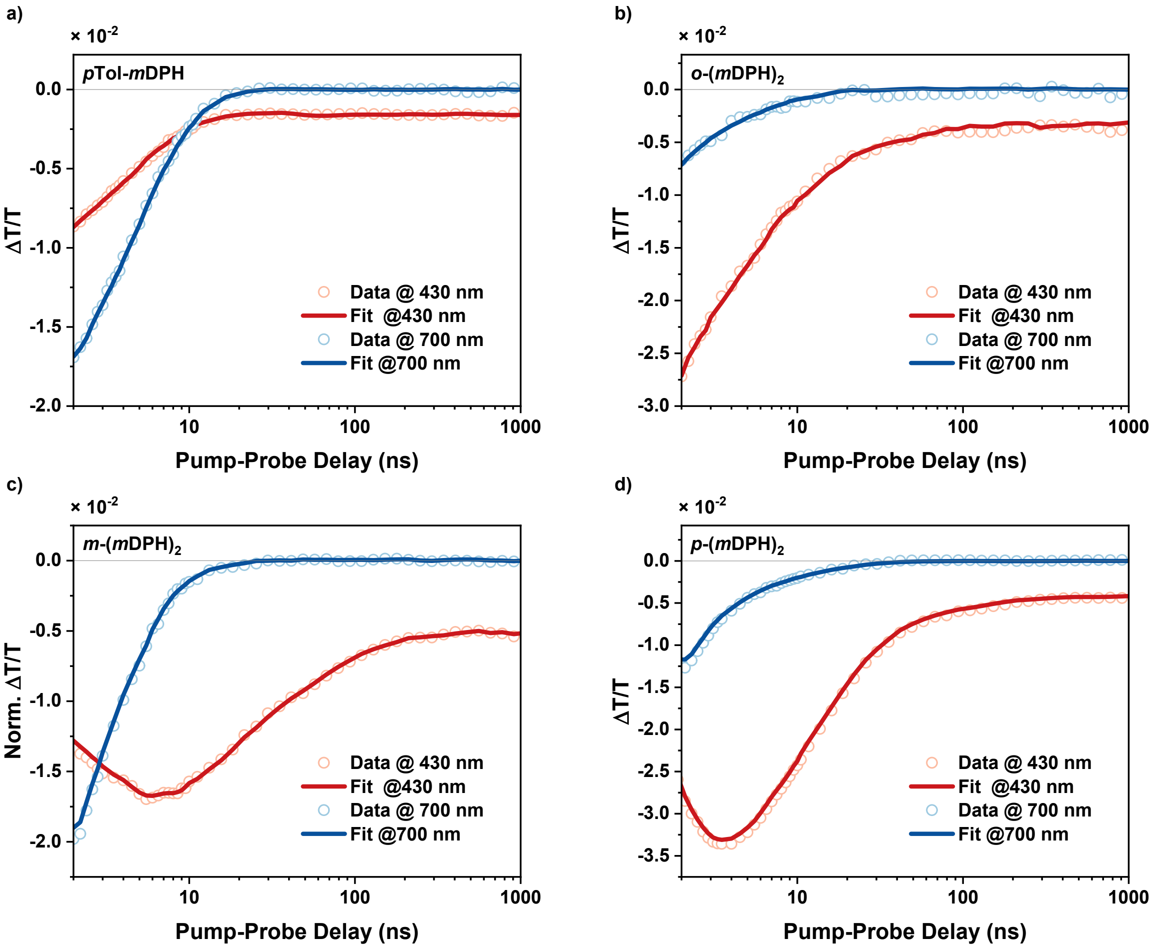

Figure S9. Comparison of the kinetics up to 1000 ns at two wavelengths of the nsTA data and the reconstructed fit spectra, as generated by the genetic algorithm.

vi) nsTA: *m*-(*m*DPH)$_2$ Concentration Series

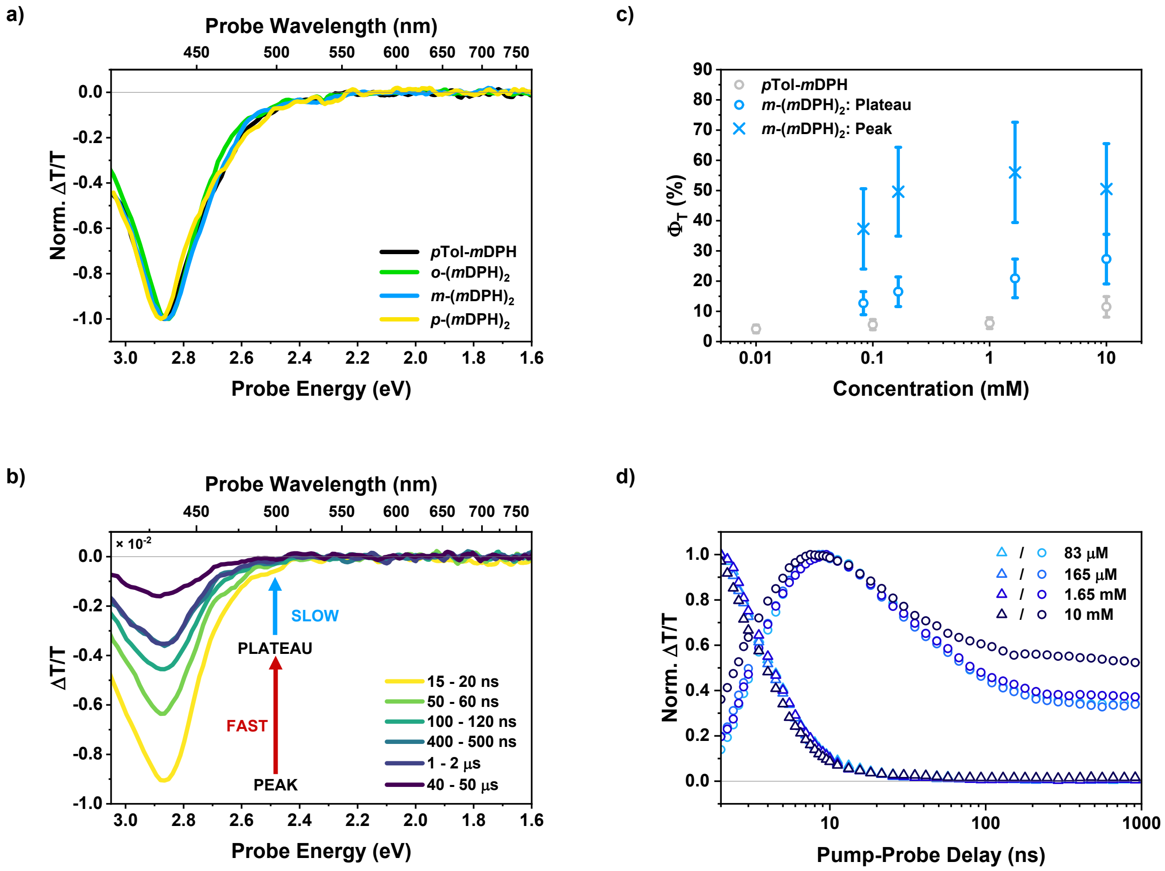

Figure S10. a) Normalized sensitized triplet spectra of the *m*DPH family of materials. b) nsTA spectra of *m*-(*m*DPH)$_2$ (83 μM), highlighting the two phases of triplet decay. c) Concentration dependence of the triplet quantum yield of *p*Tol-*m*DPH vs. *m*-(*m*DPH)$_2$ for both the peak and long-lived (plateau) triplet population. d) Kinetics of the singlet (EAS 1, triangles) and triplet (EAS 2, circles) extracted from the GA fits to the nsTA data of *m*-(*m*DPH)$_2$, over a concentration series.

5. Calculation of Triplet Quantum Yields

Triplet yields were calculated following a method derived from those reported in the singlet fission literature.[11,12] The calculation required analysis of data from the sensitization experiments alongside the nsTA data of the neat materials. Initial singlet populations were calculated directly from the absorbed laser power.

Sensitization experiments were carried out with ~100 µM of PdOEP with ~1-5 mM of the DPH acceptor being studied. A ~100 µM solution of PdOEP was measured as a control experiment. Sufficient acceptor concentration is essential in order to ensure efficient triplet energy transfer from the sensitizer. Under these conditions, the fitted timescales for triplet transfer were ~100 – 1000 ns. This is sufficiently faster than triplet decay of the neat sensitizer (>100 µs) or the acceptor (~50 µs) that the transfer efficiencies were treated as unity in all cases. Additionally, PdOEP is known to intersystem cross with near unity efficiency, so the population of triplets generated on the sensitizer was taken to be equal to the initial population of singlets as calculated from the absorbed fluence.

The sensitization data was utilized to estimate the triplet extinction coefficients by the following analytical workflow:

i) Power meter reading before the sample, after the sample and of the sample back reflection were used to calculate the laser power absorbed by the sample.

$$P_{Abs} = P_0 - P_{After} - P_{Reflected}$$

ii) $E_{Photon} = \frac{hc}{\lambda}$

iii) Laser repetition rate used to calculate number of absorbed photons per pulse:

$$Photons\ Abs/Pulse = \frac{P_{Abs}}{E_{Photon} \times "Rep.\ Rate"}$$

iv) Measurement of pump diameter, *d*, taken from beam profiler utilized to calculate absorption volume along with the cuvette path length, *l*.

$$V = l\pi\left(\frac{d}{2}\right)^2$$

v) From the assumptions discussed above:

$$"[T_1]" = [T_{1,acceptor}] = [T_{1,sensitizser}] = [S_{1,sensitizer}]$$

vi) Molar concentration of triplet states: $[T_1] = \frac{Photons\ Abs/Pulse}{V \times N_A}$

vii) $\frac{\Delta T}{T}$ for the wavelength (± 2 nm) corresponding to the DPH triplet peak is taken from the plateau of the kinetic of the sensitization data and converted to a change in absorbance.

$$\Delta A = -\log_{10}\left(\frac{\Delta T}{T} + 1\right)$$

viii) ΔA is divided by the concentration and pathlength (in cm) to get a triplet extinction coefficient with units of $M^{-1}.cm^{-1}$

$$\varepsilon^*(T) = \frac{\Delta A}{l \times [T_1]}$$

ix)

a. Repetition of measurements at different fluences allowed a **mean** taken for ε*(T). The standard deviation was typically significantly lower than the calculated fractional error. As such, the quoted errors are the mean fractional error (method below) for a single measurement rather than the standard deviation.
b. Within error ε*(T) was found to be equal for all *m*DPH materials. Given the strong qualitative match in sensitized triplet spectra and intuitive sense that the materials are based upon an identical chromophore it is reasonable to assume that ε* should be equal. A mean value was thus calculated for the *m*DPH family.

Estimate of the accompanying error, with application of standard rules for error propagation where appropriate:

i) Fluctuation of the laser power along with deviation in the power meter was estimated to give a fractional error in the power measurements of 0.05. This was based empirically on standard deviation of exact power meeting readings over the course of several measurements for which the attenuation of incoming laser power was not modified. This valued was applied as the estimated fractional error in the absorbed power, considering that the absorption and reflectivity of the samples represent absolute quantities such that the ratio $\frac{P_{Abs}}{P_0}$ should be fixed for any given sample.

$$\frac{\Delta(P_{Abs})}{P_{Abs}} = 0.05$$

ii) The second primary source of error is the measured beam diameter, with error, Δd, which was conservatively estimated upon analysis of the beam profile shape. We note that this represented the largest source of error by a significant margin in this work, yet is a factor that has been largely neglected by previous authors in the field.

iii) Treating photon energy and repetition rate as absolute constants, the error in the photons absorbed per pulse is considered as arising from the multiplication of P_{Abs} by constant factors and the fraction of power absorbed, $\frac{P_{Abs}}{P_0}$. Propagation of errors theory gives:

$$\frac{\Delta({}^{Photons\ Abs}/_{Pulse})}{{}^{Photons\ Abs}/_{Pulse}} = \sqrt{2 \times \left(\frac{\Delta(P_{Abs})}{P_{Abs}}\right)^2 + \left(\frac{\Delta(P_0)}{P_0}\right)^2} = \sqrt{3 \times 0.05^2} = 0.09$$

iv) Treating the error in the path length as negligible, the fractional error in the absorption volume is:

$$\frac{\Delta V}{V} = 2\frac{\Delta d}{d}$$

v) $$\frac{\Delta[T_1]}{T_1} = \sqrt{0.09^2 + \left(\frac{\Delta V}{V}\right)^2}$$

vi) The error in $\frac{\Delta T}{T}$ was estimated from the fluctuation of data points about the plateau position of the kinetic. $\Delta\left(\frac{\Delta T}{T}\right)$ was typically small but was accounted for. The error in the difference in absorbance was calculated: $\Delta(\Delta A) = \frac{\Delta\left(\frac{\Delta T}{T}\right)}{\left(\frac{\Delta T}{T}+1\right) \times \ln(10)}$

vii) $$\frac{\Delta\varepsilon^*(T)}{\varepsilon^*(T)} = \sqrt{\left(\frac{\Delta(\Delta A)}{\Delta A}\right)^2 + \left(\frac{\Delta[T_1]}{T_1}\right)^2}$$

	A	B	C	D
1		Green/Orange text = input row		
2		pTol-mDPH ~5mM/ PdOEP ~100 µM		Formulae
3	Power (uW)	385	814	input
4	Power After (uW)	318	680	input
5	Power Reflected (uW)	25	45	input
6	Power Absorbed(uW)	42	89	=D3-D4-D5
7	Estimated Fractional error in Power Measurement	0.05	0.05	input
8	Wavelength (nm)	532	532	input
9	E_photon (J)	3.73E-19	3.73E-19	=6.62607E-34*299792000/(D8*1e-9)
10	Laser rep rate (Hz)	500	500	input
11	Photons Abs/ Pulse	2.25E+11	4.77E+11	=1e-6*D6/(D10*D9)
12	Estimated fractional error in photons absorbed per pulse	0.09	0.09	=SQRT(3*D7^2)
13	Pump diameter (um)	891	891	input
14	Estimated Error in Pump Diameter (um)	100	100	input
15	Absorption Volume/ cm^3	6.23E-04	6.23E-04	=0.1*PI()*((D13/2)*10^-4)^2
16	Absorption Volume/ L	6.23E-07	6.23E-07	=D15/1000
17	**Singlet (triplet) Concentration/ µM**	**0.60**	**1.27**	=1e6*D11/(D$16*6.022140857*10^23)
18	**Estimated fractional Error in Singlet Concentration/ µM**	**0.24**	**0.24**	=SQRT(D12^2+(2*D14/D13)^2)
19	Maximum Triplet ΔT/T	-0.0108	-0.0233	input
20	Estimated Error in ΔT/T	0.00015	0.00010	input
21	ΔA	4.72E-03	1.02E-02	=-LOG10(D$19+1)
22	Error in ΔA	6.59E-05	4.45E-05	=D20/((D19+1)*LN(10))
23	Fractional Error in ΔA	1.40E-02	4.34E-03	=D22/D21
24	**Triplet Cross Section: ε*(T)/$M^{-1}.cm^{-1}$**	**7.87E+04**	**8.06E+04**	**=D21/(0.1*D17*10^-6)**
25	Estimated fractional error in ε*(T)/M-1.cm-1	0.24	0.24	=SQRT(D23^2+D18^2)
26				
27		Mean ε*(T)	Est. Fractional Error	
28	**pTol-mDPH**	**7.97E+04**	**0.24**	
29	**o-mDPH2**	**8.71E+04**	**0.24**	
30	**m-mDPH2**	**9.14E+04**	**0.24**	
31	**p-mDPH2**	**7.31E+04**	**0.24**	
32	**mDPH Series**	**8.28E+04**	**0.24**	

Figure S11. Representative example of the discussed calculation workflow for the sensitization of *p*Tol-*m*DPH, with demonstrative formulae indicated on the righthand side.

The workflow for calculation of the triplet yields from nsTA data was as follows:

i) [S_1] and accompanying fractional error are calculated from the laser power measurements and beam profile in an analogous manner to the calculation of [T_1]and its error from the sensitization.

ii) a. For peak triplet yield $\frac{\Delta T}{T}$ is taken from the peak of the relevant evolution associated spectrum as extracted from GA deconvolution of the nsTA data.

b. For long lived free triplet yield $\frac{\Delta T}{T}$ is taken from the plateau (~ 400 ns to 1 μs) of the EAS and an error estimated from the spread of values.

iii) ΔA and its error are calculated from $\frac{\Delta T}{T}$ by the same conversion formulae used above

iv)

a. Utilizing the cross section from sensitization the triplet concentration is estimated:

$$[T_1] = \frac{\Delta \mathrm{A}}{l \times \varepsilon^*(T)}$$

b. The fraction error is calculated from the fractional errors in ΔA and $\varepsilon^*(T)$:

$$\frac{\Delta[T_1]}{[T_1]} = \sqrt{\left(\frac{\Delta(\Delta A)}{\Delta A}\right)^2 + \left(\frac{\Delta\varepsilon^*(T)}{\varepsilon^*(T)}\right)^2}$$

v)

a. $\Phi_T\,/\,\% = 100 \times \frac{[T_1]}{[S_1]}$

b. $\frac{\Delta\Phi_T}{\Phi_T} = \sqrt{\left(\frac{\Delta[S_1]}{[S_1]}\right)^2 + \left(\frac{\Delta[T_1]}{T_1}\right)^2}$

Back-extrapolation of Φ_T from the instrument response limited value for *o*-(*m*DPH)$_2$

Amongst the *m*DPH dimers, for which Φ_T was calculated from the nsTA and sensitization data using the above method, *o*-(*m*DPH)$_2$ required further analysis to obtain a value of Φ_T truly representative of the peak triplet population. Unlike its isomers, the peak triplet yield does not occur on the timescale of the nsTA experiment. As such the value of Φ_T = 90 ± 27 %, calculated from the peak at 2 ns in the nsTA experiment, is instrument response limited peak and represents a lower bound for Φ_T. Instead, fast iSF results in the peak "TT" signal occurring at ~ 100 ps in the fsTA experiment. A time constant of 3.1 ± 0.3 ns was fit to the decay of the feature in the fsTA experiment, while a value of 3.9 ± 0.3 ns was fit as the dominant decay component of the triplet in the nsTA experiment. Together these values may be combined to suggest 3.5 ± 0.6 ns as a representative decay constant. The triplet population can be modelled as a function of time:

$$\Phi_T(\mathrm{t}) = \Phi_T(\mathrm{peak}) * e^{\frac{-(t - t_{peak})}{\tau}}$$

Which can be rearranged:

$$\Phi_T(\mathrm{peak}) = \Phi_T(\mathrm{t}) * e^{\frac{t - t_{peak}}{\tau}}$$

Taking, t = 2 ns, Φ_T(2 ns) = 90 ± 27 %, τ = 3.5 ns and t_{peak} = 0.1 ns:

$$\Phi_T(\mathrm{peak}) = (90 \pm 27) * e^{\frac{1.9}{3.5 \pm 0.6}} = 163 \pm 63\,\%$$

6. Synthetic Information

Intermediates

1-bromo-4-(2-butyloctyl)benzene (2)

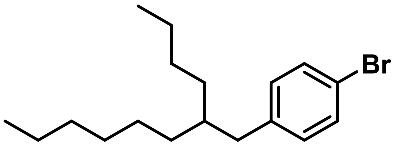

2

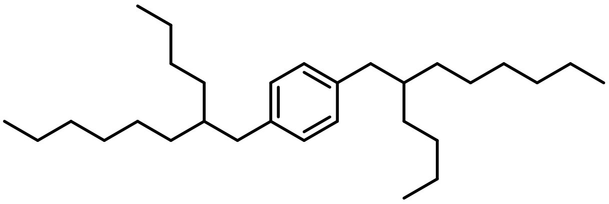

Minor By-product

Adapted from a literature procedure. [13]

Grignard Reagent

A single pellet of iodine was added to magnesium turnings (2.87 g, 0.12 mol, 1.2 eq.) in a flask fitted with reflux condenser and dropping funnel, which were ground by a stir bar under argon for ~ 20 h. A solution of 2-butyloctyl bromide (24.8 g, 99.5 mmol, 1.0 eq.) in dry diethyl ether (110 mL) was degassed and transferred to the dropping funnel by canula. This was added dropwise to the Mg/I mixture at room temperature and then heated to 40 ^{o}C to maintain steady reflux overnight. The mixture was allowed to cool to room temperature under Argon and then utilized directly in the kumada coupling step.

Kumada Coupling

To an oven-dried flask under an argon atmosphere and equipped with a dropping funnel and reflux condenser, was added 1,4-dibromobenzene **(1)** (19.4 g, 82 mmol, 0.82 equiv.), Pd(dppf)Cl_2 (0.432 g, 0.59 mmol, 0.006 equiv.), and anh. diethyl ether (~27 mL). The mixture was cooled to 0 ^{o}C. Then, the freshly prepared (2-butyoctyl)magnesium bromide solution was transferred to the dropping funnel by canula and added dropwise to the mixture. After addition was complete, the reaction was allowed to warm to RT and stir for 24 h. The resulting solution was poured onto ice water (~250 mL) and diethyl ether (~100 mL) and the mixture filtered through cotton wool. The organic phases were separated, washed with water, then dried ($MgSO_4$) and concentrated *in vacuo*. The resulting oil was purified by flash column chromatography (eluent: n-hexane) and the product containing fractions were concentrated *in vacuo.* Excess 1,4-dibromobenzene was removed from the oil by repeated (ca. 3 times) co-evaporation with DMSO *via* rotary evaporation (~14 mbar at 95 ^{o}C), and a subsequent aqueous workup with diethyl ether to remove any remaining DMSO. The product, **2**, was obtained as a colourless oil (17.5 g, 54.0 mmol, 66 %), pure except for ~ 3 % contamination by an inseparable minor product formed by double kumada coupling, *p*-di(2-butyloctyl)benzene. The material was used in the subsequent step at this level of purity with the by-product from the formation of **2** being easily separable from the aldehyde, **3**, by flash column chromatography.

^{1}H NMR (700 MHz, $CDCl_3$) *Major product: Minor product (Di-BO-Benzene), ratio: (1: 0.03);*
Major Product: δ 7.40 (d, J = 7.9 Hz, 2H), 7.03 (d, J = 7.9 Hz, 2H), 2.50 (d, J = 7.1 Hz, 2H), 1.59 (q, J = 6.1 Hz, 1H), 1.36 – 1.19 (m, 16H), 0.94 – 0.86 (m, 6H);

Minor product (Di-BO-Benzene): 7.05 (s, 4H), 2.51-2.50 (m, 4H), 1.57 – 1.47 (m, 2H), 1.37 – 1.13 (m, 16H), 0.89-0.86 (m, 12H).

^{13}C NMR (176 MHz, $CDCl_3$) δ 140.8, 131.1, 130.9, 119.2, 39.9, 39.6, 33.1, 32.8, 31.9, 29.7, 28.8, 26.5, 23.0, 22.7, 14.1, 14.1.

Minor product (Di-BO-Benzene): δ 128.8, 37.7, 33.7, 33.4, 32.0, 30.3, 29.8, 29.0, 26.7, 23.2, 22.7, 14.2.

1-bromo-4-(2-ethylhexyl)benzene (2b)

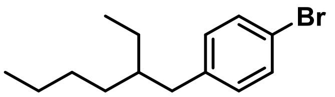

2b

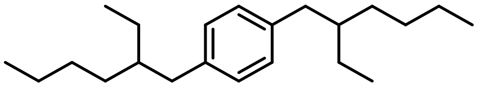

Minor By-product

1-bromo-4-(2-ethylhexyl)benzene was prepared using a Kumada coupling following the same detailed procedure given above for the butyloctyl analogue, **2**. In this case the Grignard reagent, 2-ethylhexyl magnesium bromide, used was commercial. The procedure was carried out on a greater scale than above utilizing 1,4-dibromobenzene (68 g, 288 mmol) and the product mixture was obtained as a colourless oil: Total- 62 g, 80% (major product: ~58 g, 75%; minor product ~3.8 g, 5%).

^{1}H NMR (700 MHz $CDCl_3$): *Major product: Minor product (Di-EH-Benzene), ratio: (1: 0.08);* Major product: δ 7.39 (d, *J* = 8.0 Hz, 2H), 7.02 (d, *J* = 8.0 Hz, 2H), 2.54 – 2.44 (m, 2H), 1.57 – 1.47 (m, 1H), 1.37 – 1.13 (m, 8H), 0.89-0.86 (m, 6H);

Minor product (Di-EH-Benzene): δ 7.05 (s, 4H), 2.54-2.44 (m, 4H), 1.57 – 1.47 (m, 2H), 1.37 – 1.13 (m, 16H), 0.89-0.86 (m, 12H).

^{13}C NMR (176 MHz, $CDCl_3$): Major product: δ 140.8, 131.1, 130.9, 119.2, 41.0, 39.5, 32.3, 28.8, 25.4, 23.0, 14.1, 10.8.

Minor product (Di-EH-Benzene): δ 138.9, 128.9, 41.1, 39.8, 32.4, 28.9, 25.5, 23.0, 14.1, 10.8.

HRMS(m/z) Found $[M]^+$ = 268.0817, $C_{14}H_{21}Br$ requires 268.0827, Δ = -3.7 ppm

4-(2-butyloctyl)benzaldehyde (3)

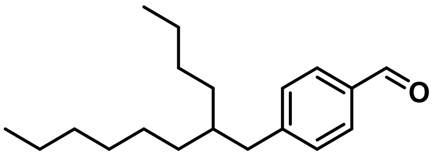

3

Adapted from a literature procedure. [14]

To an oven-dried flask equipped with a dropping funnel and under an argon atmosphere was added **2** (17.5 g, 54 mmol, 1.0 eq.) and anh. THF (~350 mL). The solution was cooled to -78 °C before *n*-butyl lithium (1.6 M in hexanes, 74 mL, 120 mmol, 2.2 equiv.) was added dropwise. The resulting mixture was allowed to stir at -78 °C for 1 h. Anh. DMF (10.5 mL, 135 mmol, 2.5 equiv.) was then added dropwise at -78 °C and the mixture was left to stir for a further 1 h at this temperature. The solution turned from clear and colourless to cloudy and white. The reaction was then quenched with ice water. The aqueous layer was separated and extracted with ethyl acetate (3 x 50 mL). The organic layers were combined and dried ($MgSO_4$) and then concentrated *in vacuo*. The crude clear yellow oil was then purified via column chromatography (eluent: DCM/ *n*-hexane gradient from 0:100 → 33:67 v/v), to obtain a yellow oil. NMR analysis revealed this to be the product contaminated by a by-product, 2-propylhept-2-enal, which was removed by Kugelrohr distillation (120 °C). Upon Kugelrohr distillation the product material darkened to brown, but NMR indicated a cleaner material than prior to distillation, with the major impurity removed. **3** was obtained as a brown oil (10.6 g, 39 mmol, 71 %).

^{1}H NMR (400 MHz, $CDCl_3$) δ 10.00 (s, 1H), 7.85 – 7.78 (m, 2H), 7.37 – 7.29 (m, 2H), 2.63 (d, J = 7.1 Hz, 2H), 1.68 (d, J = 8.4 Hz, 1H), 1.38 – 1.20 (m, 16H), 0.93 – 0.85 (m, 6H)

^{13}C NMR (176 MHz, $CDCl_3$) δ 192.0, 149.7, 134.4, 129.8, 129.7, 40.9, 39.6, 33.2, 32.9, 31.9, 29.6, 28.8, 26.5, 23.0, 22.7, 14.1

HRMS(m/z) Found [M+H]+ = 275.2372, $C_{19}H_{30}O$ requires 275.2375, Δ = -1.1 ppm

4-(2-butyloctyl)benzaldehyde (3b)

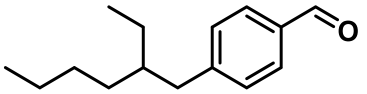

3b

Prepared by an analogous procedure to compound **3**, successfully on ~ double scale utilizing compound **2b** (31 g, 115 mmol). The crude clear yellow oil was purified *via* column chromatography (Eluent: Hexane initially, then 6:1 Hexane: Ethyl Acetate, gradually increasing polarity to 1:1 Hexane: Ethyl Acetate), to afford the title product as a clear and colourless oil (16.4 g, 66%).

^{1}H NMR (700 MHz, $CDCl_3$) δ 9.95 (d, *J* = 3.1 Hz, 1H), 7.77 (dd, *J* = 8.1, 2.9 Hz, 2H), 7.29 (dd, *J* = 8.1, 2.9 Hz, 2H), 2.59 (dd, *J* = 7.0, 2.8 Hz, 2H), 1.67–1.53 (m, 1H), 1.35–1.14 (m, 8H), 0.86 (m, 6H,).

^{13}C NMR (176, MHz $CDCl_3$) δ 191.9, 149.6, 134.4, 129.8, 129.7, 41.0, 40.4, 32.4, 28.8, 25.5, 23.0, 14.1, 10.7.

HRMS(m/z) Found [M+H]+ = 219.1747, $C_{15}H_{23}O$ requires 219.1743, Δ = 1.8 ppm

(E)-3-(4-(2-butyloctyl)phenyl)acrylaldehyde (4)

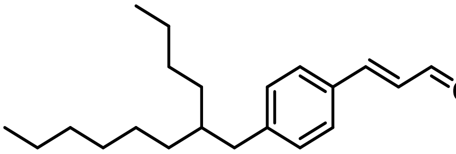

4

Adapted from a literature procedure. [15]

To an oven-dried flask under an argon atmosphere and equipped with a dropping funnel and reflux condenser was added (1,3-dioxolan-2-ylmethyl)triphenylphosphonium bromide (27.6 g, 64.0 mmol, 2.5 equiv.), and anh. THF (~660 mL) via cannula. To this was added lithium methoxide solution in methanol (2.2 M, 30 mL, 66 mmol, 2.6 equiv.). The suspension was heated to reflux (80 °C) for 30 mins. To a separate oven-dried flask under argon was added **3** (7.03 g, 25.6 mmol, 1.0 equiv.) and anh. THF (~300 mL). This solution was transferred to the dropping funnel and added to reaction mixture over 30-60 mins. The mixture was heated to reflux and allowed to stir for 24 h. The reaction was cooled to RT, at which point 10% aq. HCl solution (100 mL) was added. Stirring was continued for 1 hr to hydrolyse intermediate acetals to the all-trans aldehydes. Colour change observed was from cloudy cream to clear and pale yellow. The organic layer was then separated, and the aqueous layer extracted with DCM. Combined organic layers were subsequently washed with water, sat. aq. sodium bicarbonate solution and brine, before being dried ($MgSO_4$) and concentrated *in vacuo*. The crude material was then

stirred in *n*-hexane (~ 500 mL) overnight and filtered through celite to remove insoluble phosphine oxide by-product. The filtrate was concentrated *in vacuo* and purified by flash column chromatography (eluent: DCM/ *n*-hexane gradient from 0:100 → 26:74 v/v), to give the title product, **4**, as a pale yellow oil, (6.90 g, 23 mmol, 89%), following removal of the solvent.

^{1}H NMR (700 MHz, $CDCl_3$) δ 9.72 (d, J = 7.7 Hz, 1H), 7.51 (d, J = 8.3 Hz, 2H), 7.49 (d, J = 16.6 Hz, 1H), 7.23 (d, J = 7.9 Hz, 2H), 6.72 (dd, J = 15.9, 7.7 Hz, 1H), 2.59 (d, J = 7.1 Hz, 2H), 1.66 (h, J = 6.2 Hz, 1H), 1.36 – 1.21 (m, 16H), 0.90 (t, J = 6.9 Hz, 6H)

^{13}C NMR (176 MHz, $CDCl_3$) δ 193.8, 153.0, 146.2, 131.5, 130.0, 128.4, 127.7, 40.6, 39.6, 33.2, 32.9, 31.9, 29.6, 28.8, 26.5, 23.0, 22.7, 14.1, 14.1

HRMS(m/z) Found $[M]^+$ = 300.24532, $C_{21}H_{32}O$ requires 300.2450, Δ = -1.1 ppm

(E)-3-(4-(2-ethylhexyl)phenyl)acrylaldehyde (4b)

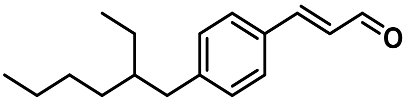

4b

Prepared by an analogous procedure to compound **4** on similar scale, using **3b** (5.03 g, 23.0 mmol, 1.0 equiv.). The crude product was dry loaded onto silica before being subjected to column chromatography (Hexane: EtOAc, 9:1), to give the title product as a clear golden yellow oil, (3.8 g, 68%).

^{1}H NMR (700 MHz, $CDCl_3$) δ 9.69 (d, *J* = 7.7 Hz, 1H), 7.48 (d, *J* = 8.1 Hz, 2H), 7.46 (d, *J* = 16.1 Hz, 1H), 7.22 (d, *J* = 8.1 Hz, 2H), 6.70 (dd, *J* = 16.1, 7.7 Hz, 1H), 2.57 (dd, *J* = 7.1, 2.7 Hz, 2H), 1.58 (m, 1H), 1.34 – 1.18 (m, 8H), 0.88 (t, J = 7.4 Hz, 6H).

^{13}C NMR (126 MHz, $CDCl_3$): 193.9, 153.1, 146.2, 131.5, 130.0, 128.5, 127.7, 41.1, 40.2, 32.3, 28.8, 25.5, 23.0, 14.1, 10.8.

HRMS(m/z) Found [M+H]+ = 245.1916, $C_{17}H_{25}O$ requires 245.1905, Δ = 4.5 ppm

(2E,4E)-5-(4-(2-butyloctyl)phenyl)penta-2,4-dienal (5)

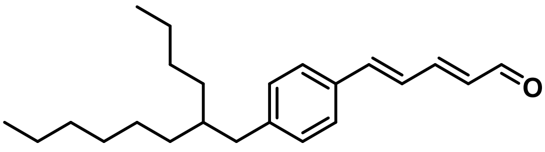

5

5 was prepared by analogous procedure to **4**, utilizing **4** as the starting material. This corresponded to 0.9 times the molar scale for all reagents. In this case the crude material was purified by flash column chromatography with a different solvent system (eluent: EtOAc/ *n*-hexane gradient from 0:100 → 3:97 v/v). The product, **5**, was obtained as a yellow oil (6.65 g, 20.4 mmol, 87 %). *NB: Keep under vacuum or argon until ready to use as decomposition is possible.*

^{1}H NMR (700 MHz, $CDCl_3$) δ 9.64 (d, J = 7.9 Hz, 1H), 7.46 – 7.42 (m, 2H), 7.29 (dd, J = 15.2, 9.8 Hz, 1H), 7.20 – 7.16 (m, 2H), 7.03 (d, J = 15.5 Hz, 1H), 7.00 (dd, J = 15.5, 9.8 Hz, 1H), 6.28 (dd, J = 15.2, 7.9 Hz, 1H), 2.57 (d, J = 7.1 Hz, 2H), 1.64 (m, 1H), 1.36 – 1.22 (m, 16H), 0.91 – 0.85 (m, 6H)

^{13}C NMR (176 MHz, $CDCl_3$) δ 193.6, 152.4, 144.3, 142.7, 133.0, 131.1, 129.8, 127.4, 125.3, 40.5, 39.6, 33.2, 32.9, 31.9, 29.7, 28.8, 26.5, 23.0, 22.7, 14.1, 14.1

HRMS(m/z) Found [M+H]+ = 327.2681, $C_{23}H_{34}O$ requires 327.2688, Δ = -2.1 ppm

(2E,4E)-5-(4-(2-ethylhexyl)phenyl)penta-2,4-dienal (5b)

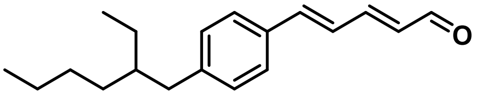

5b

5b was prepared by analogous procedure to **4**, utilizing **5** as the starting material. The procedure was scaled up, utilizing **4b** (13.4 g, 54.8 mmol, 1.0 equiv.), and performed at higher concentration without scaling the amount of solvent. The crude product was dry loaded onto silica before being subjected to column chromatography (Hexane: EtOAc, 9:1), to give the title product as a clear, golden yellow oil, (10.0 g, 72%). *NB: Keep under vacuum or argon until ready to use as decomposition is possible.*

^{1}H NMR (700 MHz, $CDCl_3$) δ 9.60 (d, *J* = 7.9 Hz, 1H), 7.41 (d, *J* = 8.2 Hz, 2H), 7.25 (dd, *J* = 15.2, 9.9 Hz, 1H), 7.16 (d, *J* = 8.2 Hz, 2H), 7.00 – 6.94 (m, 2H), 6.24 (dd, *J* = 15.2, 7.9 Hz, 1H), 2.59 – 2.50 (m, 2H), 1.62 – 1.54 (m, 1H), 1.35 – 1.20 (m, 8H), 0.88 (m, 6H).

^{13}C NMR (176 MHz, $CDCl_3$): 193.5, 152.4, 144.2, 142.6, 133.1, 131.1, 129.8, 127.4, 125.3, 41.1, 40.1, 32.4, 28.8, 25.5, 23.0, 14.2, 10.8.

HRMS(m/z) Found [M+H]+ = 271.2054, $C_{19}H_{27}O$ requires 271.2062, Δ = -2.9 ppm

4-bromobenzyl triphenylphosphonium bromide (7)

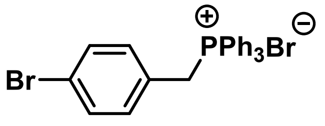

7

4-bromobenzyl bromide **(6)** (10 g, 40 mmol, 1.0 eq.) and triphenylphosphine (12.6 g, 48 mmol, 1.2 eq.) were heated to reflux in toluene (390 mL) overnight. After the reaction mixture was allowed to cool to room temperature it was filtered, and the precipitate washed with toluene and dried under suction. **7** was obtained as a white powder (18.9 g, 37 mmol, 93 %). Due to low solubility ^{13}C NMR spectrum could not be obtained. ^{1}H NMR data were in accordance with those reported in the literature.[16]

^{1}H NMR (400 MHz, $CDCl_3$) δ 7.85 – 7.77 (m, 9H), 7.71 – 7.61 (m, 6H), 7.30 – 7.22 (m, 2H), 7.11 – 7.03 (m, 2H), 5.56 (d, J = 14.6 Hz, 2H)

3-bromobenzyl triphenylphosphonium bromide (7b)

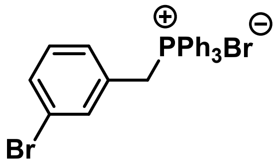

7b

Adapted from a literature procedure.[17]

To a flask set up for reflux was added 3-bromobenzylbromide (12.8 g, 51.2 mmol, 1 equiv.), triphenylphosphine (16.2 g, 61.8 mmol, 1.2 equiv.) and toluene (400 mL). The mixture was refluxed at 110 °C for 18 h. The reaction was then cooled to RT, to 0°C and the resulting precipitate was filtered under vacuum. The filter cake was washed with toluene (x2) and hexanes (x2) and then dried under vacuum to afford the title product as a white powder, (25.7 g, 98%).

^{1}H NMR (400 MHz $CDCl_3$) δ 7.82 – 7.72 (m, 9H), 7.66 – 7.56 (m, 6H), 7.34 – 7.27 (m, 2H), 6.99 (td, J = 7.9, 1.0 Hz, 1H), 6.94 (q, J = 2.1 Hz, 1H), 5.54 (d, J = 14.7 Hz, 2H).

^{13}C NMR (101 MHz, $CDCl_3$) δ 135.1, 135.1, 134.5, 134.4, 134.0, 134.0, 131.4, 131.4, 130.7, 130.6, 130.4, 130.3, 130.3, 130.1, 129.7, 129.7, 122.3, 122.3, 118.0, 117.2, 30.6, 30.1.

HRMS(m/z) Found $[M-Br]^{+}$= 431.0561, $C_{25}H_{21}PBr$ requires 431.0564, Δ = -0.7 ppm

1-bromo-4-((1E,3E,5E)-6-(4-(2-butyloctyl)phenyl)hexa-1,3,5-trien-1-yl)benzene (Br-*p*DPH)

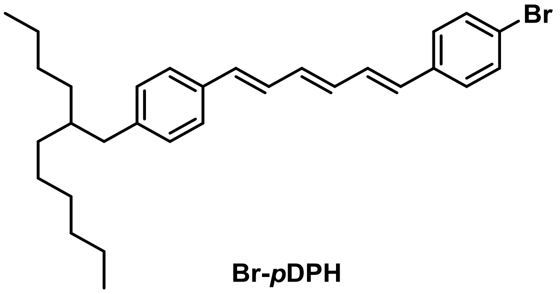

Br-*p*DPH

Br-*p*DPH was synthesized by a Wittig reaction of pentadienal **5** and phosphonium salt **7**. Sodium hydride (60 % dispersion in mineral oil, 282 mg, 7.04 mmol, 2.3 eq.) was dissolved in dry THF (70 mL) and cooled to 0 °C using an ice bath, under argon. Under a positive flow of argon **7** (1.96 g, 3.83 mmol, 1.25 eq.) was added as a single solid portion. The ice bath was removed, and the suspension was stirred at room temperature for 15 minutes. Colour change from the immediately formed yellow suspension to orange was observed. A solution of **5** (1.0 g, 3.1 mmol, 1.0 eq.) in dry THF (65 mL) was prepared and added dropwise and the mixture stirred overnight, in the dark. The mixture was poured over ice/water, extracted with EtOAc, washed with 0.1 M HCl (10 mL) and brine (10 mL), dried ($MgSO_4$) and the solvent removed *in vacuo.* The solid material was sonicated in methanol, filtered, washed with more methanol and dried under suction. **Br- *p*DPH** was obtained as a pale-yellow powder (0.943 g, 1.96 mmol, 64 %).

^{1}H NMR (700 MHz, $CDCl_3$) δ 7.46 (d, J = 8.5 Hz, 2H), 7.35 (d, J = 8.1 Hz, 2H), 7.29 (d, J = 8.5 Hz, 2H), 7.13 (d, J = 8.0 Hz, 2H), 6.89 (t, J = 10.7 Hz, 1H), 6.87 (t, J = 10.6 Hz, 1H), 6.63 (d, J = 15.5 Hz, 1H), 6.56 (dd, J = 14.7, 10.5 Hz, 1H), 6.53 (d, J = 15.7 Hz, 1H), 6.50 (dd, J = 14.8, 10.5 Hz, 1H), 2.54 (d, J = 7.0 Hz, 2H), 1.63 (p, J = 6.1 Hz, 1H), 1.36 – 1.23 (m, 16H), 0.91 (td, J = 7.1, 1.9 Hz, 6H)

^{13}C NMR (176 MHz, $CDCl_3$) δ 141.8, 136.4, 134.6, 134.5, 133.3, 132.5, 131.7, 130.8, 130.0, 129.6, 128.0, 127.7, 126.2, 121.1, 40.4, 39.7, 33.2, 32.9, 31.9, 29.7, 28.8, 26.6, 23.1, 22.7, 14.2, 14.1

HRMS(m/z) Found [M+H]+ = 479.2318, $C_{30}H_{39}Br$ requires 479.2313, Δ = 1.0 ppm

1-bromo-3-((1E,3E,5E)-6-(4-(2-ethylhexyl)phenyl)hexa-1,3,5-trien-1-yl)benzene (Br-*m*DPH)

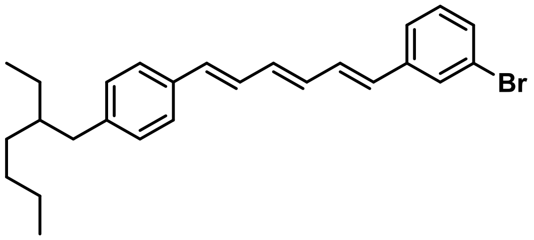

Br-*m*DPH

Br-*m*DPH was synthesized by an analogous procedure to **Br-*p*DPH**, on approximately triple scale using **5b** (2.26 g, 8.36 mmol, 1 equiv.) and **7b** (5.37 g, 10.5 mmol, 1.25 equiv.). However, the solvent volumes were not scaled and as such a higher concentration was employed. The initial colour change observed was from bright orange to copper.

The crude product was then dry loaded onto silica before being subjected to column chromatography (Hexane: DCM, 2:1). Fractions containing the product were combined and concentrated *in vacuo* before being sonicated in methanol and filtered under vacuum to give the title product as a pale- yellow powder (1.4 g, 32%).

^{1}H NMR (400 MHz, $CDCl_3$) δ 7.56 (t, *J* = 1.8 Hz, 1H), 7.43 – 7.27 (m, 4H), 7.18 (t, *J* = 7.8 Hz, 1H), 7.14 – 7.05 (m, 2H), 6.86 (ddd, *J* = 15.5, 10.0, 7.8 Hz, 2H), 6.67 – 6.33 (m, 4H), 2.52 (dd, *J* = 6.7, 2.3 Hz, 2H), 1.57 (d, *J* = 6.7 Hz, 1H), 1.36 – 1.18 (m, 8H), 0.87 (ddt, *J* = 7.5, 5.7, 2.3 Hz, 6H).

^{13}C NMR (101 MHz, $CDCl_3$): δ 141.9, 139.7, 134.9, 134.6, 133.54, 132.4, 130.7, 130.5, 130.2, 130.1, 129.6, 129.0, 128.0, 126.3, 124.9, 122.9, 41.1, 39.9, 32.4, 28.9, 25.4, 23.1, 14.2, 10.8.

HRMS(m/z) Found [M+H]+ = 423.1698, $C_{26}H_{32}Br$ requires 423.1687, Δ = 2.6 ppm

Final Compounds

4-((1E,3E,5E)-6-(4-(2-butyloctyl)phenyl)hexa-1,3,5-trien-1-yl)-4'-methyl-1,1'-biphenyl (*p*Tol-*p*DPH)

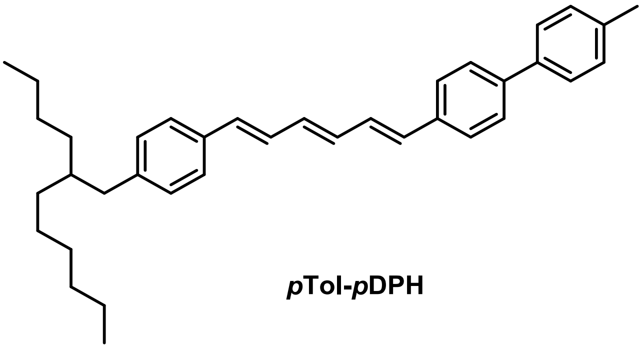

***p*Tol-*p*DPH**

***p*Tol-*p*DPH** was synthesized by a Suzuki coupling of **Br- *p*DPH** and *p*-tolyl boronic acid.

A 10 mL microwave vial was charged with a stir bar, **Br- *p*DPH** (65 mg, 0.135 mmol, 1.0 eq.), *p*-tolyl boronic acid (22 mg, 0.16 mmol, 1.2 eq.), Na_2CO_3 (30 mg, 0.28 mmol, 2 eq.) and $Pd(PPh_3)_2Cl_2$ (3 mg, 3 mol %) and flush under argon. THF (5 mL) and water (0.5 mL) were separately degassed and then added to the solid reagents. The reaction mixture was lowered into a preheated oil bath and heated at 65 °C in the dark for 24 h. The reaction mixture was diluted with DCM (75 mL) and brine (75 mL) and the layers separated. The aqueous layer was extracted with further DCM (2 x 30 mL) and the combined organic phase dried ($MgSO_4$) and the solvent removed *in vacuo* to obtain the crude product. The crude material was purified by flash column chromatography (eluent: gradient *n*-hexane → DCM/ *n*-hexane 0.11/0.98 v/v) and following sonication and filtration from methanol, ***p*Tol-*p*DPH** obtained as a yellow-green powder (42 mg, 0.086 mmol, 63 %).

^{1}H NMR (700 MHz, $CDCl_3$) δ 7.59 – 7.57 (m, 2H), 7.56 – 7.52 (m, 2H), 7.52 – 7.48 (m, 2H), 7.36 (d, J = 8.1 Hz, 2H), 7.30 – 7.26 (m, 2H), 7.13 (d, J = 8.0 Hz, 2H), 6.98 – 6.93 (m, 1H), 6.92 – 6.87 (m, 1H), 6.64 (t, J = 15.6 Hz, 2H), 6.60 – 6.51 (m, 2H), 2.55 (d, J = 7.0 Hz, 2H), 2.43 (s, 3H), 1.64 (dt, J = 12.3, 6.5 Hz, 1H), 1.39 – 1.22 (m, 16H), 0.95 – 0.89 (m, 6H).

^{13}C NMR (176 MHz, $CDCl_3$) δ 141.7, 140.08, 137.8, 137.1, 136.3, 134.8, 133.9, 133.1, 132.8, 131.9, 129.5, 129.5, 129.2, 128.2, 127.1, 126.8, 126.7, 126.2, 40.4, 39.7, 33.2, 32.8, 31.9, 29.7, 28.8, 26.6, 23.1, 22.7, 21.1, 14.2, 14.1

HRMS(m/z) Found [M+H]+ = 490.3602, $C_{37}H_{46}$ requires 490.3600, Δ = 0.4 ppm

3-((1E,3E,5E)-6-(4-(2-ethylhexyl)phenyl)hexa-1,3,5-trien-1-yl)-4'-methyl-1,1'-biphenyl (*p*Tol-*m*DPH)

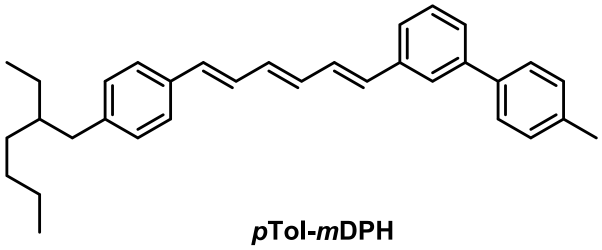

***p*Tol-*m*DPH**

A 10 mL microwave vial was charged with a stir bar, **Br- *m*DPH** (70 mg, 0.17 mmol, 1.0 eq.), *p*-tolyl boronic acid (31 mg, 0.22 mmol, 1.3 eq.) and $Pd(PPh_3)_4$ (8 mg, 0.007 mmol, 5 mol %) and flushed under argon. THF (5 mL) and an aqueous solution of Na_2CO_3 (2M, 0.5 mL) were separately degassed and then added to the solid reagents. The reaction mixture was lowered into a preheated oil bath and heated at 65 °C in the dark for 2 days. The reaction mixture was carefully acidified with 1 M HCl (~ 2 mL) with stirring and then diluted with DCM (~50 mL) and brine (~30 mL). The organic layer was separated and washed with brine (~ 30 mL), dried ($MgSO_4$) and the solvent remove *in vacuo*. The crude material was then purified by flash column chromatography (eluent: DCM/ *n*-hexane 1:9 v/v). Following removal of the solvent the columned material was sonicated in methanol and filtered to obtain the product. ***p*Tol-*m*DPH**, was obtained as a cream-coloured powder (47 mg, 0.11 mmol, 65 %).

^{1}H NMR (500 MHz, $CDCl_3$) δ 7.62 (s, 1H), 7.53 (d, J = 8.0 Hz, 2H), 7.45 (m, 2H), 7.36 (m, 1H), 7.40 (m, 2H), 7.35 (d, J = 8.0 Hz, 2H), 7.29 (d, J = 8.0 Hz, 2H), 7.13 (d, J = 8.1 Hz, 2H), 6.99 – 6.94 (m, 1H), 6.91 – 6.86 (m, 1H), 6.67 (d, J = 15.5 Hz, 1H), 6.61 (d, J = 15.5 Hz, 1H), 6.56 – 6.53 (m, 2H), 2.56-2.52 (m, 2H), 2.43 (s, 3H), 1.59 (m, 1H), 1.35 – 1.22 (m, 8H), 0.93 – 0.85 (m, 6H).

^{13}C NMR (126 MHz, $CDCl_3$) δ 141.7, 141.6, 138.2, 137.9, 137.2, 134.7, 134.0, 133.0, 132.9, 132.3, 129.5, 129.5, 129.0, 128.2, 127.0, 126.2, 125.1, 124.9, 41.1, 39.9, 32.4, 28.9, 25.4, 23.1, 21.1, 14.2, 10.8.

HRMS(m/z) Found [M+H]+ = 435.3042, $C_{33}H_{38}$ requires 435.3052, Δ = -2.3 ppm

4,4''-bis((1E,3E,5E)-6-(4-(2-butyloctyl)phenyl)hexa-1,3,5-trien-1-yl)-1,1':3',1''-terphenyl (*m*-(*p*DPH)$_2$)

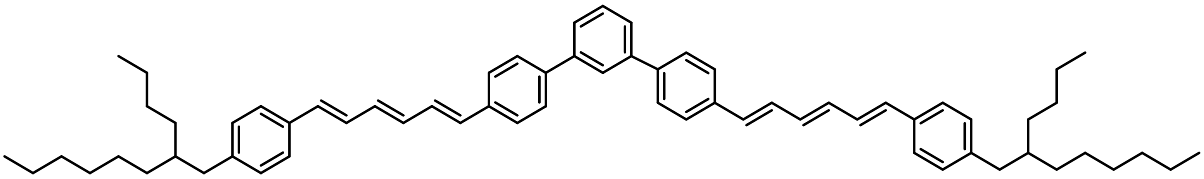

***m*-(*p*DPH)$_2$**

Dimer ***m*-(*p*DPH)$_2$** was synthesized by a Suzuki coupling of **Br-*p*DPH** and benzene-1,3-diboronic acid bispinacol ester.

A 20 mL microwave vial was charged with a stir bar, **Br-*p*DPH** (164 mg, 0.34 mmol, 2.1 eq.), benzene-1,3-diboronic acid bispinacol ester (53 mg, 0.16 mmol, 1.0 eq.) and $Pd(PPh_3)_4$ (17 mg, 0.016 mmol, 0.1 eq.) and flushed under argon. THF (10 mL) and an aqueous solution of Na_2CO_3 (2M, 1 mL) were separately degassed and then added to the solid reagents. The reaction mixture was lowered into a preheated oil bath and heated at 65 °C in the dark for 2 days. The reaction mixture was carefully acidified with 1 M HCl (~ 4 mL) with stirring and then diluted with DCM (~100 mL) and brine (~75 mL). The organic layer was separated and washed with brine (~ 30 mL), dried ($MgSO_4$) and the solvent remove *in vacuo*. The crude material was then purified by flash column chromatography (eluent: DCM/ *n*-hexane 1:9 v/v). Following removal of the solvent the columned material was sonicated in methanol and filtered to obtain the product. ***m*-(*p*DPH)$_2$**, was obtained as a yellow powder (76.4 mg, 0.087 mmol, 55 %).

^{1}H NMR (700 MHz, $CDCl_3$) δ 7.85 (d, *J* = 2.0 Hz, 1H), 7.65 (d, *J* = 7.9 Hz, 4H), 7.60 (dd, *J* = 7.6, 1.8 Hz, 2H), 7.53 (d, *J* = 7.9 Hz, 5H), 7.36 (d, *J* = 7.7 Hz, 4H), 7.13 (d, *J* = 7.7 Hz, 4H), 7.00 – 6.94 (m, 2H), 6.92 – 6.87 (m, 2H), 6.65 (dd, *J* = 22.5, 15.5 Hz, 4H), 6.60 – 6.53 (m, 4H), 2.55 (d, *J* = 6.9 Hz, 4H), 1.64 (p, *J* = 6.1 Hz, 2H), 1.38 – 1.21 (m, 32H), 0.95 – 0.87 (m, 12H).

^{13}C NMR (176 MHz, $CDCl_3$) δ 141.7, 141.3, 140.1, 136.8, 134.8, 134.0, 132.98, 133.0, 131.8, 129.5, 129.5, 129.2, 128.2, 127.4, 126.8, 126.2, 125.9, 125.5, 40.4, 39.7, 33.2, 32.9, 31.9, 29.7, 28.8, 26.6, 23.0, 22.7, 14.1, 14.1.

HRMS(m/z) Found [M+H]+ = 875.6494, $C_{66}H_{82}$ requires 875.6495, Δ = -0.1 ppm

4,4''-bis((1E,3E,5E)-6-(4-(2-butyloctyl)phenyl)hexa-1,3,5-trien-1-yl)-1,1':4',1''-terphenyl (*p*-(*p*DPH)$_2$)

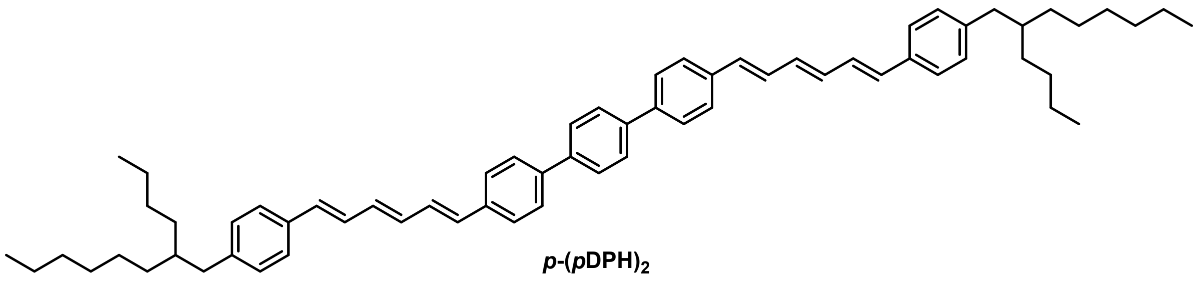

***p*-(*p*DPH)$_2$**

Dimer ***p*-(*p*DPH)$_2$** was synthesized by an analogous Suzuki coupling to ***m*-(*p*DPH)$_2$**, on the same scale, utilizing the isomeric starting material 1,4-diboronic acid bispinacol ester. The product was less soluble requiring modification of the work up procedure.

The reaction mixture was carefully acidified with 1 M HCl (~ 4 mL) with stirring and then filtered. The precipitate was washed with THF, 1 M HCl, water and methanol before being recrystallized from boiling chloroform (~ 50 mL). The product, ***p*-(*p*DPH)$_2$**, was obtained as a poorly soluble yellow powder (66.9 mg, 0.076 mmol, 48 %).

^{1}H NMR (500 MHz, *d_2*-TCE, 70 °C) δ 7.75 (s, 4H), 7.69 (d, J = 8.0 Hz, 4H), 7.56 (d, J = 7.9 Hz, 4H), 7.39 (d, J = 7.7 Hz, 4H), 7.17 (d, J = 7.8 Hz, 4H), 6.99 (dd, J = 15.5, 9.0 Hz, 2H), 6.91 (dd, J = 15.6, 9.0 Hz, 2H), 6.68 (t, J = 16.1 Hz, 4H), 6.60 (d, J = 6.8 Hz, 4H), 2.59 (d, J = 6.8 Hz, 4H), 1.68 (s, 2H), 1.33 (d, J = 8.4 Hz, 32H), 0.95 (t, J = 6.6 Hz, 12H).

Insufficient solubility for ^{13}C NMR spectrum to be obtained.

HRMS(m/z) Found [M+H]+ = 875.6470, $C_{66}H_{82}$ requires 875.6495, Δ = -2.9 ppm

4,4''-bis((1E,3E,5E)-6-(4-(2-butyloctyl)phenyl)hexa-1,3,5-trien-1-yl)-1,1':2',1''-terphenyl (*o*-(*p*DPH)$_2$)

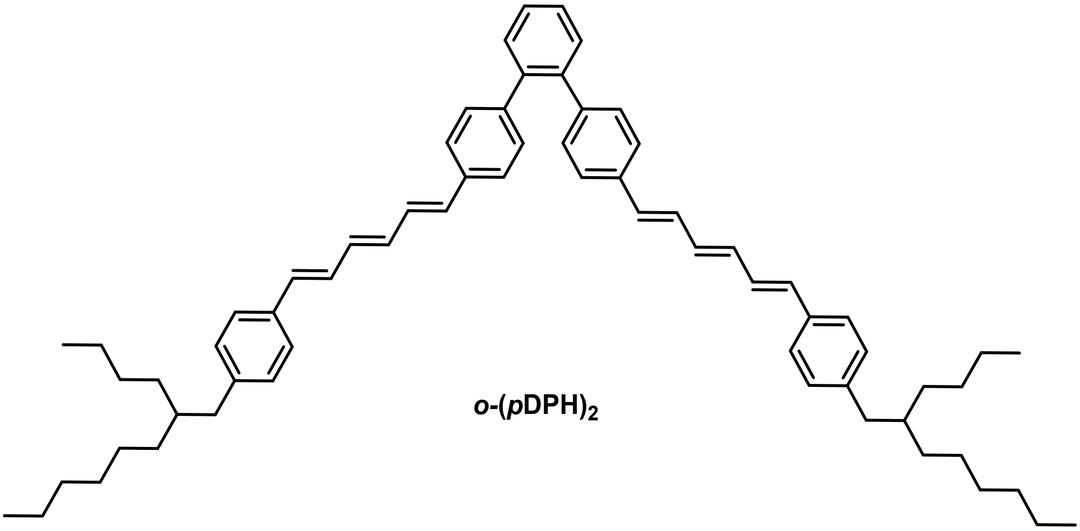

Dimer ***o*-(*p*DPH)$_2$** was synthesized by an analogous Suzuki coupling to ***m*-(*p*DPH)$_2$**, on the same scale, utilizing the isomeric starting material 1,2-diboronic acid bispinacol ester. The product still contained trace impurities after the MeOH wash step, requiring modification of the work up procedure.

Additionally following the MeOH wash step the material was recrystallized from EtOH/ $CHCl_3$ (~25 ml/ ~5 ml) to obtain the product, ***o*-(*p*DPH)$_2$**, as a yellow powder (59.8 mg, 0.068 mmol, 43 %).

^{1}H NMR (700 MHz, $CDCl_3$) δ 7.45 (dq, *J* = 18.2, 4.3 Hz, 4H), 7.34 (d, *J* = 7.8 Hz, 4H), 7.31 (d, *J* = 7.9 Hz, 4H), 7.13 (dd, *J* = 16.2, 7.8 Hz, 8H), 6.91 – 6.84 (m, 4H), 6.59 (dd, *J* = 21.6, 15.5 Hz, 4H), 6.56 – 6.48 (m, 4H), 2.54 (d, *J* = 7.0 Hz, 4H), 1.63 (p, *J* = 6.2 Hz, 2H), 1.29 (dq, *J* = 21.5, 7.1 Hz, 32H), 0.91 (t, *J* = 7.2 Hz, 12H).

^{13}C NMR (176 MHz, $CDCl_3$) δ 141.6, 140.7, 140.10, 135.7, 134.8, 133.8, 133.0, 132.8, 132.0, 130.5, 130.2, 129.5, 129.2, 128.2, 127.5, 126.1, 126.0, 40.4, 39.7, 33.2, 32.9, 31.9, 29.7, 28.8, 26.6, 23.0, 22.7, 14.1, 14.1.

HRMS(m/z) Found [M+H]+ = 875.6490, $C_{66}H_{82}$ requires 875.6495, Δ = -0.6 ppm

3,3''-bis((1E,3E,5E)-6-(4-(2-ethylhexyl)phenyl)hexa-1,3,5-trien-1-yl)-1,1':3',1''-terphenyl (*m*-(*m*DPH)$_2$)

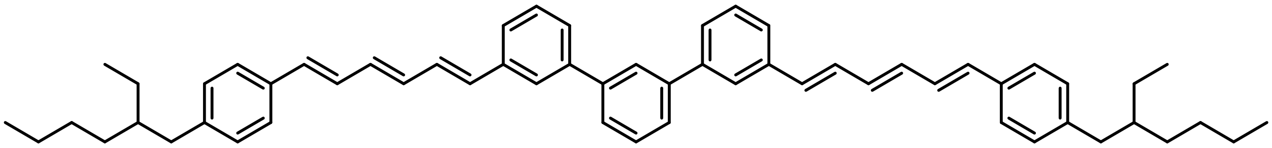

***m*-(*m*DPH)$_2$**

Dimer ***m*-(*m*DPH)$_2$** was synthesized by a Suzuki coupling of **Br- *m*DPH** and benzene-1,3-diboronic acid bispinacol ester.

A 20 mL microwave vial was charged with a stir bar, **Br- *m*DPH** (200 mg, 0.47 mmol, 2.1 eq.), benzene-1,3-diboronic acid bispinacol ester (73 mg, 0.22 mmol, 1.0 eq.) and $Pd(PPh_3)_4$ (25 mg, 0.022 mmol, 0.1 eq.) and flushed under argon. THF (7 mL) and an aqueous solution of Na_2CO_3 (2M, 0.7 mL) were separately degassed and then added to the solid reagents. The reaction mixture was lowered into a preheated oil bath and heated at 65 °C in the dark for 2 days. The reaction mixture was carefully acidified with 1 M HCl (~ 4 mL) with stirring and then diluted with DCM (~100 mL) and brine (~75 mL). The organic layer was separated and washed with brine (~ 30 mL), dried ($MgSO_4$) and the solvent remove *in vacuo*. The crude material was then purified by flash column chromatography (eluent: DCM/ *n*-hexane 2:8 v/v). Following removal of the solvent the columned material was triturated in hexanes and filtered to obtain the product. ***m*-(*m*DPH)$_2$**, was obtained as a pale yellow powder (62.1 mg, 0.087 mmol, 40 %).

^{1}H NMR (400 MHz, $CDCl_3$) δ 7.83 (s, 1H), 7.68 (s, 2H), 7.61 (m, 2H), 7.58 – 7.48 (m, 3H), 7.43 (m, 4H), 7.33 (d, J = 8.0 Hz, 4H), 7.11 (d, J = 8.0 Hz, 4H), 7.04 – 6.93 (m, 2H), 6.87 (m, 2H), 6.74 – 6.46 (m, 8H), 2.60 – 2.44 (m, 4H), 1.66 – 1.54 (m, 2H), 1.39 – 1.17 (m, 16H), 0.88 (m, 12H).

^{13}C NMR (101 MHz, $CDCl_3$) δ 141.9, 141.8, 141.7, 138.2, 134.9, 134.3, 133.1, 133.0, 132.2, 129.9, 129.7, 129.4, 129.3, 128.3, 126.6, 126.4, 126.3, 126.3, 125.5, 125.5, 41.2, 40.1, 32.5, 29.0, 25.6, 23.2, 14.3, 10.9.

HRMS(m/z) Found [M+H]+ = 763.5217, $C_{66}H_{82}$ requires 763.5243, Δ = -3.4 ppm

3,3''-bis((1E,3E,5E)-6-(4-(2-ethylhexyl)phenyl)hexa-1,3,5-trien-1-yl)-1,1':4',1''-terphenyl (*p*-(*m*DPH)$_2$)

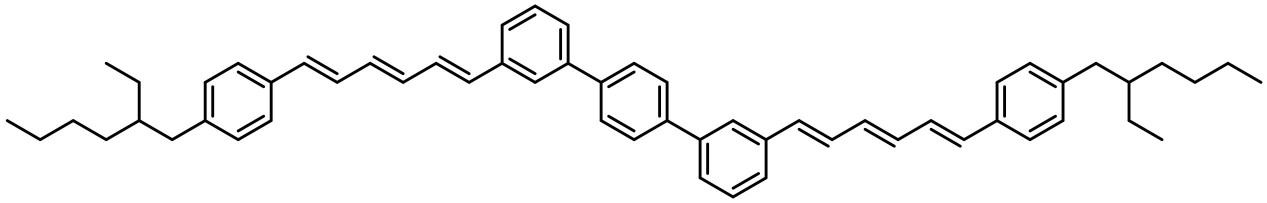

***p*-(*m*DPH)$_2$**

Dimer ***p*-(*m*DPH)$_2$** was synthesized by an analogous Suzuki coupling to ***m*-(*m*DPH)$_2$**, on the same scale, utilizing the isomeric starting material 1,4-diboronic acid bispinacol ester. The product was less soluble requiring modification of the work up procedure.

The reaction mixture was carefully acidified with 1 M HCl (~ 4 mL) with stirring and then filtered. The precipitate was washed with THF, 1 M HCl, water and methanol before being recrystallized from chloroform and ethanol mixture. The product, ***p*-(*m*DPH)$_2$**, was obtained as pale yellow powder (71.3 mg, 0.097 mmol, 44 %).

^{1}H NMR (500 MHz, $CDCl_3$) δ 7.69 (m, 6H), 7.50 (m, 2H), 7.42 (m, 4H), 7.34 (d, J = 8.0 Hz, 4H), 7.11 (d, J = 8.0 Hz, 4H), 7.02 – 6.93 (m, 2H), 6.90 – 6.83 (m, 2H), 6.68 – 6.51 (m, 8H), 2.53 – 2.47 (m, 4H), 1.34 – 1.20 (m, 16H), 1.00 – 0.75 (m, 12H).

^{13}C NMR (126 MHz, $CDCl_3$) δ 141.9, 141.3, 140.3, 138.2, 134.9, 134.3, 133.1, 132.3, 129.9, 129.7, 129.3, 128.3, 127.7, 126.4, 126.3, 125.4, 125.3, 41.2, 40.1, 32.5, 29.0, 25.6, 23.2, 14.3, 10.9.

HRMS(m/z) Found [M+H]+ = 763.5229, $C_{66}H_{82}$ requires 763.5243, Δ = -1.8 ppm

3,3''-bis((1E,3E,5E)-6-(4-(2-ethylhexyl)phenyl)hexa-1,3,5-trien-1-yl)-1,1':2',1''-terphenyl (*o*-(*m*DPH)$_2$)

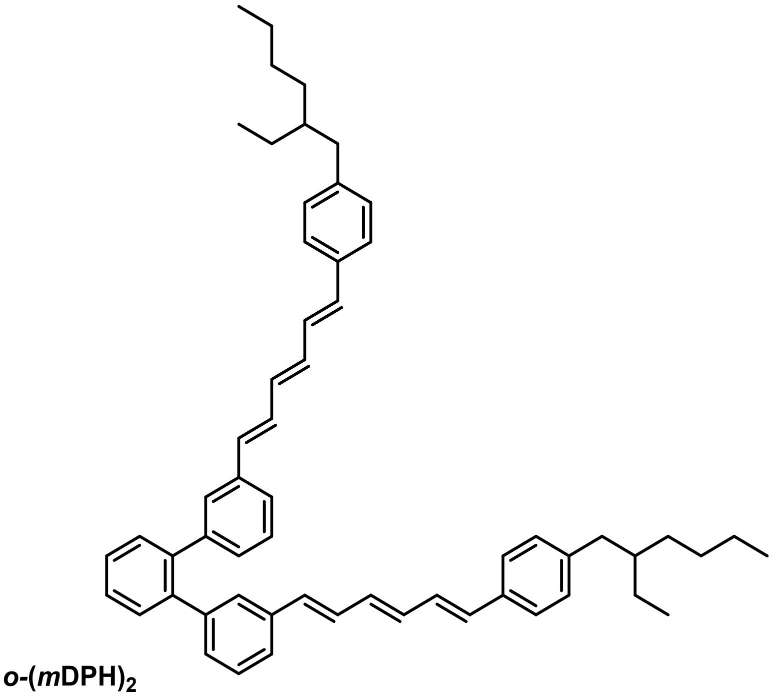

***o*-(*m*DPH)$_2$**

Dimer ***o*-(*m*DPH)$_2$** was synthesized by an analogous Suzuki coupling to ***m*-(*m*DPH)$_2$**, on the same scale, utilizing the isomeric starting material 1,2-diboronic acid bispinacol ester.

The product, ***o*-(*m*DPH)$_2$**, was obtained as pale yellow powder (68.2 mg, 0.093 mmol, 42 %).

^{1}H NMR (400 MHz, $CDCl_3$) δ 7.53 – 7.40 (m, 4H), 7.34 – 7.27 (m, 6H), 7.25 – 7.22 (m, 2H), 7.15 (t, *J* = 7.6 Hz, 2H), 7.06 (d, *J* = 8.1 Hz, 4H) 7.00 – 6.96 (m, 2H), 6.88 – 6.81 (m, 2H), 6.74 – 6.64 (m, 2H), 6.59 – 6.40 (m, 8H), 2.55 – 2.46 (m, 4H), 1.34 – 1.18 (m, 16H), 0.92 – 0.83 (m, 12H).

^{13}C NMR (101 MHz, $CDCl_3$) δ 141.9, 141.7, 140.6, 137.3, 134.9, 134.2, 133.1, 133.1, 132.3, 130.5, 129.6, 129.2, 128.3, 128.3, 128.1, 127.8, 126.4, 124.9, 41.2, 40.0, 32.5, 29.0, 25.6, 23.2, 14.3, 10.9.

HRMS(m/z) Found [M+H]+ = 763.5217, $C_{66}H_{82}$ requires 763.5243, Δ = -3.4 ppm

7. NMR Spectra

1-bromo-4-(2-butyloctyl)benzene (2)

^{1}H NMR, 700 MHz, $CDCl_3$

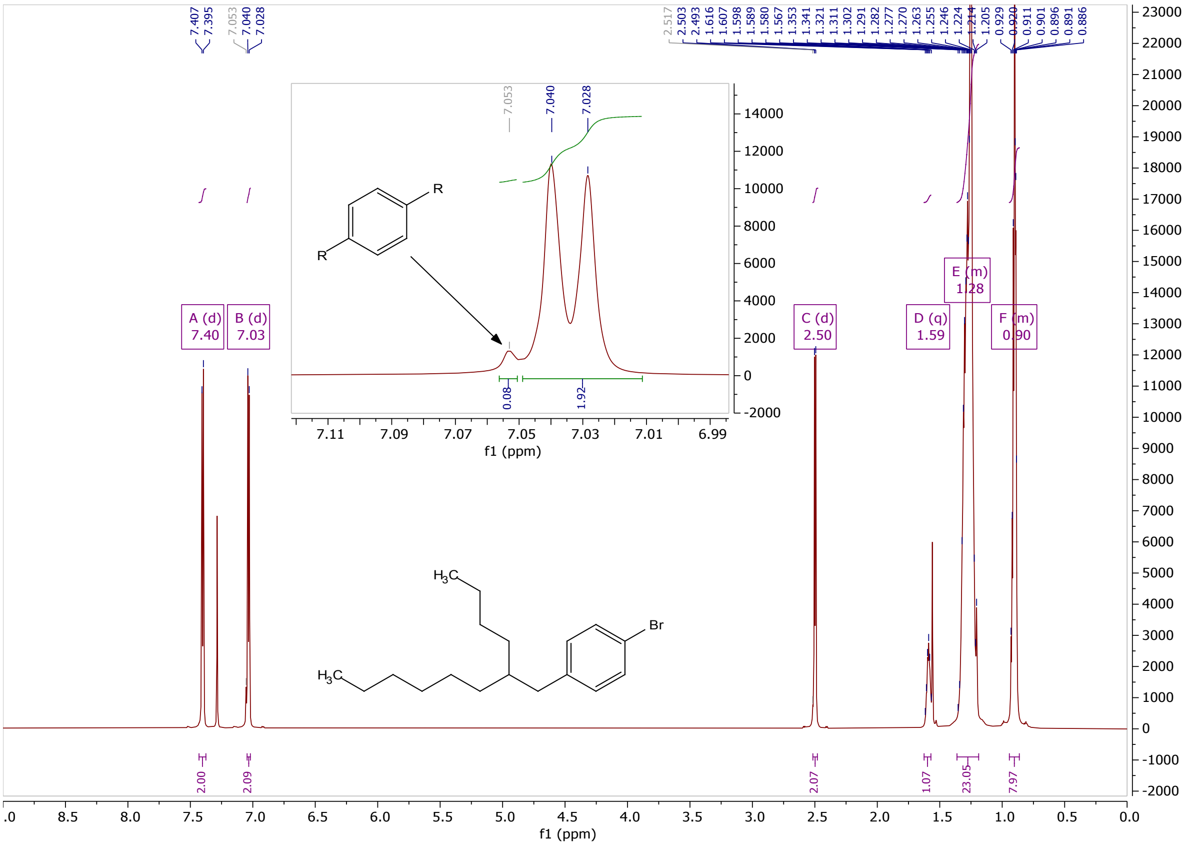

^{13}C NMR, 176 MHz, $CDCl_3$

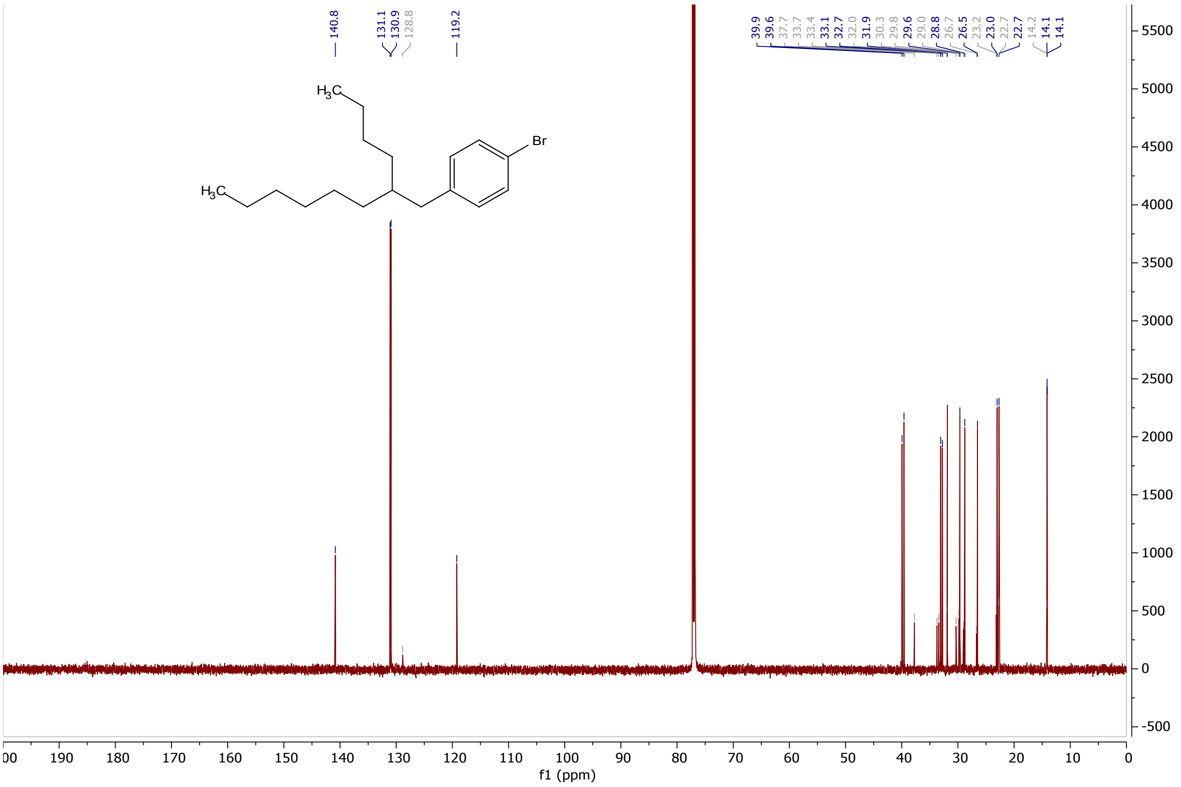

1-bromo-4-(2-ethylhexyl)benzene (2b)

^{1}H NMR, 700 MHz, $CDCl_3$

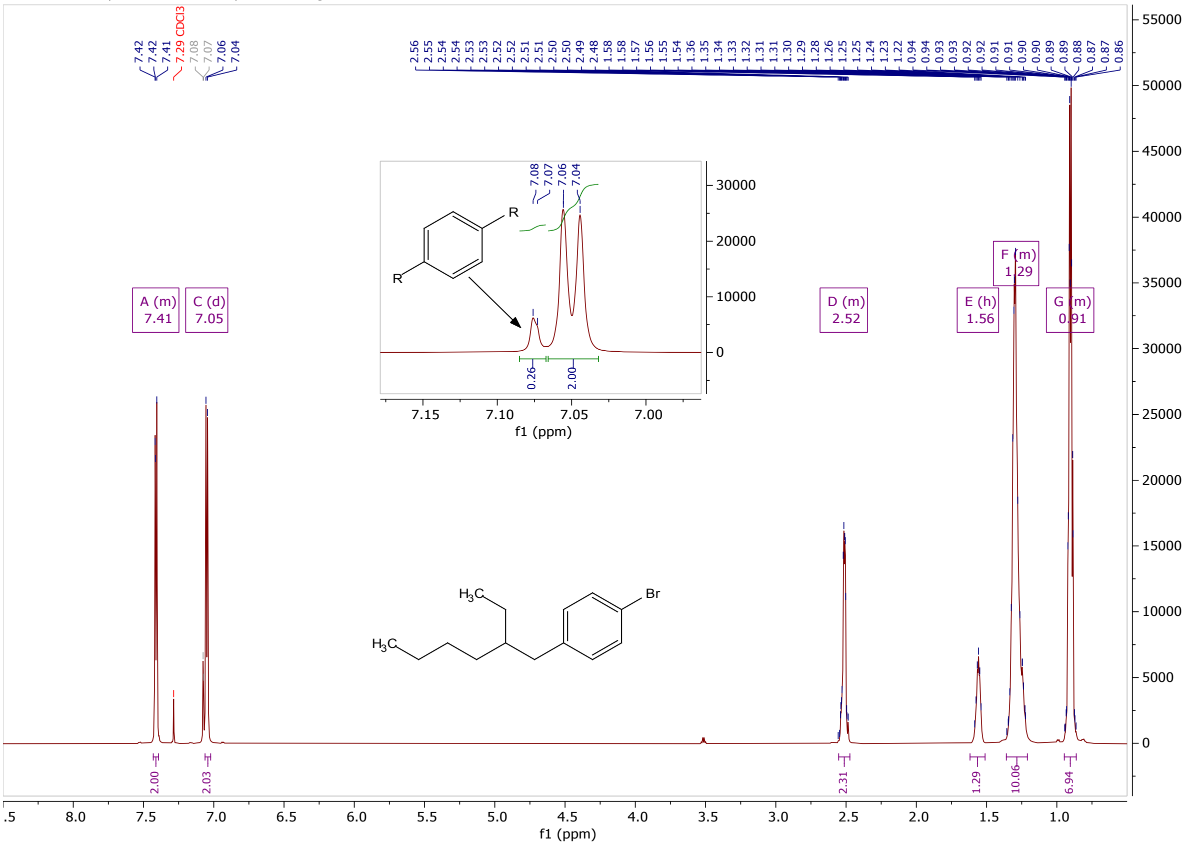

^{13}C NMR, 176 MHz, $CDCl_3$

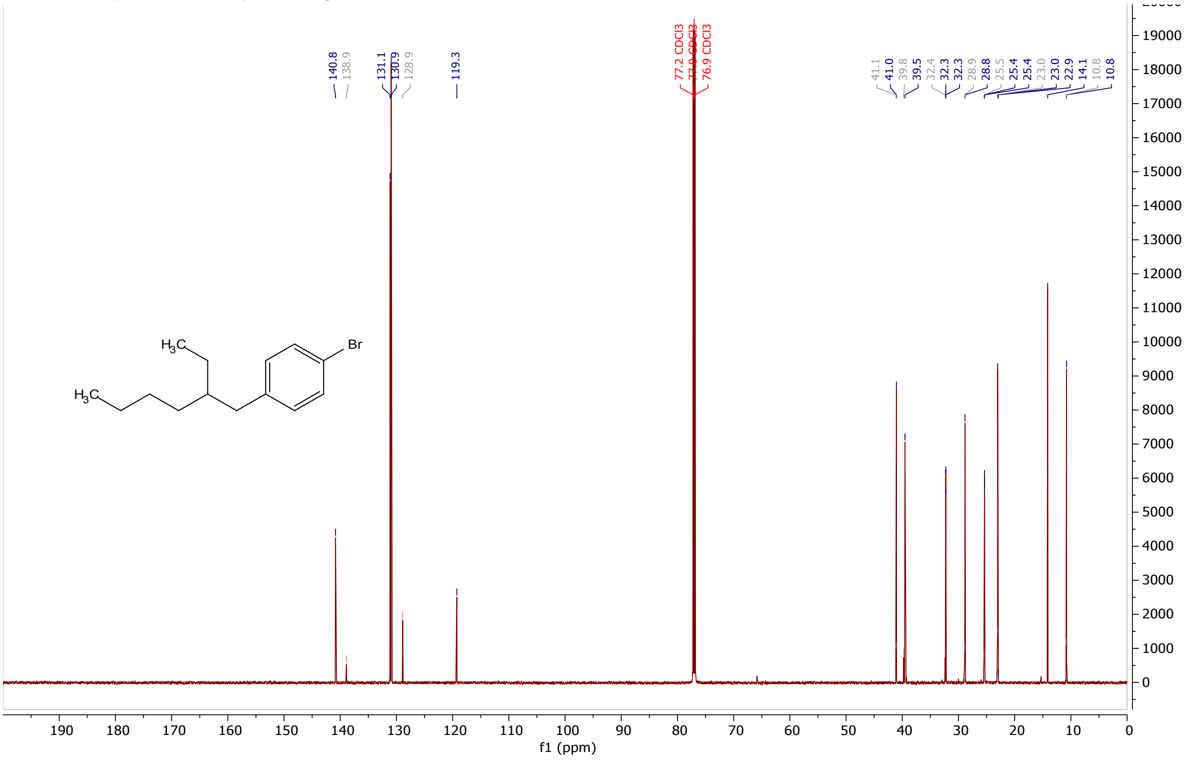

4-(2-butyloctyl)benzaldehyde (3)

^{1}H NMR, 400 MHz, $CDCl_3$

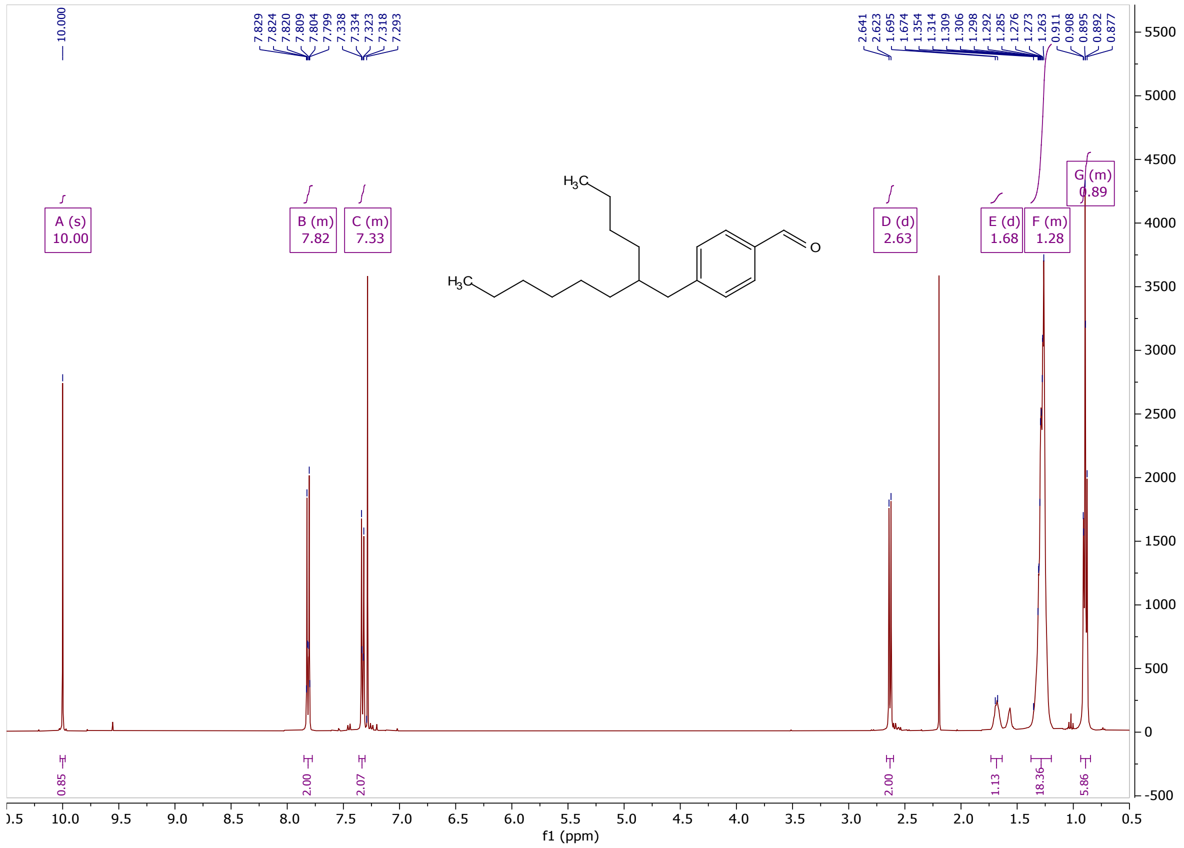

^{13}C NMR, 176 MHz, $CDCl_3$

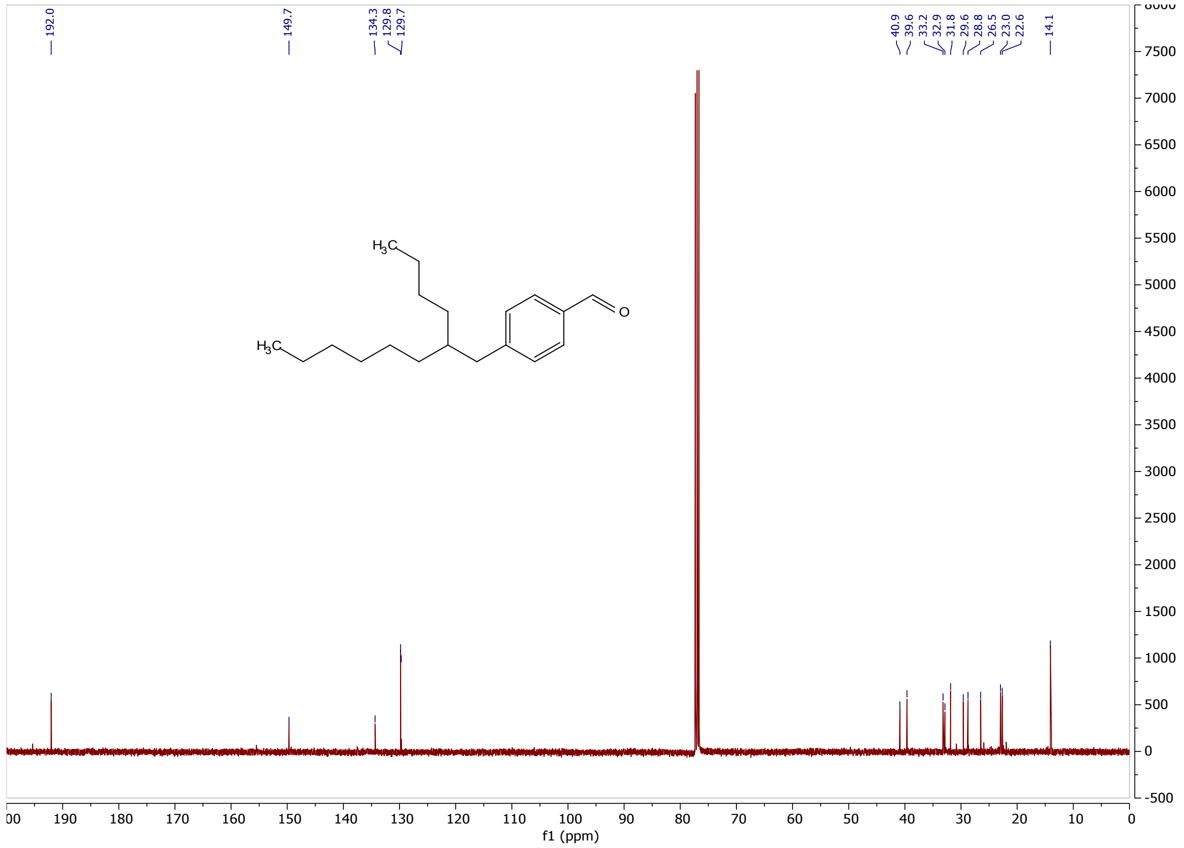

4-(2-butyloctyl)benzaldehyde **(3b)**

^{1}H NMR, 700 MHz, $CDCl_3$

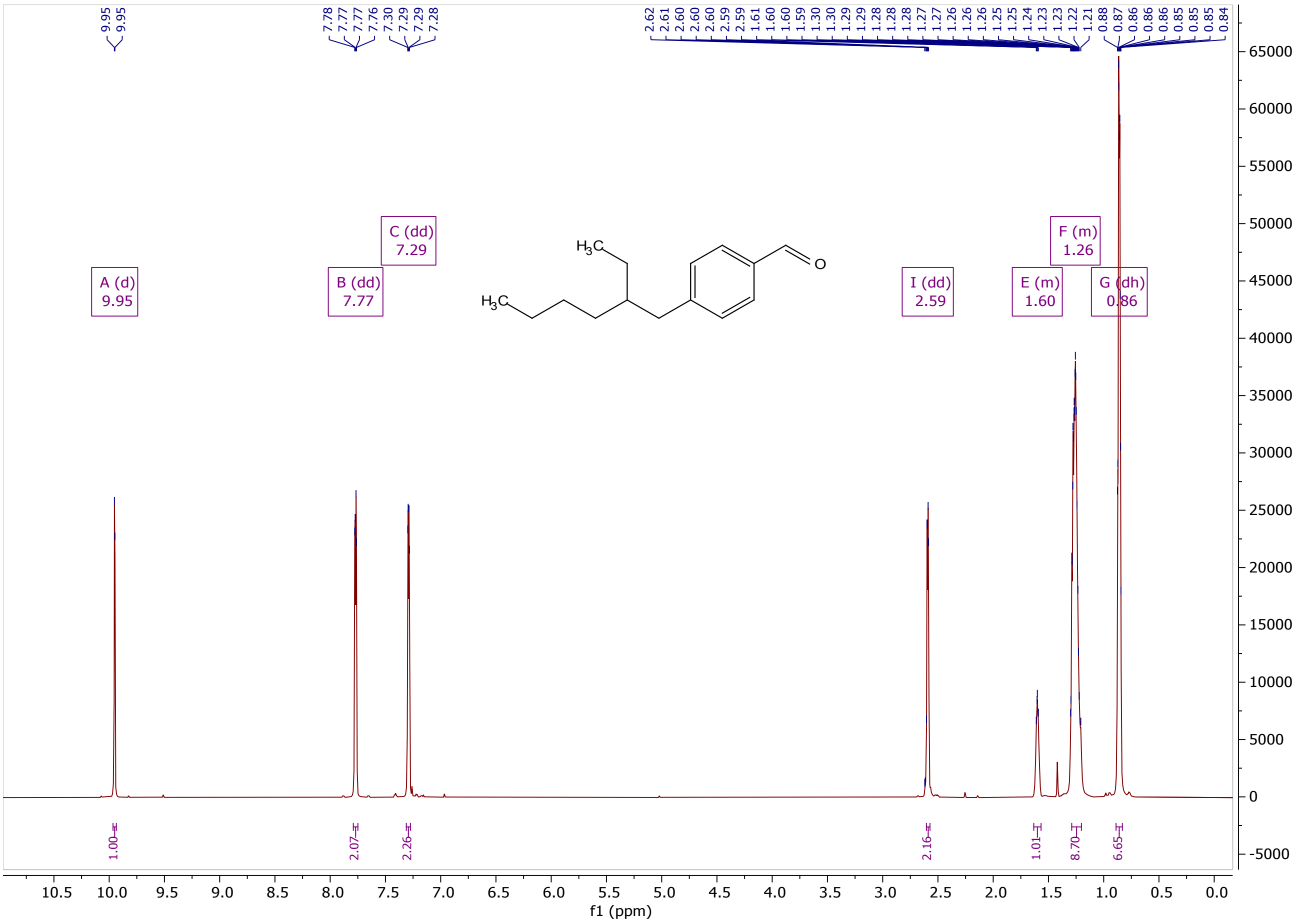

^{13}C NMR, 176 MHz, $CDCl_3$

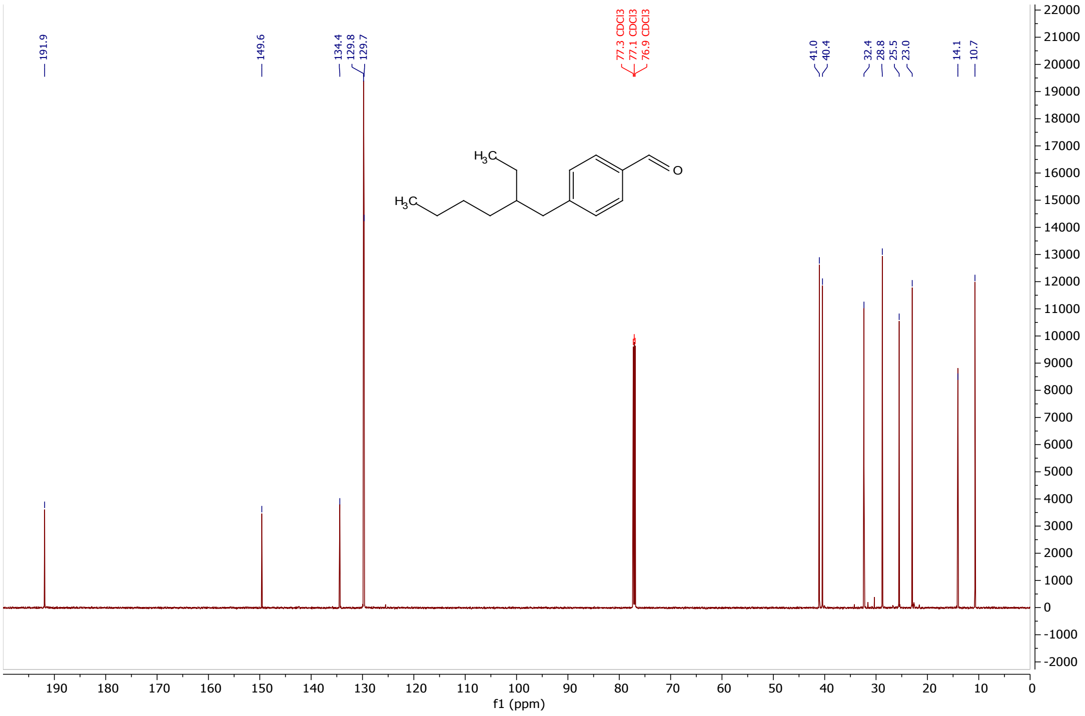

(E)-3-(4-(2-butyloctyl)phenyl)acrylaldehyde (4)

^{1}H NMR, 700 MHz, $CDCl_3$

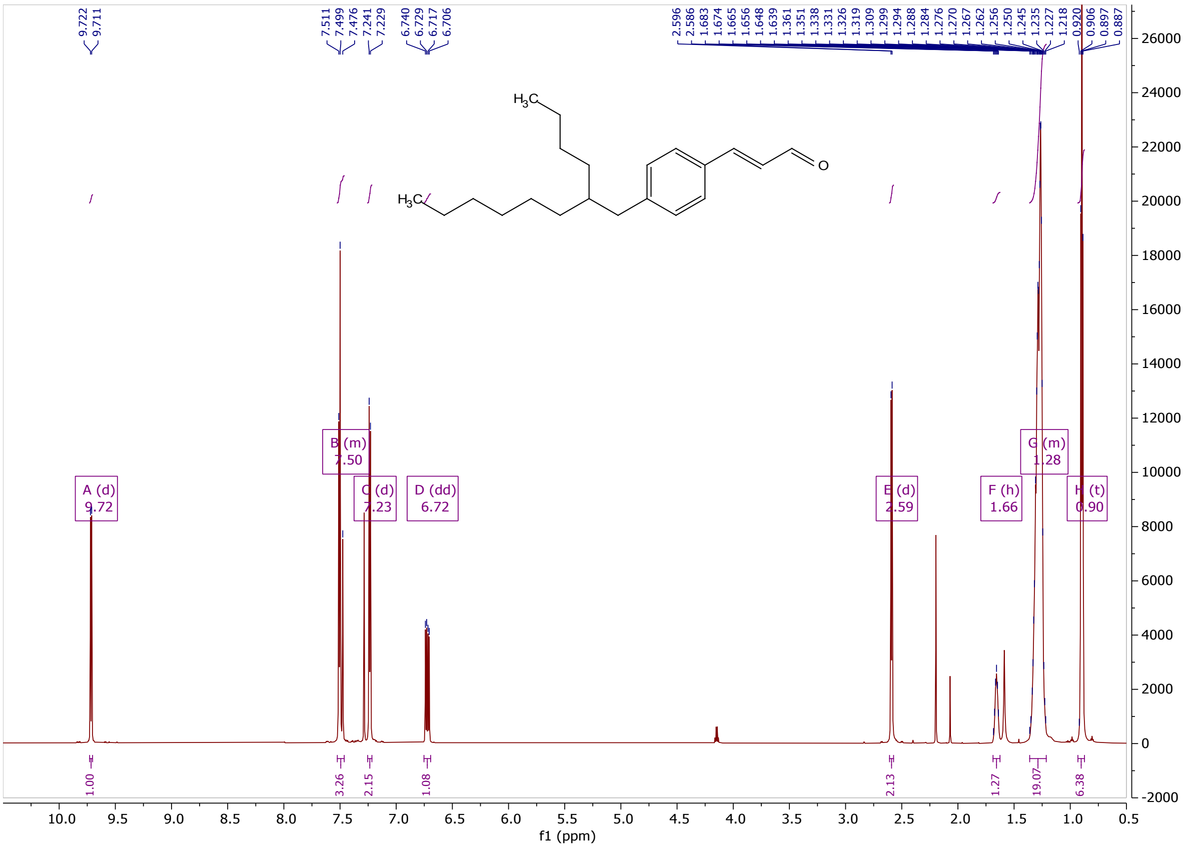

^{13}C NMR, 176 MHz, $CDCl_3$

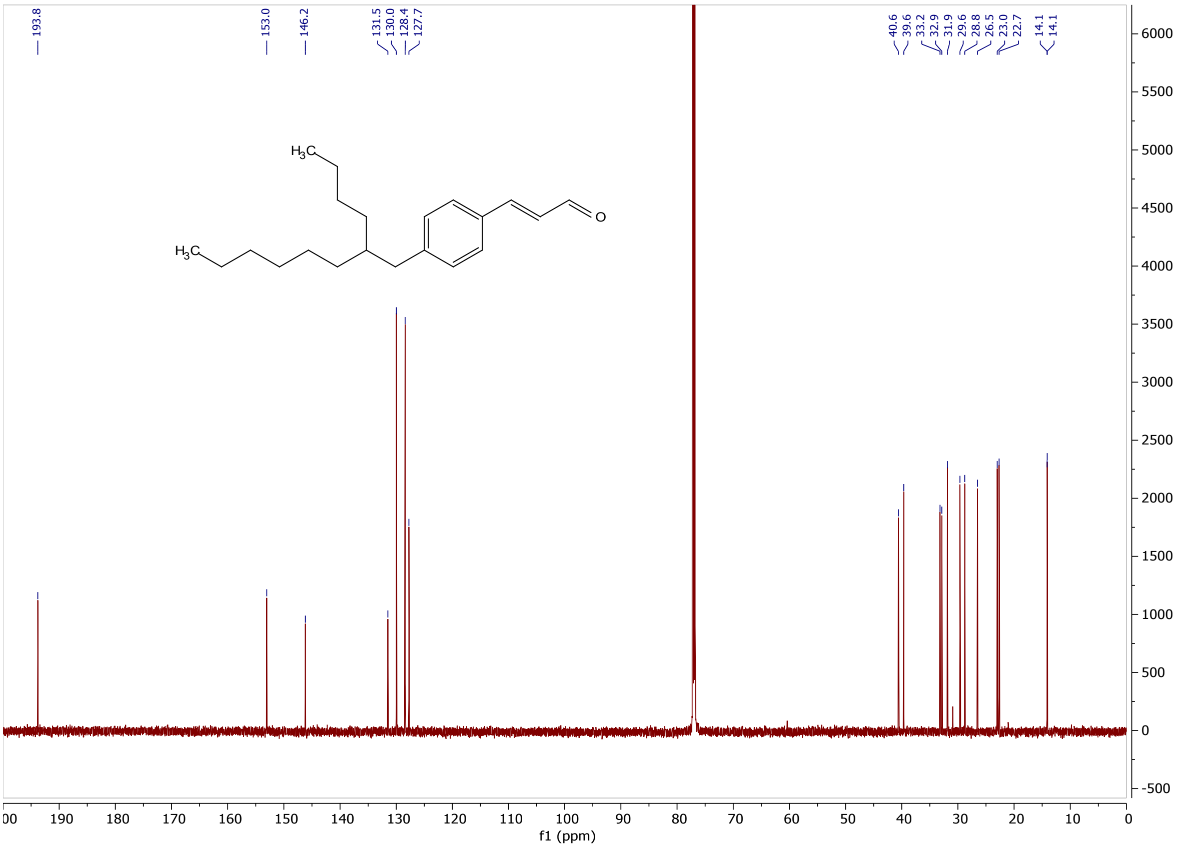

(E)-3-(4-(2-ethylhexyl)phenyl)acrylaldehyde (4b)

^{1}H NMR, 700 MHz, $CDCl_3$

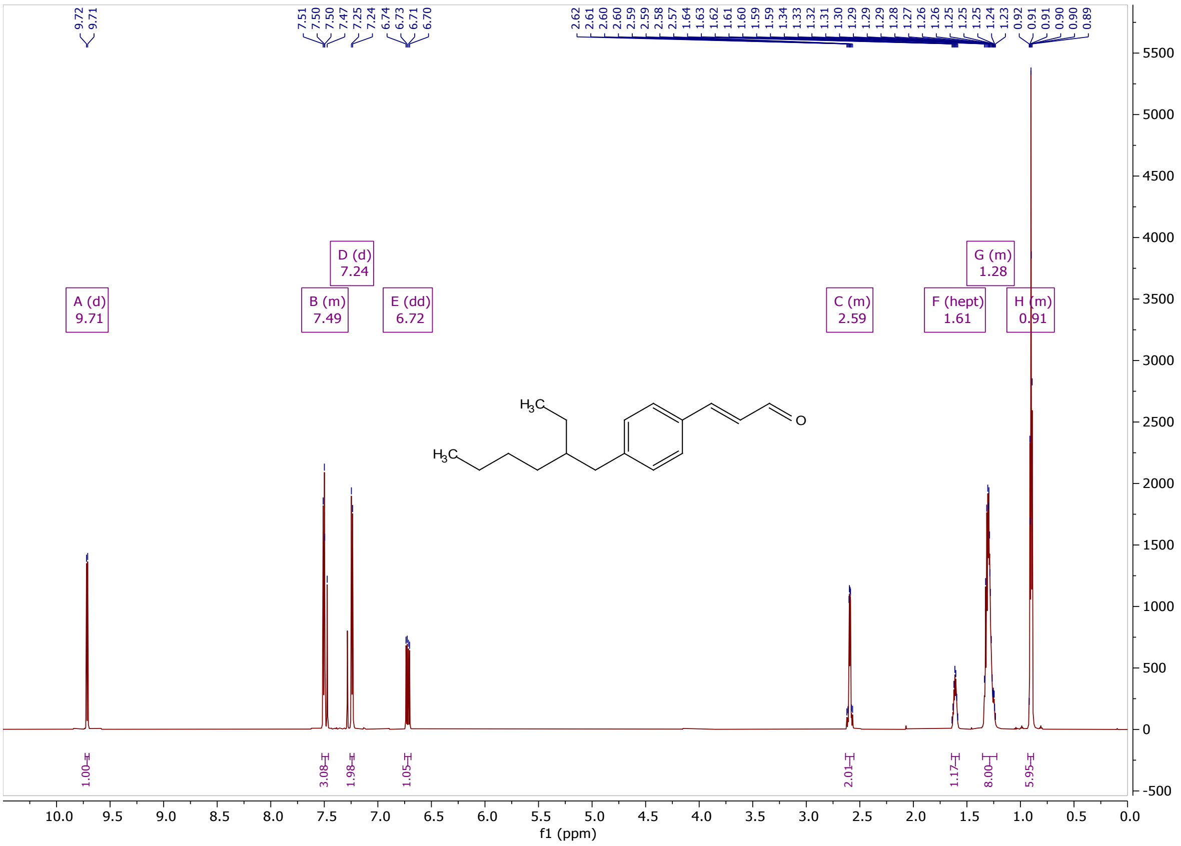

^{13}C NMR, 176 MHz, $CDCl_3$

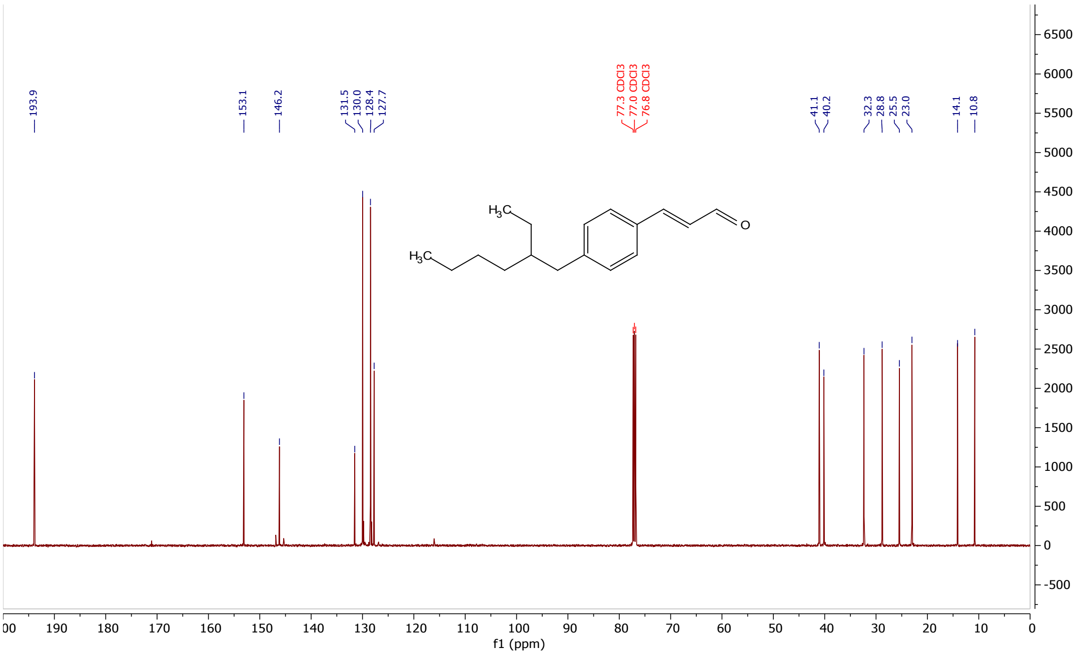

(2E,4E)-5-(4-(2-butyloctyl)phenyl)penta-2,4-dienal (5)

^{1}H NMR, 700 MHz, $CDCl_3$

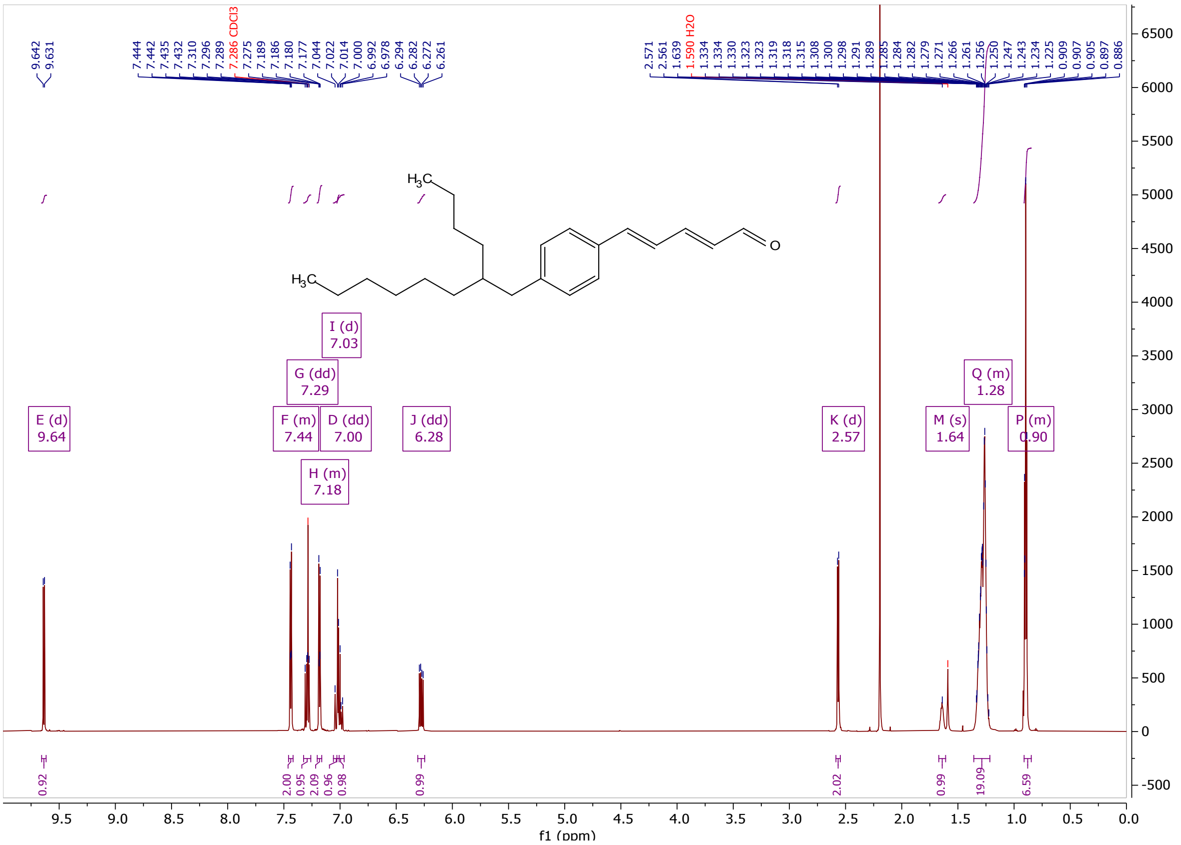

^{13}C NMR, 176 MHz, $CDCl_3$

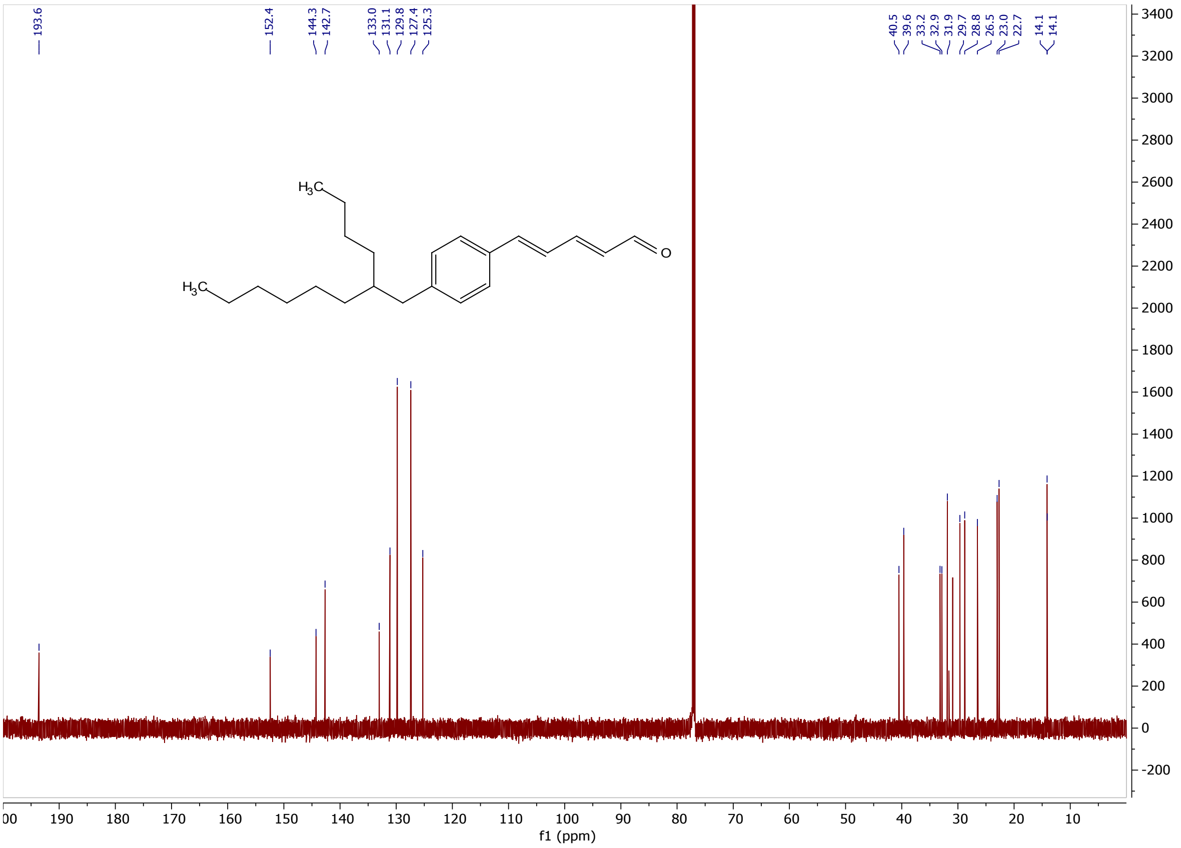

(2E,4E)-5-(4-(2-ethylhexyl)phenyl)penta-2,4-dienal (5b)

^{1}H NMR, 700 MHz, $CDCl_3$

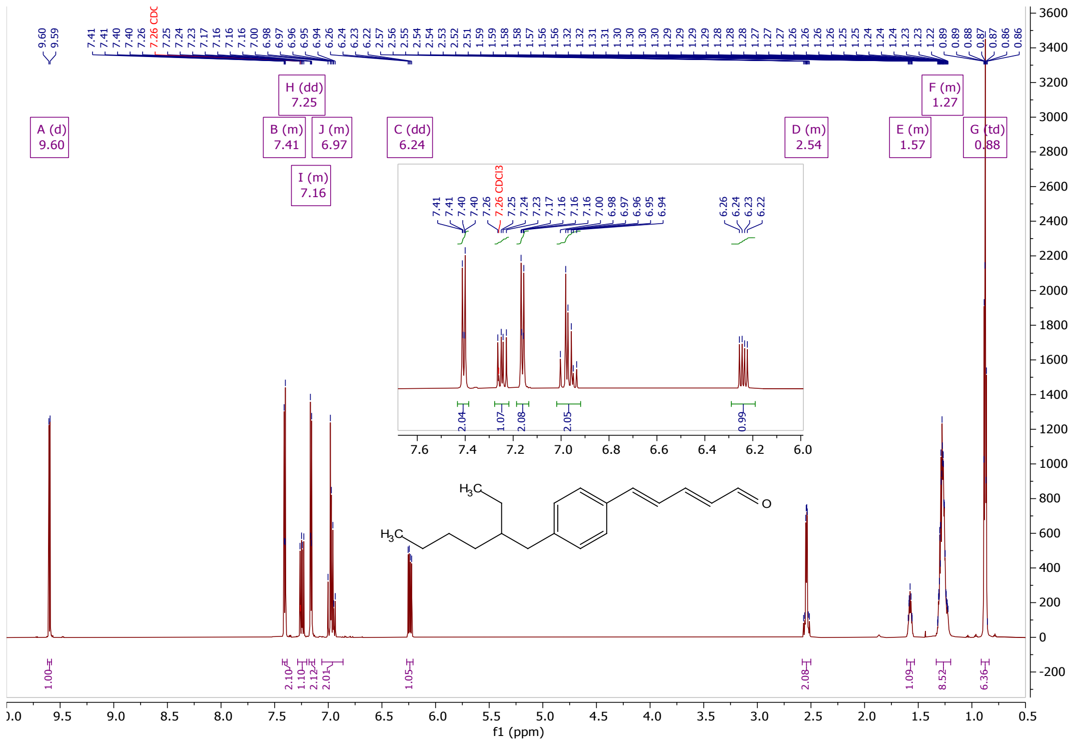

^{13}C NMR, 176 MHz, $CDCl_3$

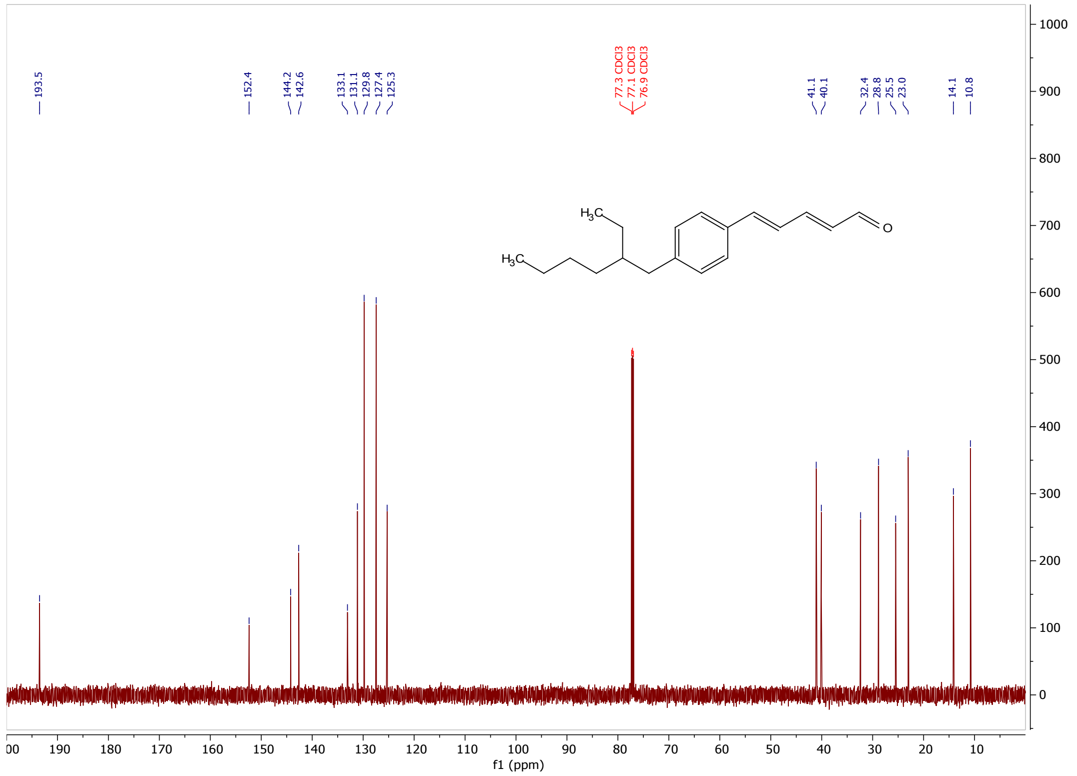

4-bromobenzyl triphenylphosphonium bromide (7)

^1H NMR, 400 MHz, $CDCl_3$

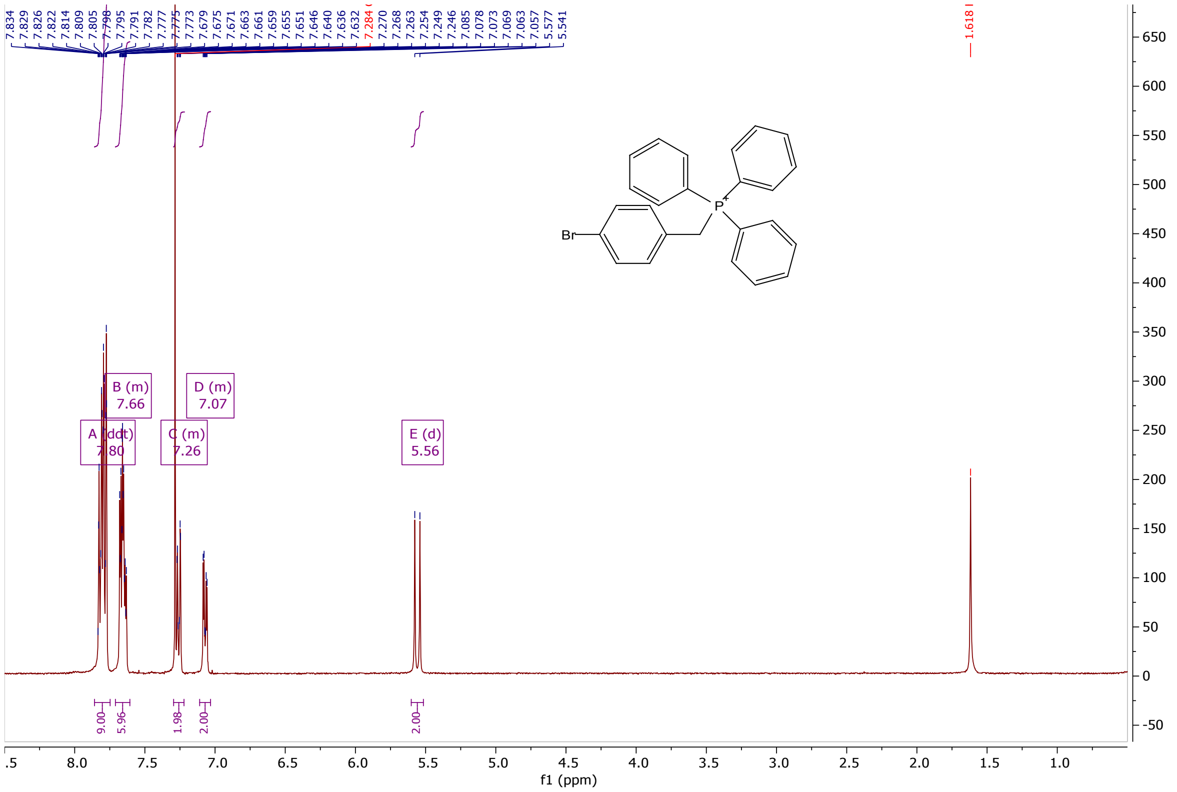

3-bromobenzyl triphenylphosphonium bromide (7b)

^{1}H NMR, 400 MHz, $CDCl_3$

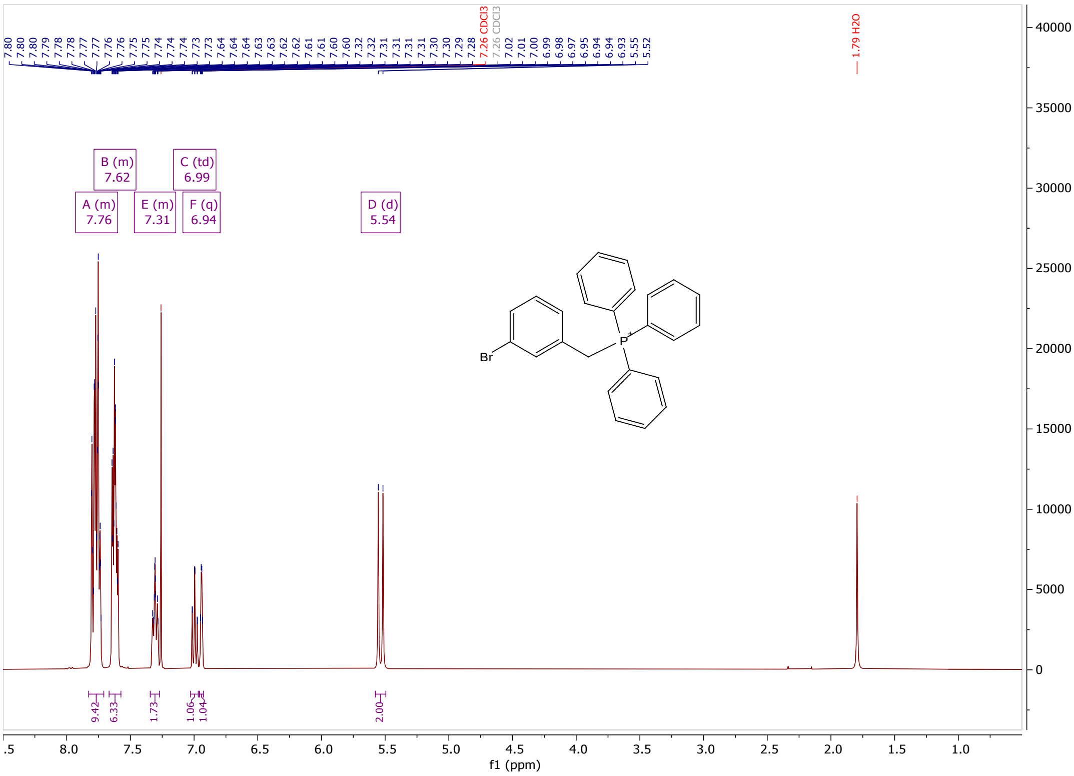

^{13}C NMR, 101 MHz, $CDCl_3$

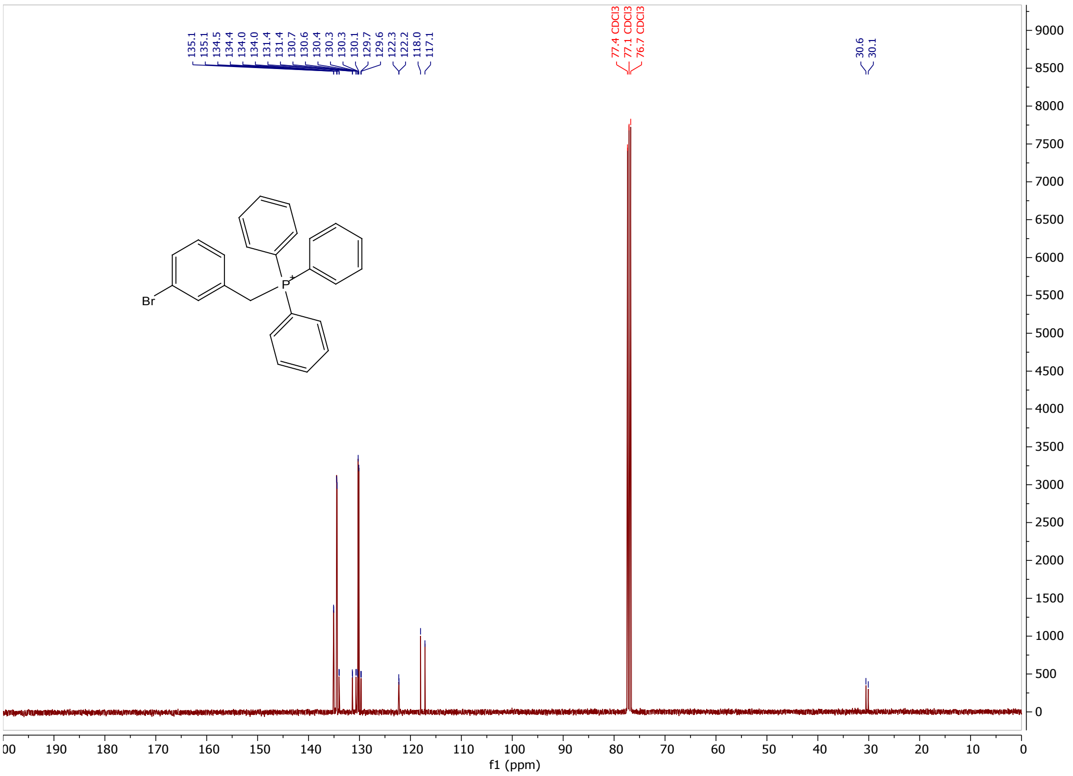

1-bromo-4-((1E,3E,5E)-6-(4-(2-butyloctyl)phenyl)hexa-1,3,5-trien-1-yl)benzene (Br- *p*DPH)

^{1}H NMR, 700 MHz, $CDCl_3$

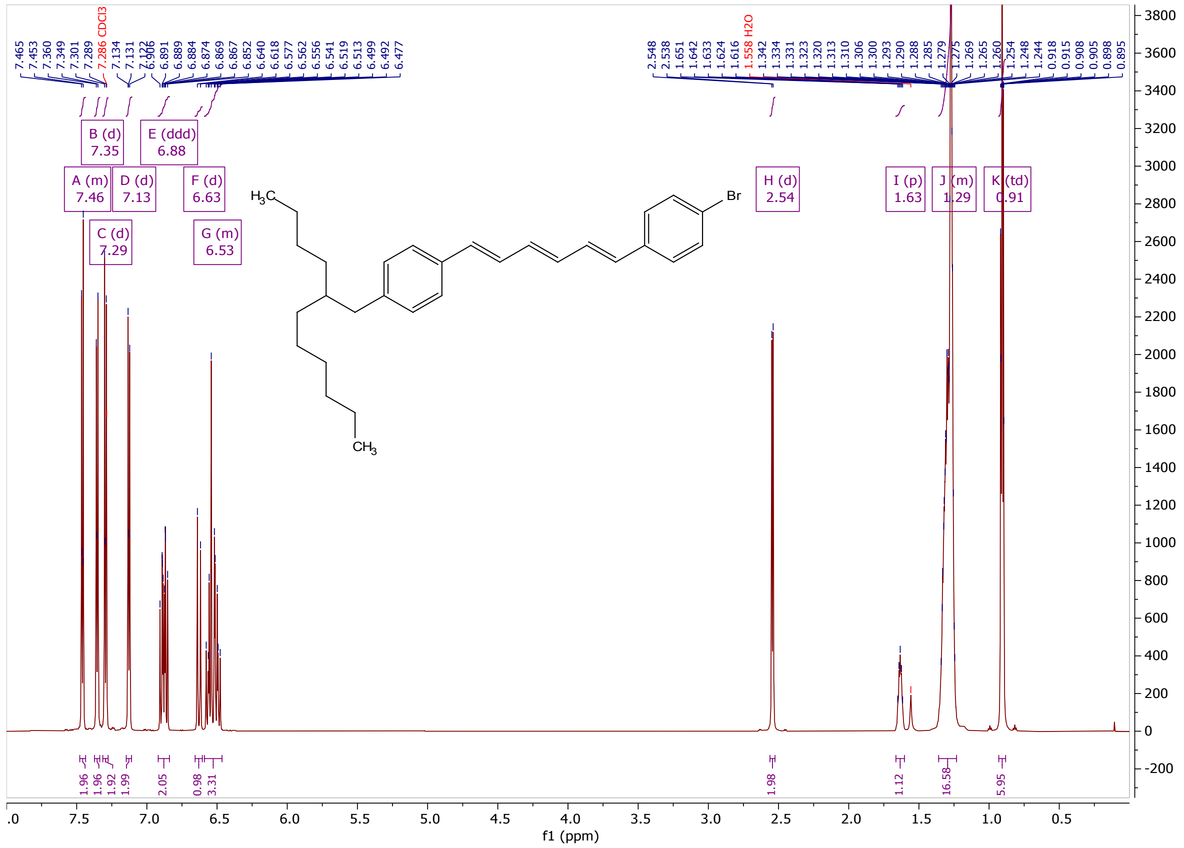

^{13}C NMR, 176 MHz, $CDCl_3$

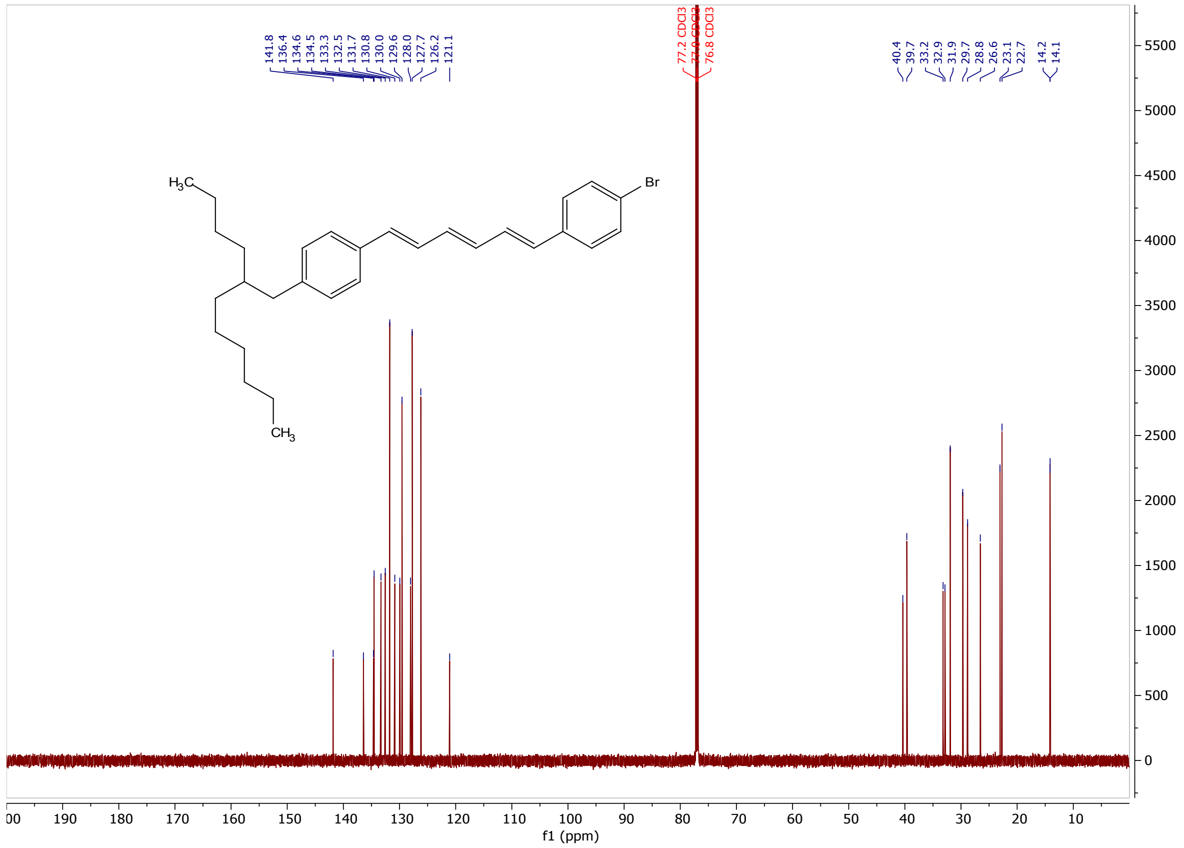

1-bromo-3-((1E,3E,5E)-6-(4-(2-ethylhexyl)phenyl)hexa-1,3,5-trien-1-yl)benzene (Br- *m*DPH)

^{1}H NMR, 400 MHz, $CDCl_3$

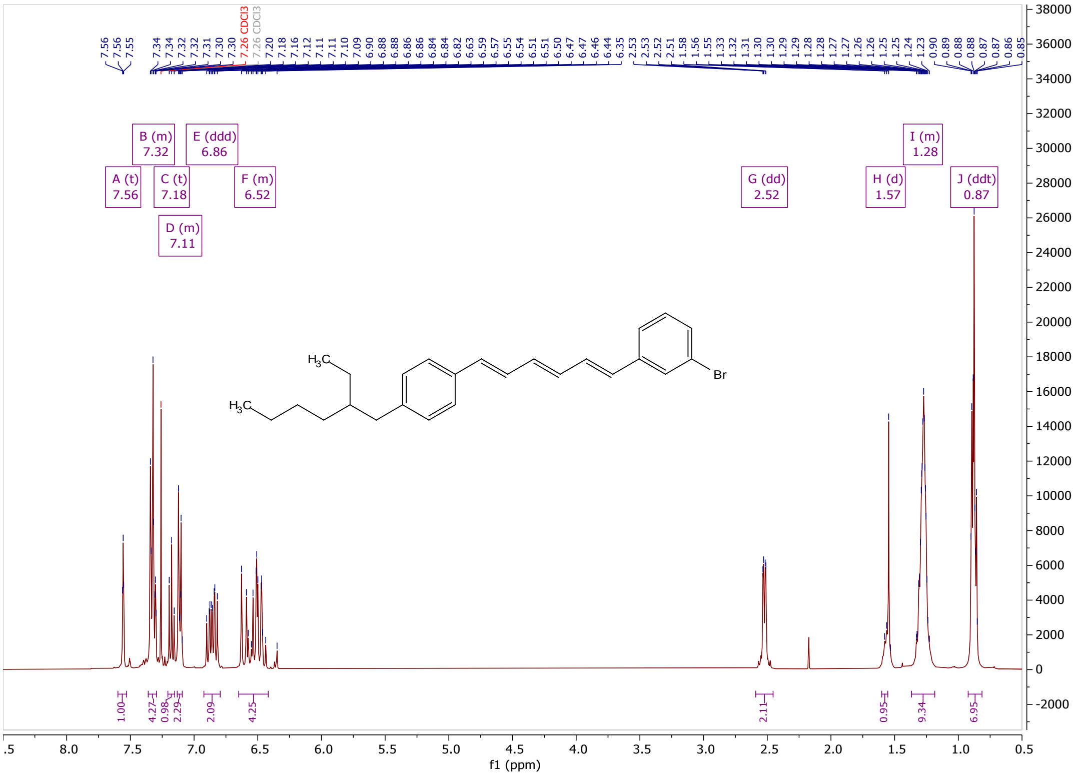

^{13}C NMR, 101 MHz, $CDCl_3$

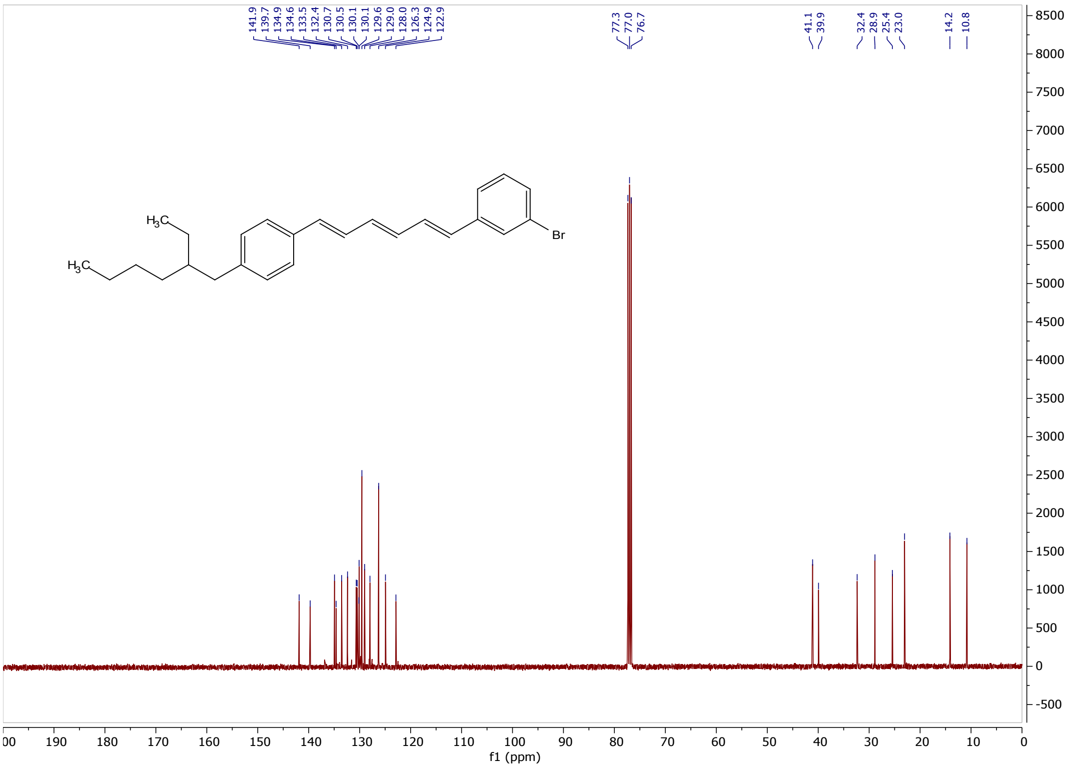

4-((1E,3E,5E)-6-(4-(2-butyloctyl)phenyl)hexa-1,3,5-trien-1-yl)-4'-methyl-1,1'-biphenyl (*p*Tol-*p*DPH)

^{1}H NMR, 700 MHz, $CDCl_3$

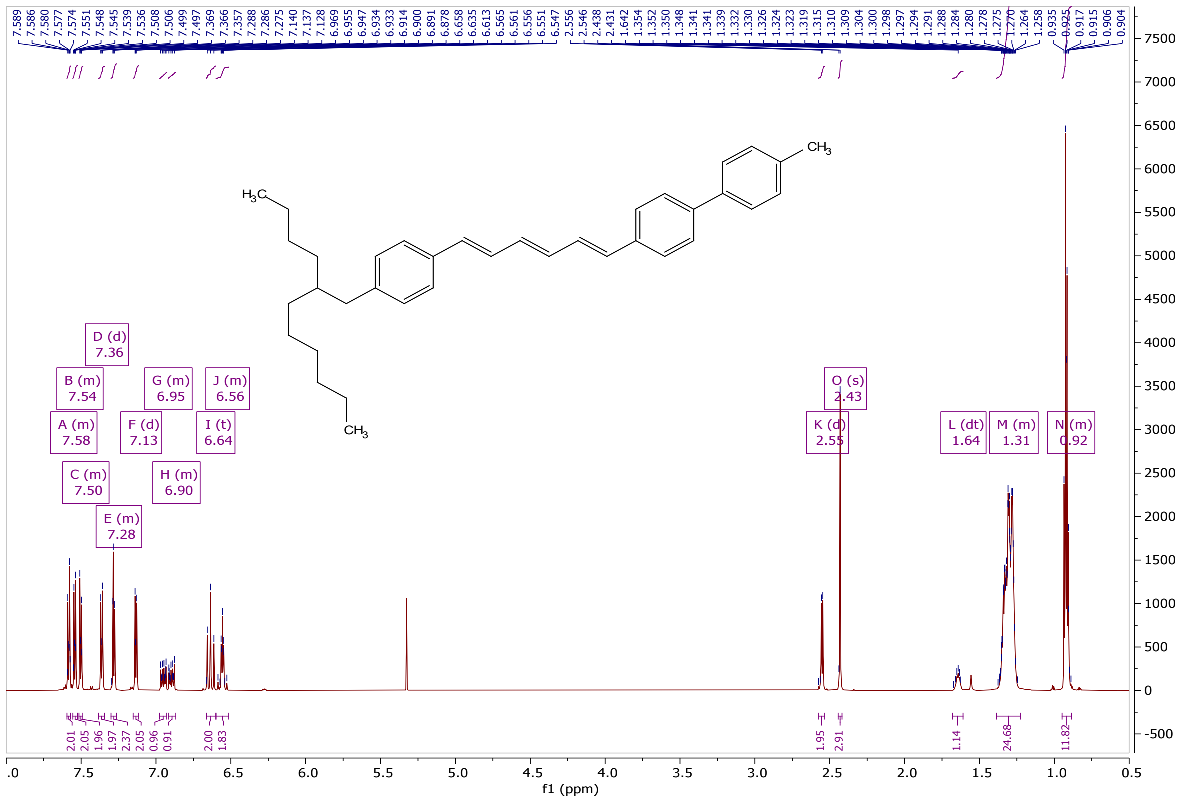

^{13}C NMR, 176 MHz, $CDCl_3$

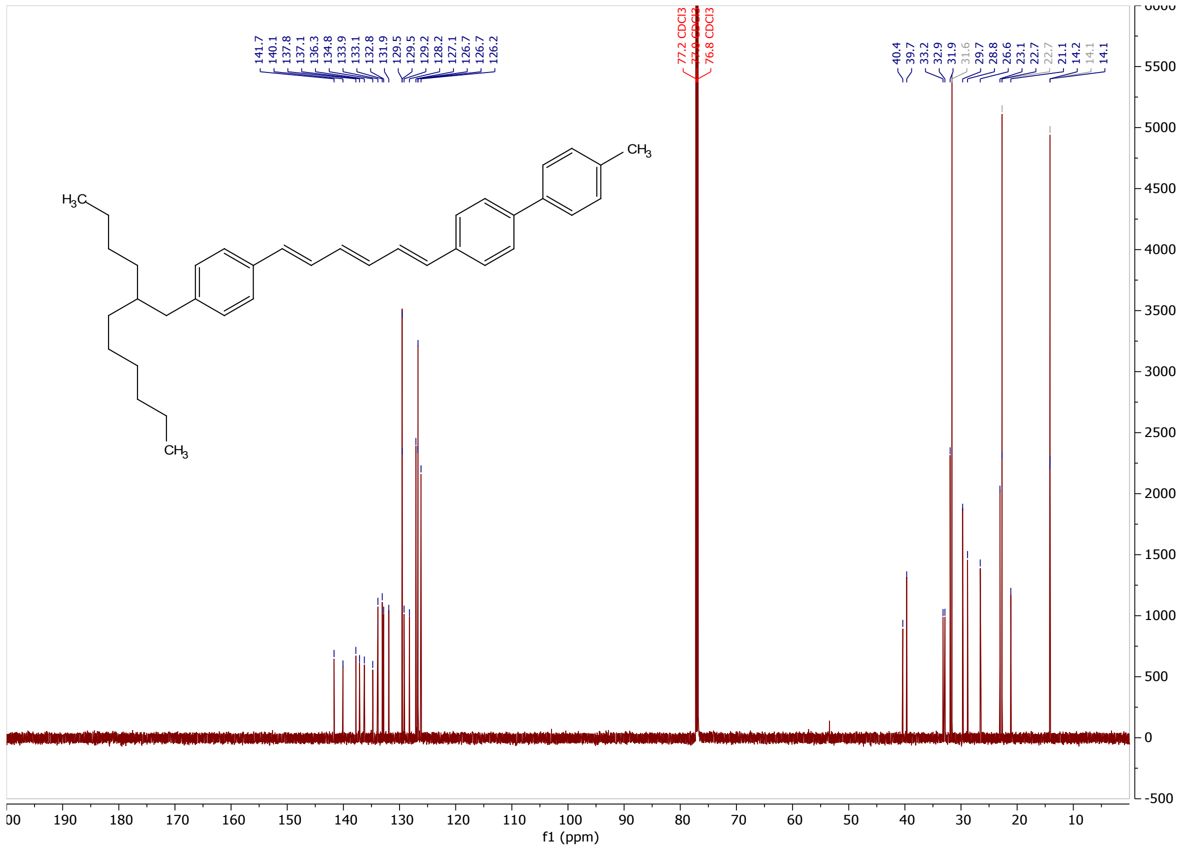

3-((1E,3E,5E)-6-(4-(2-ethylhexyl)phenyl)hexa-1,3,5-trien-1-yl)-4'-methyl-1,1'-biphenyl (*p*Tol-*m*DPH)

^{1}H NMR, 500 MHz, $CDCl_3$

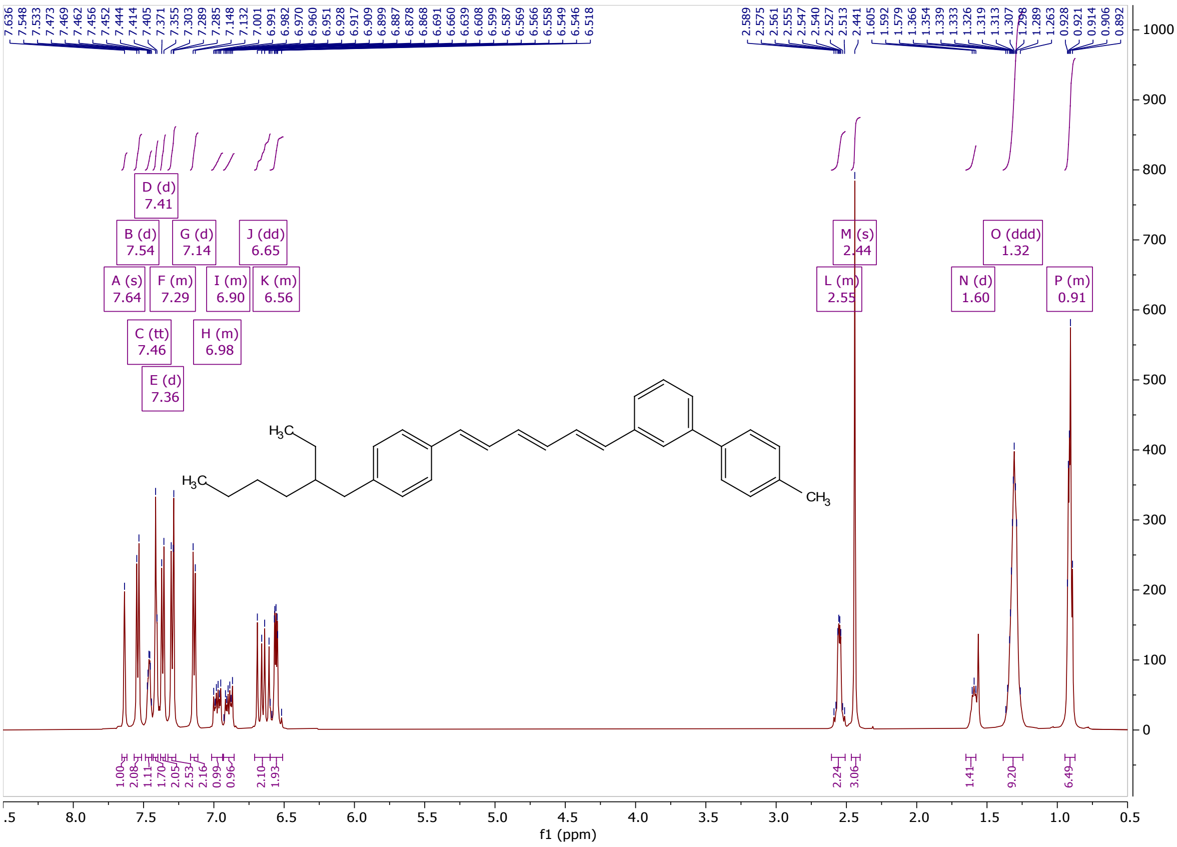

^{13}C NMR, 126 MHz, $CDCl_3$

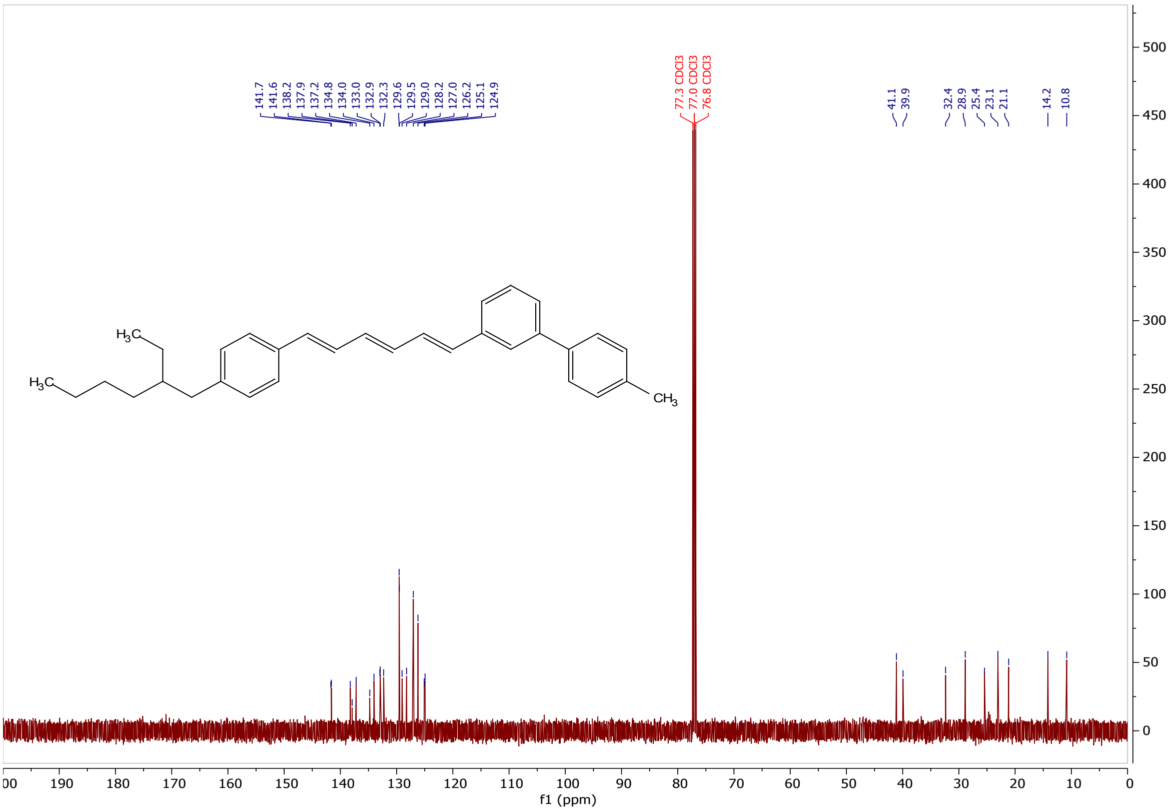

4,4''-bis((1E,3E,5E)-6-(4-(2-butyloctyl)phenyl)hexa-1,3,5-trien-1-yl)-1,1':4',1''-terphenyl (*p*-(*p*DPH)$_2$)

^{1}H NMR, 500 MHz, d_2-TCE, 70 °C

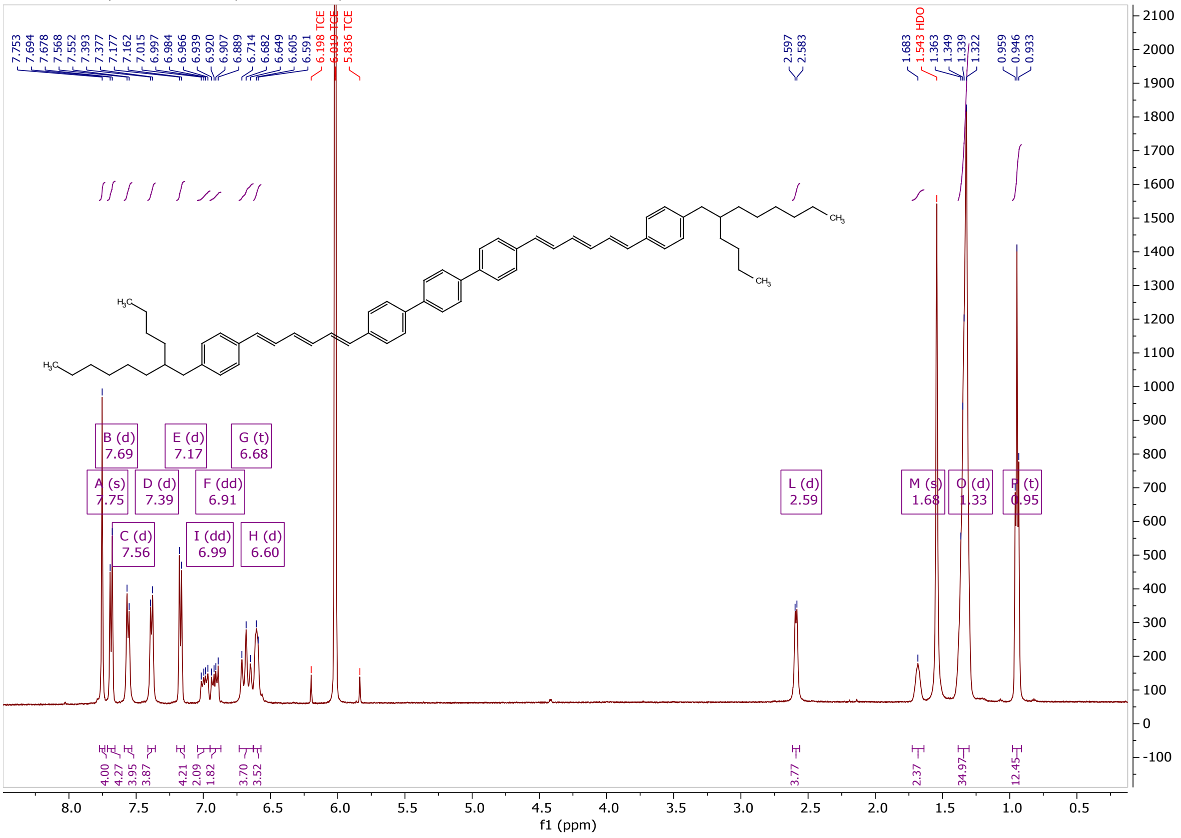

Variable temperature 500 MHz ^{1}H NMR spectra of *p*-(*p*DPH)$_2$ (6 mg in 0.4 ml d$_4$-TCE).

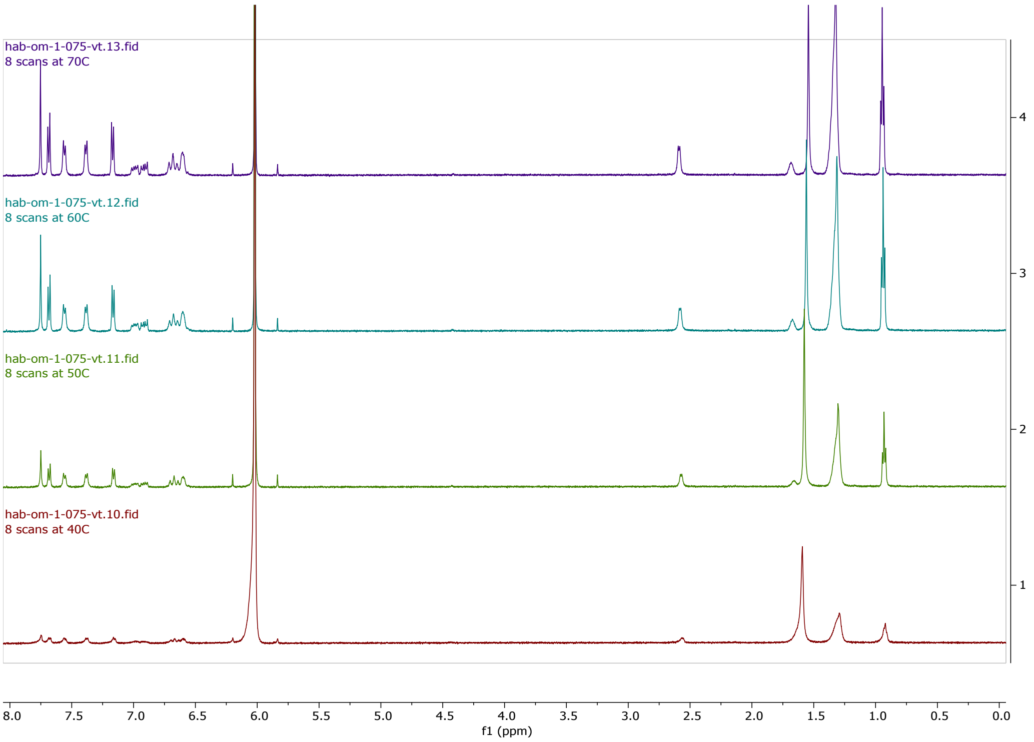

4,4''-bis((1E,3E,5E)-6-(4-(2-butyloctyl)phenyl)hexa-1,3,5-trien-1-yl)-1,1':3',1''-terphenyl (*m*-(*p*DPH)$_2$)

^{1}H NMR, 700 MHz, $CDCl_3$

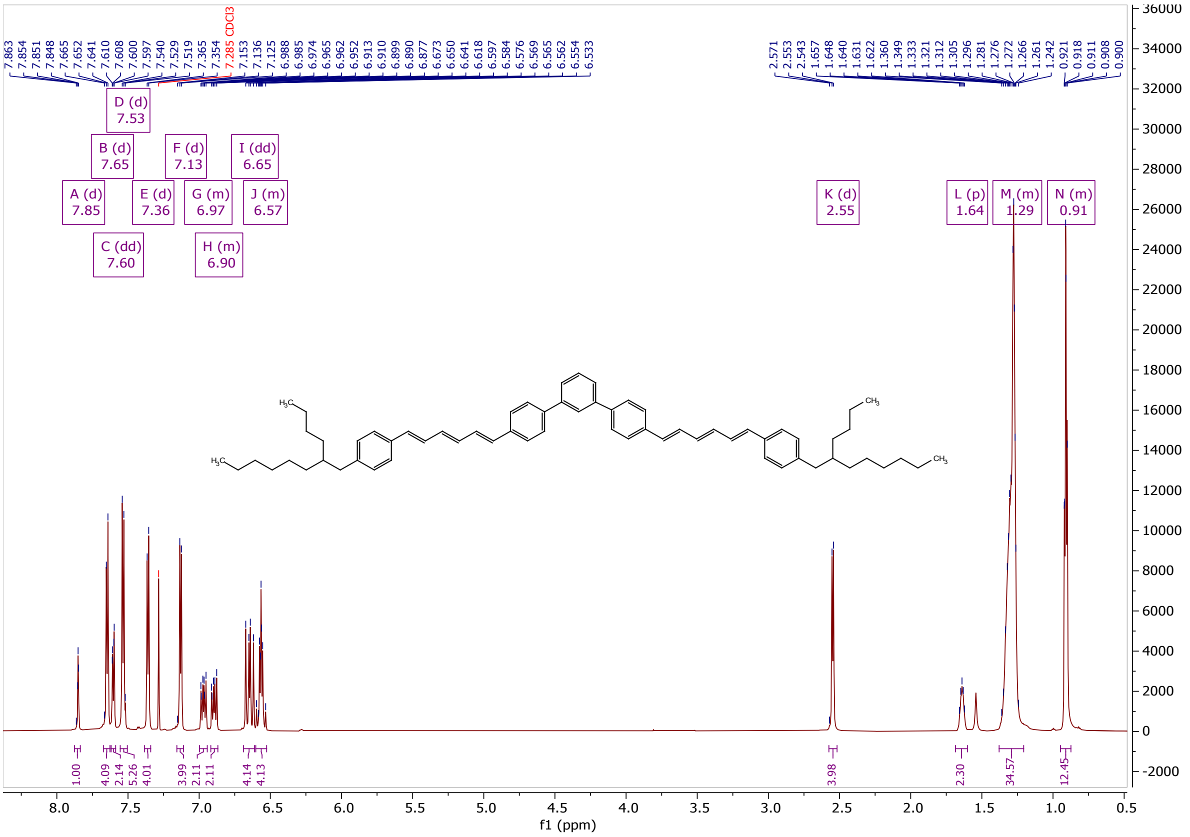

^{13}C NMR, 176 MHz, $CDCl_3$

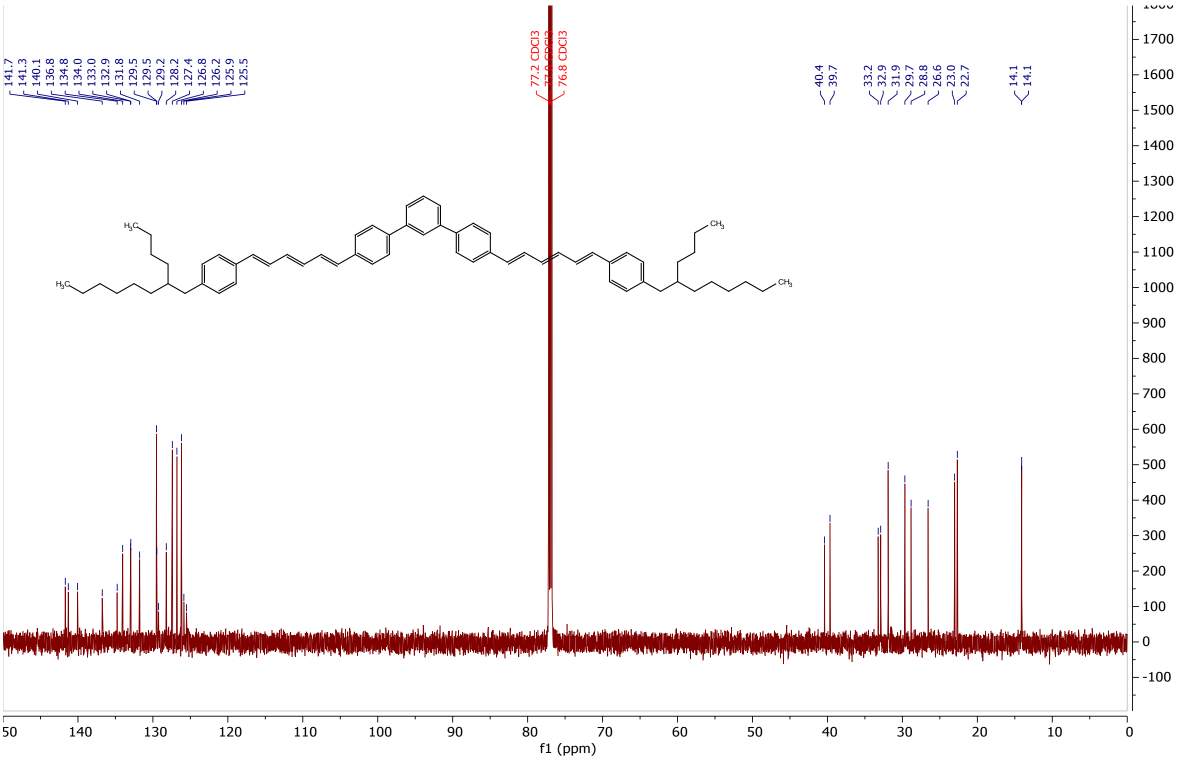

4,4''-bis((1E,3E,5E)-6-(4-(2-butyloctyl)phenyl)hexa-1,3,5-trien-1-yl)-1,1':2',1''-terphenyl (*o*-(*p*DPH)$_2$)

^{1}H NMR, 700 MHz, $CDCl_3$

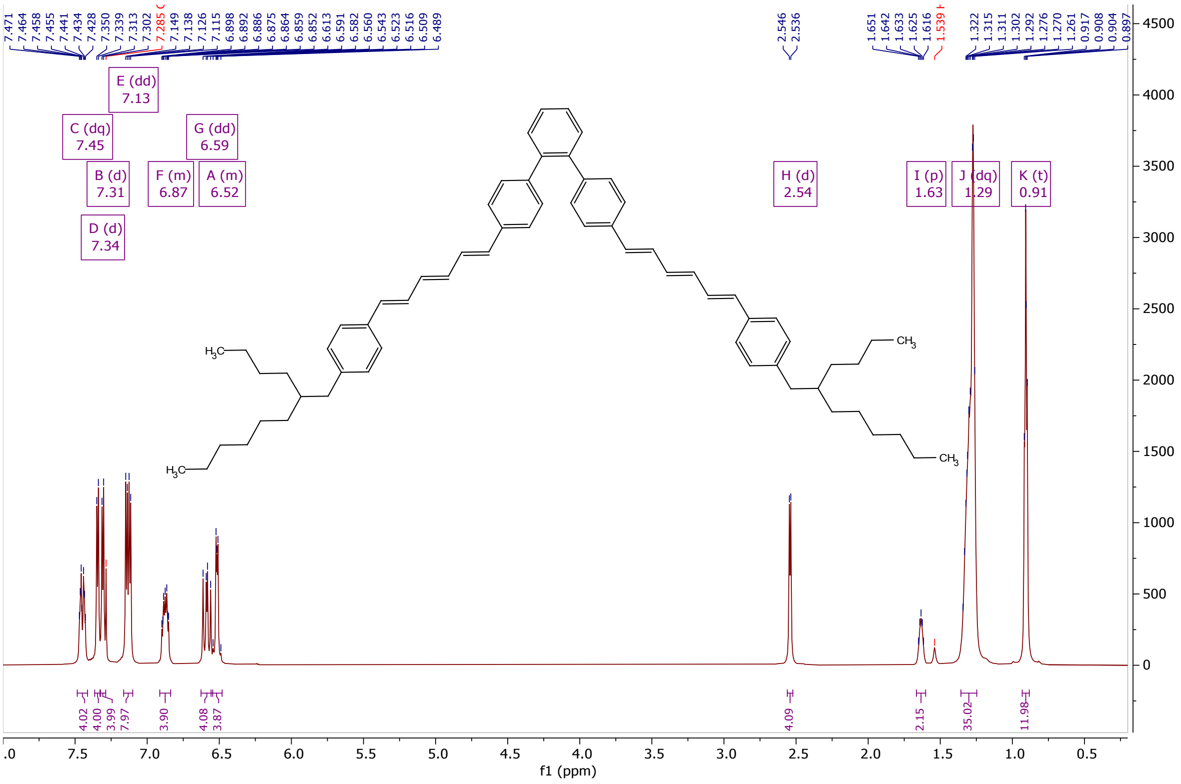

^{13}C NMR, 176 MHz, $CDCl_3$

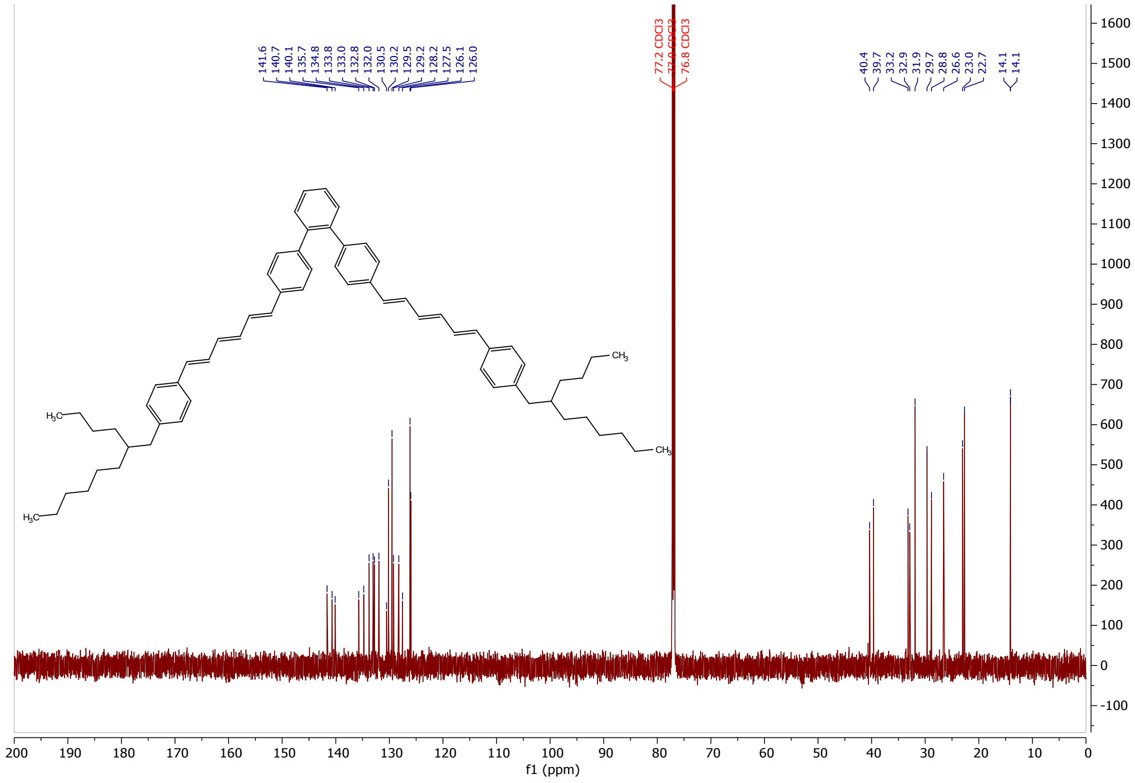

3,3''-bis((1E,3E,5E)-6-(4-(2-ethylhexyl)phenyl)hexa-1,3,5-trien-1-yl)-1,1':3',1''-terphenyl (*m*-(*m*DPH)$_2$)

^{1}H NMR, 400 MHz, CDCl$_3$

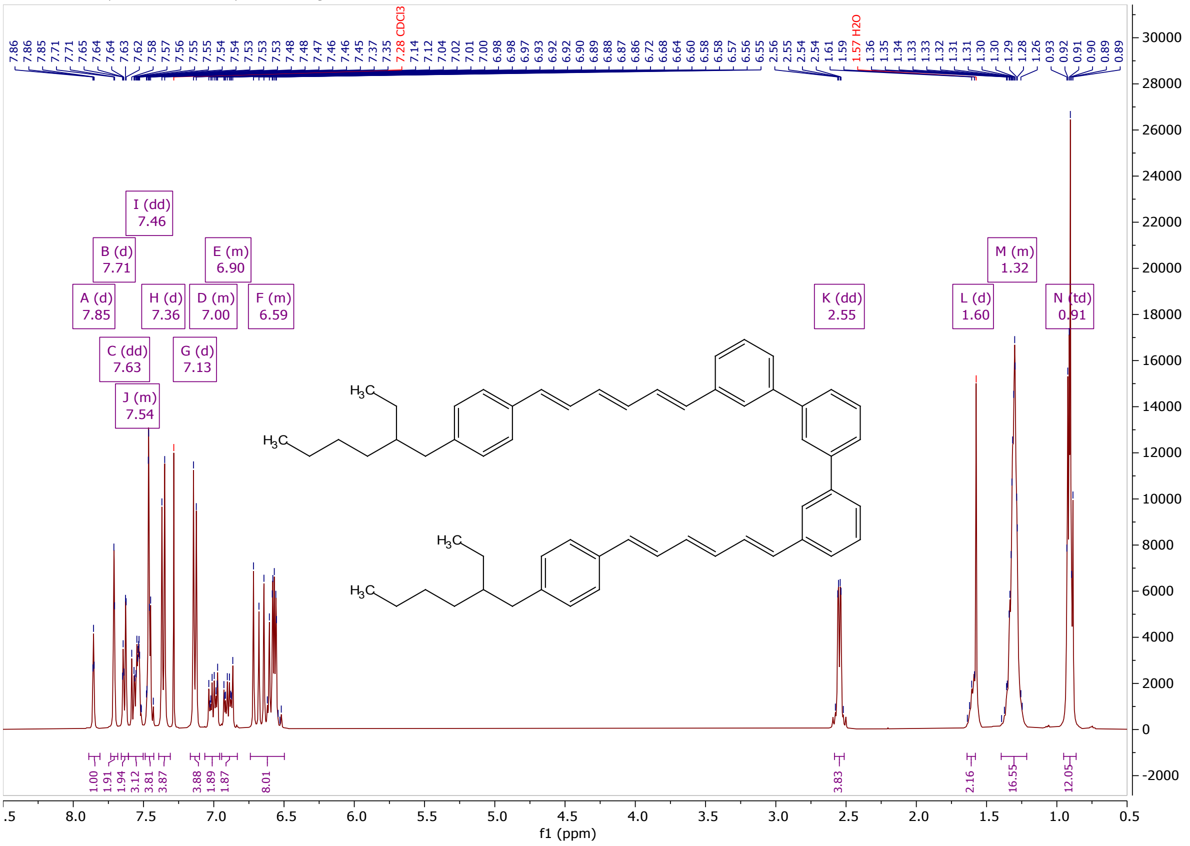

^{13}C NMR, 101 MHz, CDCl$_3$

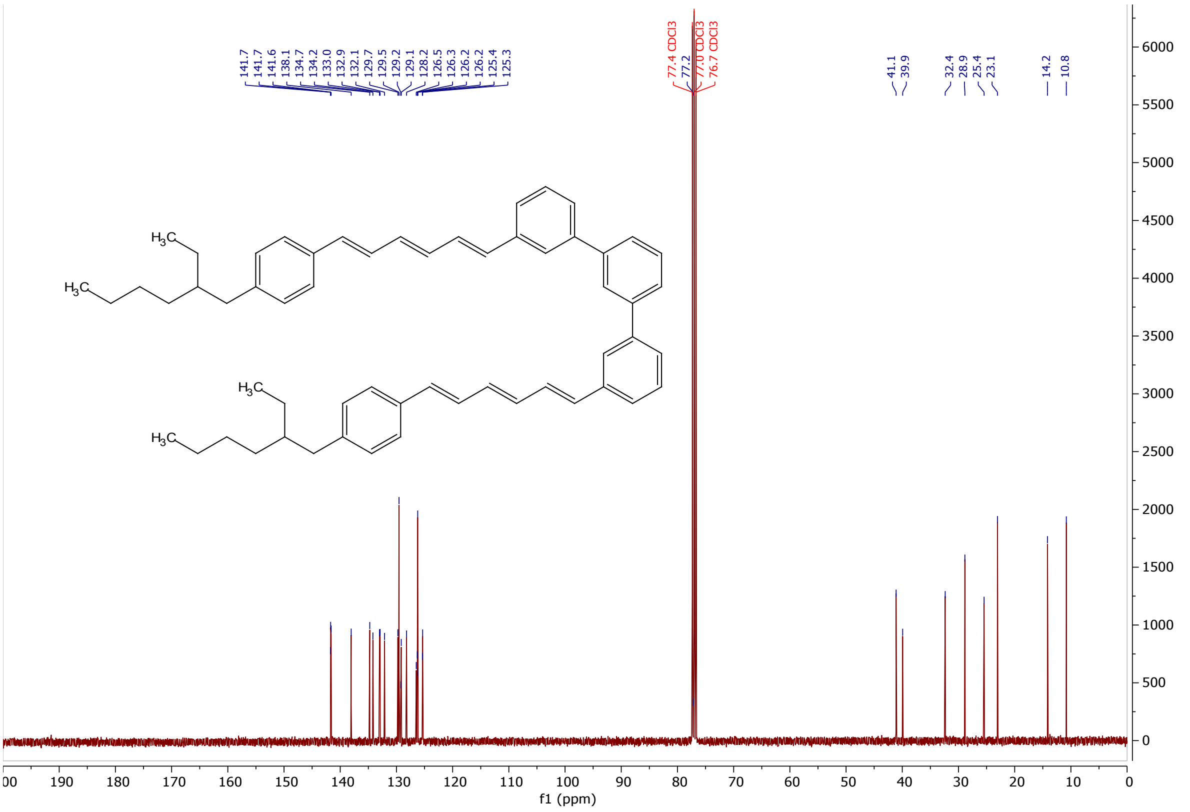

3,3''-bis((1E,3E,5E)-6-(4-(2-ethylhexyl)phenyl)hexa-1,3,5-trien-1-yl)-1,1':4',1''-terphenyl (*p*-(*m*DPH)$_2$)

^{1}H NMR, 500 MHz, $CDCl_3$

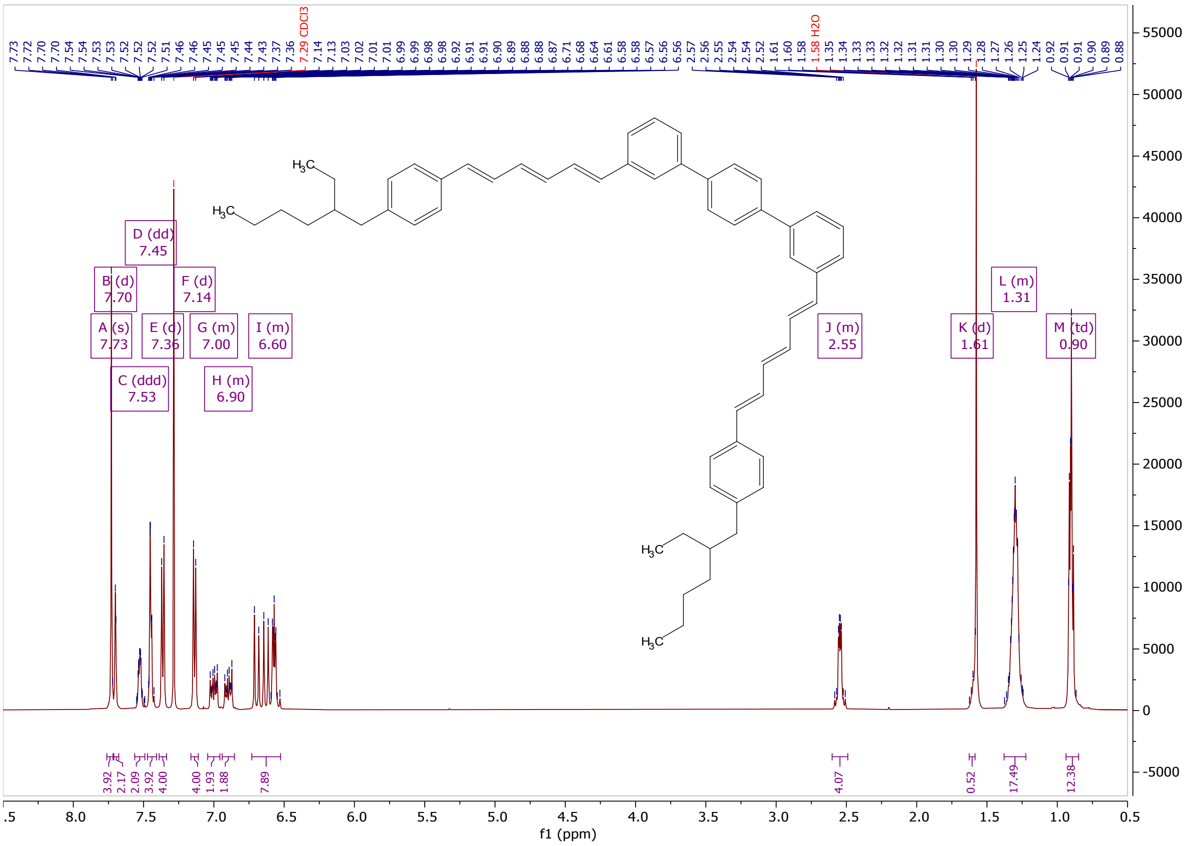

^{13}C NMR, 126 MHz, $CDCl_3$

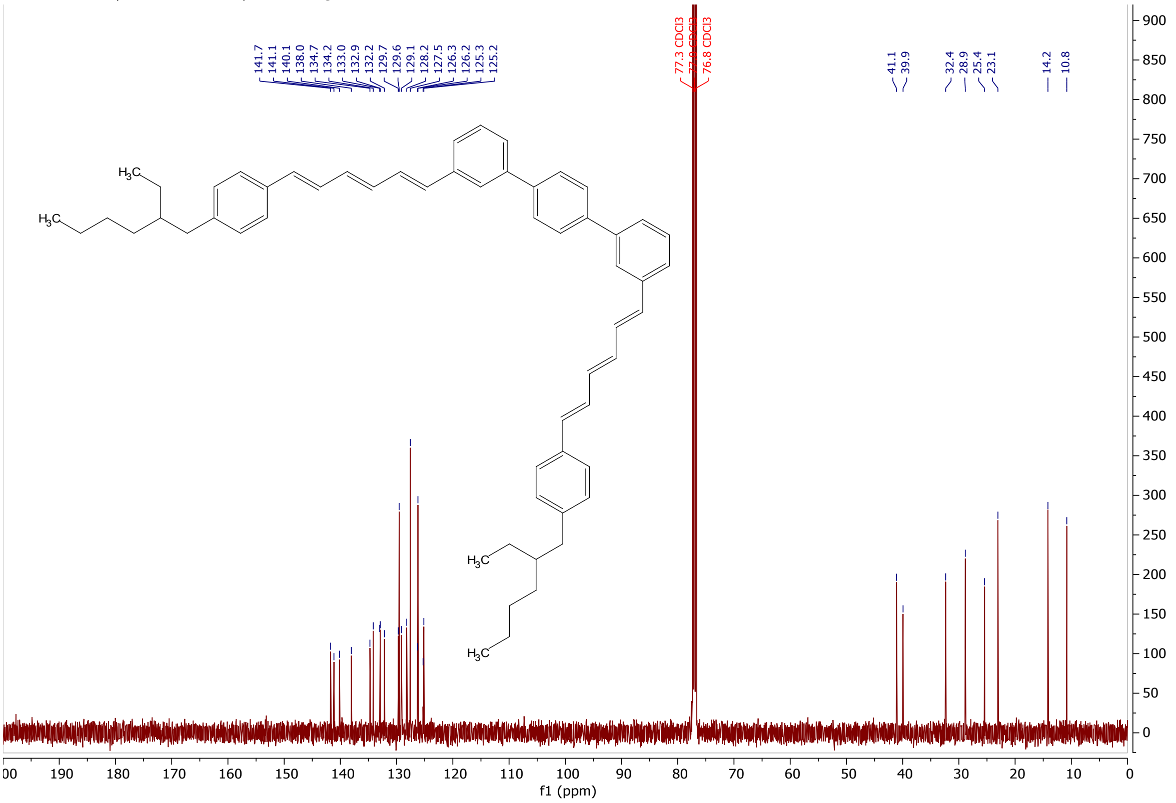

3,3''-bis((1E,3E,5E)-6-(4-(2-ethylhexyl)phenyl)hexa-1,3,5-trien-1-yl)-1,1':2',1''-terphenyl (*o*-(*m*DPH)$_2$)

^{1}H NMR, 400 MHz, $CDCl_3$

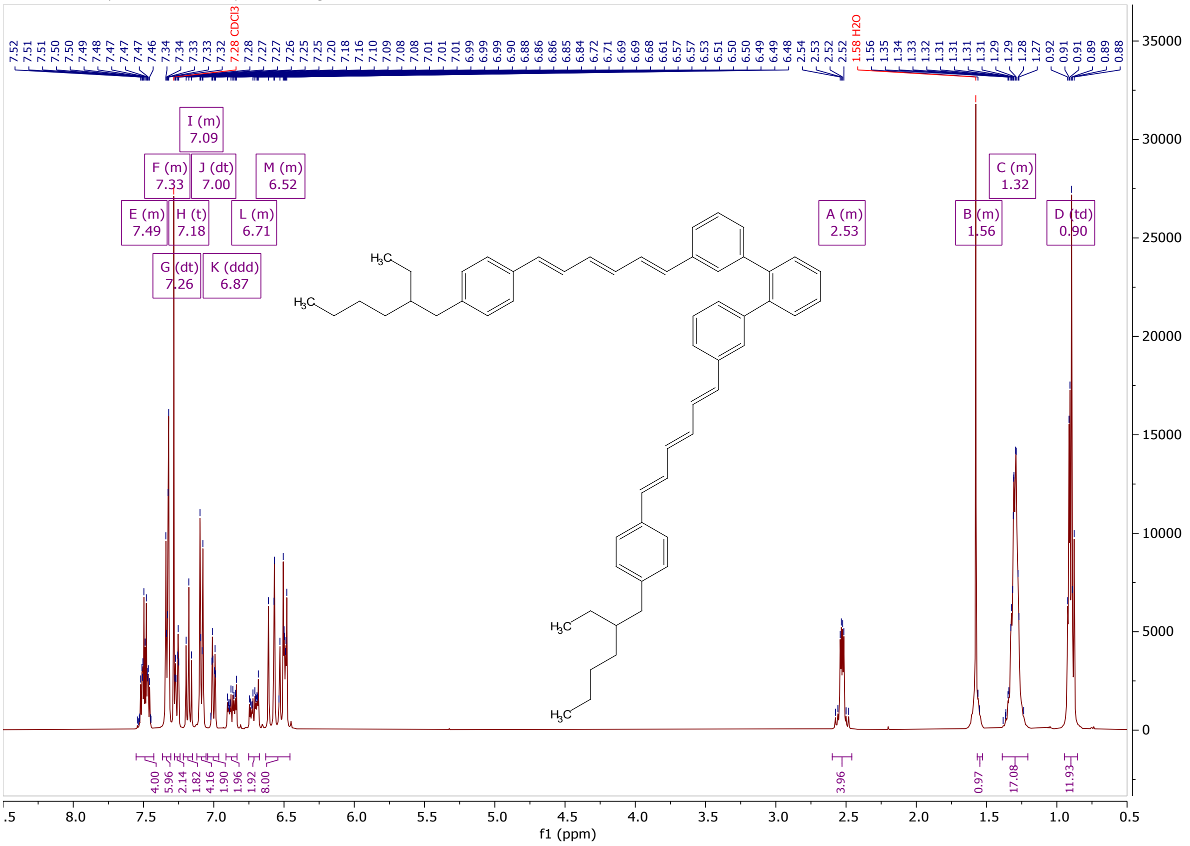

^{13}C NMR, 101 MHz, $CDCl_3$

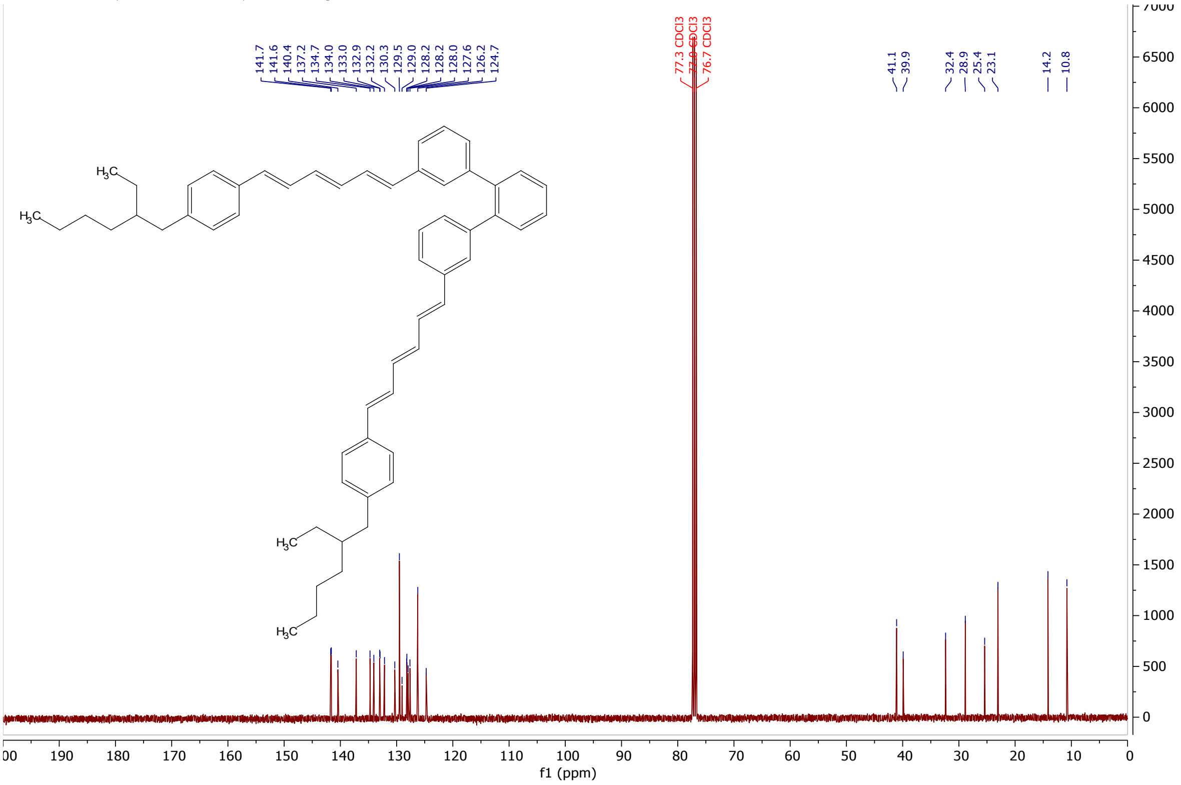

8. References

(1) Chattopadhyay, S. K.; Das, P. K.; Hug, G. L. Photoprocesses in Diphenylpolyenes. Oxygen and Heavy-Atom Enhancement of Triplet Yields. *J Am Chem Soc* **1982**, *104* (17), 4507–4514. https://doi.org/10.1021/ja00381a001.

(2) Chattopadhyay, S. K.; Kumar, C. v.; Das, P. K. Photoprocesses in Diphenylpolyenes. 3. Efficiency of Singlet Oxygen Generation from Oxygen Quenching of Polyene Singlets and Triplets. *J Phys Chem* **1985**, *89* (4), 670–673. https://doi.org/10.1021/j100250a022.

(3) de Mello, J. C.; Wittmann, H. F.; Friend, R. H. An Improved Experimental Determination of External Photoluminescence Quantum Efficiency. *Advanced Materials* **1997**, *9* (3), 230–232. https://doi.org/10.1002/adma.19970090308.

(4) Kubin, R. F.; Fletcher, A. N. Fluorescence Quantum Yields of Some Rhodamine Dyes. *J Lumin* **1982**, *27* (4), 455–462. https://doi.org/10.1016/0022-2313(82)90045-X.

(5) Lunde, K.; Zechmeister, L. Cis-Trans Isomeric 1,6-Diphenylhexatrienes. *J Am Chem Soc* **1954**, *76* (9), 2308–2313. https://doi.org/10.1021/ja01638a008.

(6) Saltiel, J. The *s* - *Trans* , *s* - *Cis* -conformer of All- *Trans* -1,6-diphenyl-1,3,5-hexatriene Was Detected: A Response. *J Phys Org Chem* **2022**. https://doi.org/10.1002/poc.4439.

(7) Saltiel, J.; Wang, S.; Watkins, L. P.; Ko, D.-H. Direct Photoisomerization of the 1,6-Diphenyl-1,3,5-Hexatrienes. Medium Effect on Triplet and Singlet Contributions. *J Phys Chem A* **2000**, *104* (48), 11443–11450. https://doi.org/10.1021/jp002359f.

(8) Dong, S.; Ong, A.; Chi, C. Photochemistry of Various Acene Based Molecules. *Journal of Photochemistry and Photobiology C: Photochemistry Reviews* **2019**, *38*, 27–46. https://doi.org/10.1016/j.jphotochemrev.2018.12.002.

(9) Jousselme, B.; Blanchard, P.; Frère, P.; Roncali, J. Enhancement of the π-Electron Delocalization and Fluorescence Efficiency of 1,6-Diphenyl-1,3,5-Hexatriene by Covalent Rigidification. *Tetrahedron Lett* **2000**, *41* (26), 5057–5061. https://doi.org/10.1016/S0040-4039(00)00792-9.

(10) Gélinas, S.; Paré-Labrosse, O.; Brosseau, C.-N.; Albert-Seifried, S.; McNeill, C. R.; Kirov, K. R.; Howard, I. A.; Leonelli, R.; Friend, R. H.; Silva, C. The Binding Energy of Charge-Transfer Excitons Localized at Polymeric Semiconductor Heterojunctions. *The Journal of Physical Chemistry C* **2011**, *115* (14), 7114–7119. https://doi.org/10.1021/jp200466y.

(11) Walker, B. J.; Musser, A. J.; Beljonne, D.; Friend, R. H. Singlet Exciton Fission in Solution. *Nat Chem* **2013**, *5* (12), 1019–1024. https://doi.org/10.1038/nchem.1801.

(12) Sakai, H.; Inaya, R.; Nagashima, H.; Nakamura, S.; Kobori, Y.; Tkachenko, N. v.; Hasobe, T. Multiexciton Dynamics Depending on Intramolecular Orientations in Pentacene Dimers: Recombination and Dissociation of Correlated Triplet Pairs. *J Phys Chem Lett* **2018**, *9* (12), 3354–3360. https://doi.org/10.1021/acs.jpclett.8b01184.

(13) Skorotetcky, M. S.; Borshchev, O. V.; Cherkaev, G. V.; Ponomarenko, S. A. Synthesis of Nanostructured Organosilicon Luminophores Based on Phenyloxazoles. *Russian Journal of Organic Chemistry* **2019**, *55* (1), 25–41. https://doi.org/10.1134/S1070428019010056.

(14) Chen, W.; Huang, Z.; Wang, W.; Mao, F.; Guan, L.; Tang, Y.; Jiang, H.; Li, J.; Huang, J.; Jiang, L.; Zhu, J. Discovery of New Antimalarial Agents: Second-Generation Dual Inhibitors against FP-2 and PfDHFR via Fragments Assembely. *Bioorg Med Chem* **2017**, *25* (24), 6467–6478. https://doi.org/10.1016/j.bmc.2017.10.017.

(15) Meisner, J. S.; Sedbrook, D. F.; Krikorian, M.; Chen, J.; Sattler, A.; Carnes, M. E.; Murray, C. B.; Steigerwald, M.; Nuckolls, C. Functionalizing Molecular Wires: A Tunable Class of α,ω-Diphenyl-μ,ν-Dicyano-Oligoenes. *Chem Sci* **2012**, *3* (4), 1007. https://doi.org/10.1039/c2sc00770c.

(16) Nomura, S.; Endo-Umeda, K.; Aoyama, A.; Makishima, M.; Hashimoto, Y.; Ishikawa, M. Styrylphenylphthalimides as Novel Transrepression-Selective Liver X Receptor (LXR) Modulators. *ACS Med Chem Lett* **2015**, *6* (8), 902–907. https://doi.org/10.1021/acsmedchemlett.5b00170.

(17) Ramachandran, G. K.; Tomfohr, J. K.; Li, J.; Sankey, O. F.; Zarate, X.; Primak, A.; Terazono, Y.; Moore, T. A.; Moore, A. L.; Gust, D.; Nagahara, L. A.; Lindsay, S. M. Electron Transport Properties of a Carotene Molecule in a Metal-(Single Molecule)-Metal Junction. *Journal of Physical Chemistry B* **2003**, *107* (25), 6162–6169. https://doi.org/10.1021/jp0343786.
